# Novel supramolecular affinity materials based on (−)-isosteviol as molecular templates

**DOI:** 10.3762/bjoc.9.317

**Published:** 2013-12-09

**Authors:** Christina Lohoelter, Malte Brutschy, Daniel Lubczyk, Siegfried R Waldvogel

**Affiliations:** 1 Institute for Organic Chemistry, Johannes Gutenberg University, Duesbergweg 10–14, 55128 Mainz, Germany

**Keywords:** affinity materials, (−)-Isosteviol, supramolecular chemistry, triphenylene ketals, triptycenes, templates

## Abstract

The readily available ex-chiral-pool building block (−)-isosteviol was combined with the *C*_3_-symmetric platforms hexahydroxytriphenylene and hexaaminotriptycene providing large and rigid molecular architectures. Because of the persistent cavities these scaffolds are very potent supramolecular affinity materials for head space analysis by quartz crystal microbalances. The scaffolds serve in particular as templates for tracing air-borne arenes at low concentration. The affinities of the synthesized materials towards different air-borne arenes were determined by 200 MHz quartz crystal microbalances.

## Introduction

Divalent building blocks with a well-defined geometry play a significant role in the construction of highly potent supramolecular structures [1–5]. The rigid nature of such architectures limits the degrees of freedom and guarantees a good preorganization [1–2,6–10]. Particular interest was given to *C*_3_-symmetric structures, serving e.g. as templates in asymmetric catalysis or molecular recognition [11–14].

**Figure 1:**
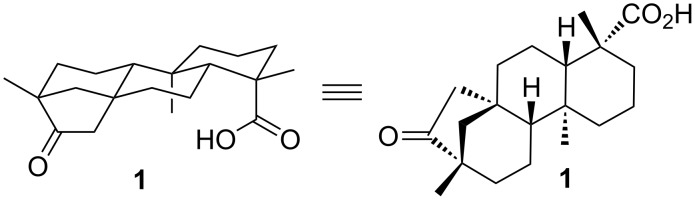
Unique structural features of (−)-isosteviol.

**Figure 2:**
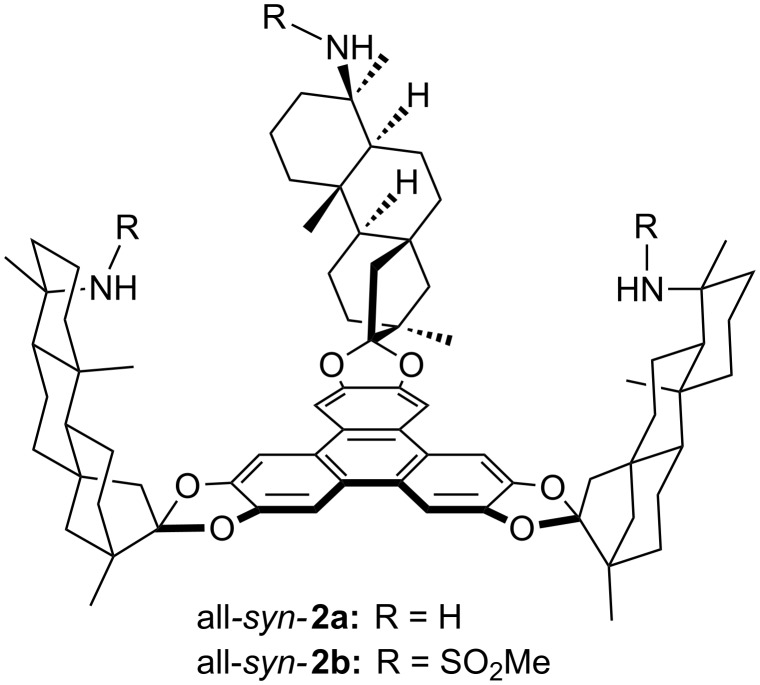
Triphenylene ketal based on (−)-isosteviol.

A specific but potent subclass of such *C*_3_-symmetric architectures is represented by triphenylene ketals [15]. They have found significant application as receptors and chemical sensors in the detection of aromatic compounds [16–20]. Thus, the first artificial receptor for caffeine had been established [21–22]. The introduction of chiral information onto a supramolecular entity gave rise for enantiofacial differentiation of a single substrate [23–24]. Due to the concave arrangement of both functional groups, exhibiting a distance of the two carbonyl carbon atoms of about 7 Å, naturally occurring diterpene (−)-isosteviol **1** [25] came into focus as building block for the construction of such receptor geometries (Figure 1) [26].

(−)-Isosteviol **1** can be easily obtained on large scale by acidic treatment of stevioside [27–28]. This stevioside is a commercially available natural sweetener which is isolated from *stevia rebaudiana* by alcoholic extraction [29–30]. In addition to their cytotoxic activities [31–34], compounds based on (−)-isosteviol have found application in various fields of synthetic chemistry [35], including the construction of tweezer-like supramolecular transporters for amino acids [36], chiral organocatalysts in aldol reactions [37], or the complex formation with aromatic compounds [38–39], Within in a nine-step synthesis triphenylene ketals based on (−)-isosteviol were prepared [40]. Receptor structures **2** equipped with amino or sulfonylamido functionalities were obtained (Figure 2).

**Figure 3:**
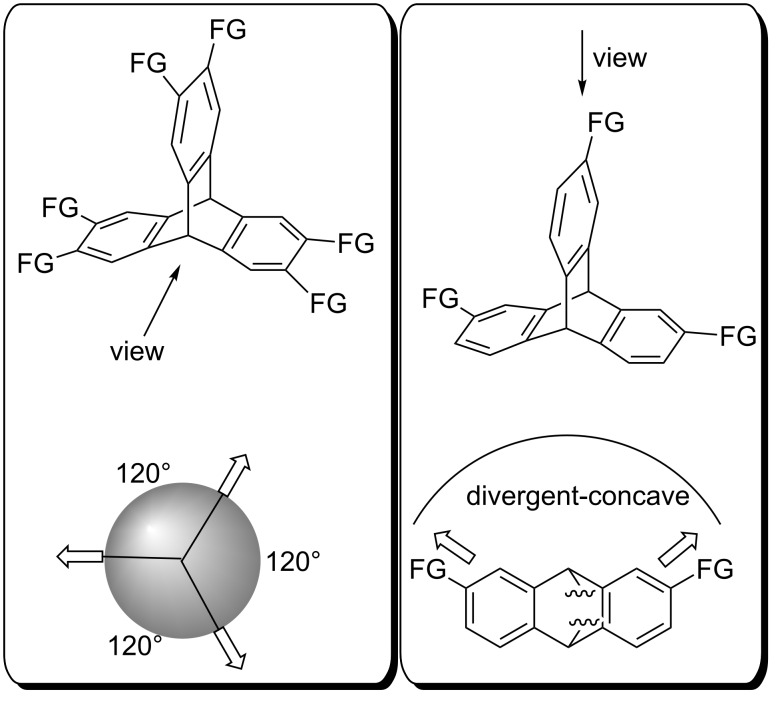
Structural features of triptycene derivatives.

In addition to triphenylene ketals, triptycene-based structures also exhibit the geometrical requirements for the formation of *C*_3_-symmetric architectures with extended cavities. Due to their structural features, triptycenes have found widespread application in organic synthesis: Ranging from polymer sciences [41], materials for gas storage [42–50], (organo)catalysis [51–52], molecular machinery [53–54] and supramolecular host–guest chemistry [55–56]. With an angle of 120° between its aromatic moieties, it exhibits a rigid geometry with a defined alignment of functional groups (Figure 3, left). Next to classical convergent–concave structures, architectures with a divergent–concave arrangement of functionalities can be obtained upon installation of building blocks with a linear geometry (Figure 3, right).

Here, the functionalization of (−)-isosteviol-based triphenylene ketal all*-syn****-*2a** as well as the combination of (−)-isosteviol with the triptycene platform is reported. The application of these novel architectures as supramolecular affinity materials was studied.

The investigations of these possible affinity materials were carried out with 200 MHz high fundamental frequency quartz crystal microbalances (HFF-QCMs) via gravimetric sensing of the adsorbed analytes. The advantage of HFF-QCMs is the low detection limit and the fast, highly reproducible and easy to apply electro spray protocol for the coating of such devices [57–59]. Almost all organic materials which show at least a certain solubility in tetrahydrofuran or other volatile organic solvents allow application of this versatile coating protocol. For such studies only small amounts of affinity material in the sub mg-range are required.

The frequency shifts of the coated QCMs were determined for different analyte concentrations. Subsequently, the constants of the Langmuir isotherm are obtained by fitting the frequency shifts over concentration. The affinity of an affinity material to an analyte is calculated by multiplication of these constants. Details about the experimental setup and the determination of the affinities are given in Supporting Information File 1.

## Results and Discussion

### Triphenylene ketals

**Scheme 1:**
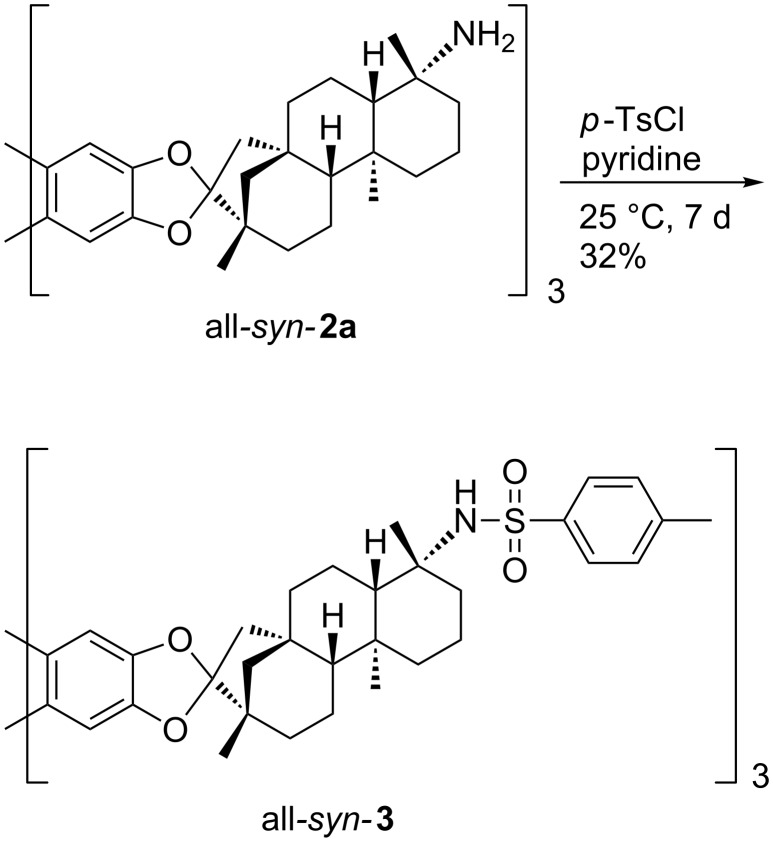
Functionalization of triphenylene ketal **2a**.

The high hydrogen bonding donor capability of sulfonamides made this class of functional groups highly attractive for supramolecular affinity materials [60]. The installation of larger substituents at the sulfonyl moiety should provide suitable properties for the processing on the quartz crystal microbalances. Based on the reported triamine all*-syn****-*2a** [40], **2a** was brought to reaction with *para*-toluenesulfonyl chloride in order to equip the receptor scaffold with sulfonamide binding sites (Scheme 1).

The reaction proceeds with a moderate yield of 32% under very mild reaction conditions. The high steric demand in vicinity of the amino functions requires these prolonged reaction times. Unfortunately, more drastic reaction conditions lead to degradation of the substrate.

### Triptycenes

**Figure 4:**
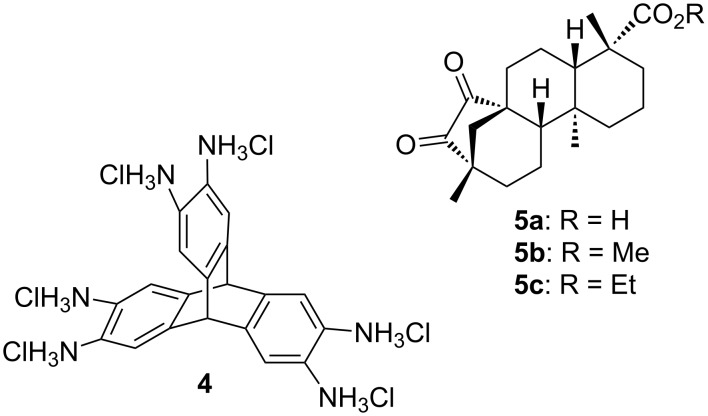
Hexaammoniumtriptycene hexachloride **4** and 15-oxo-derivatives **5a**–**c** of (–)-isosteviol.

Recently, the synthesis of hexaammoniumtriptycene hexachloride **4** (Figure 4) was reported by Mastalerz et al. [61]. Further, (−)-isosteviol and its esters can be converted into the corresponding 1,2-diketones **5a**–**c** via Riley oxidation [62–66]. The linkage of the two building blocks by condensation reaction of the keto with the amino functionalities presents a promising route for the construction of novel (−)-isosteviol-based *C*_3_-symmetric scaffolds.

**Scheme 2:**
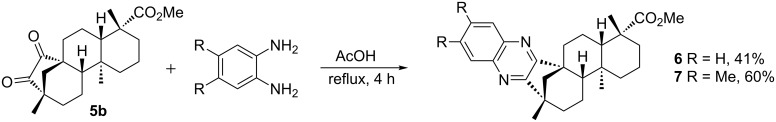
Quinoxalines based on diketone **5b**.

For the elaboration of optimal condensation conditions to form quinoxalines and to obtain model compounds representing subunits for the triptycene architectures *o*-phenylenediamines were employed (Scheme 2). It turned out that **5b** forms the corresponding quinoxaline derivatives **6** and **7** in acceptable yields of 41% and 60%, respectively, when refluxing in glacial acetic acid.

**Scheme 3:**
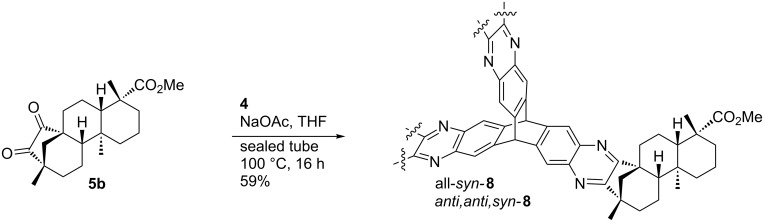
Condensation of **5b** with hexaammoniumtriptycene hexachloride.

**Figure 5:**
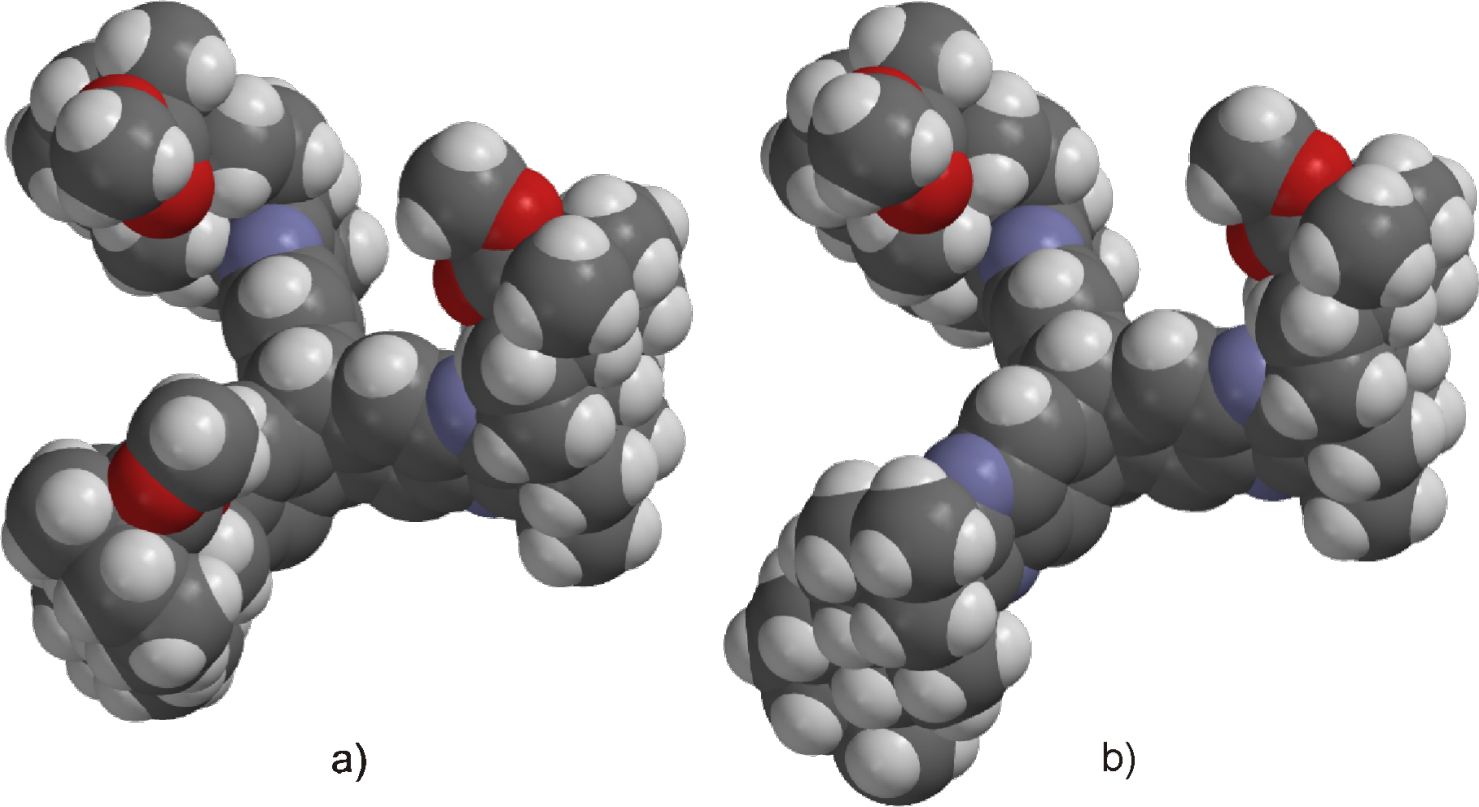
Molecular modelling structures (Spartan ’08 V1.0.0) of (a) all*-syn*-**8** and (b) *anti,anti,syn*-**8**.

The established reaction conditions failed on the *C*_3_-symmetric platform due to the poor solubility of the starting materials. Switching to a protocol with sodium acetate as additional base and operation in a sealed tube provided a satisfactory yield of **8** (Scheme 3) [61]. By molecular modelling the structural features can be visualized (Figure 5). A relatively closed *C*_3_-symmetric cleft is formed by all*-syn*-**8** (Figure 5a), whereas the less symmetric *anti,anti,syn*-**8** (Figure 5b) provides an open side to access the concave regime.

**Scheme 4:**
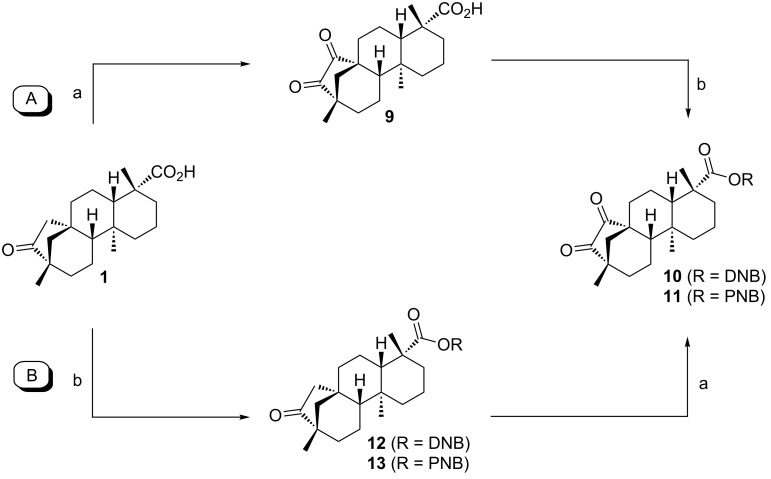
Synthesis of nitrobenzylic ester derivatives **10** and **11**, starting from (−)-isosteviol **1**.

As anticipated, two stereoisomers are formed in this condensation reaction of **5b** with **4**. After separation via column chromatography, these products were identified as the all-*syn-* and *anti,anti,syn-*isomers of the desired product **8**, which were obtained in a total yield of 59%. Due to very similar polarity of both isomers, the separation turned out to be tedious. Thus, pure *anti,anti,syn-***8** and all*-syn-***8** were isolated in yields of only 25% and 3%, respectively, with the remaining 31% as mixture of isomers. In order to facilitate the separation of isomers the ester moiety of (−)-isosteviol was converted into nitrobenzylic esters. The highly polar nitro groups at the periphery of the resulting triptycene isomers should provide the desired difference in polarity. Another feature of the employment of benzyl derivatives is the facile cleavage under reductive conditions representing a versatile precursor for further modifications. Starting from (−)-isosteviol **1**, there are two ways of obtaining the diketo building blocks **10** and **11**, bearing a dinitrobenzylic (DNB) and a *p*-nitrobenzylic (PNB) ester moiety, respectively (Scheme 4).

**Scheme 5:**
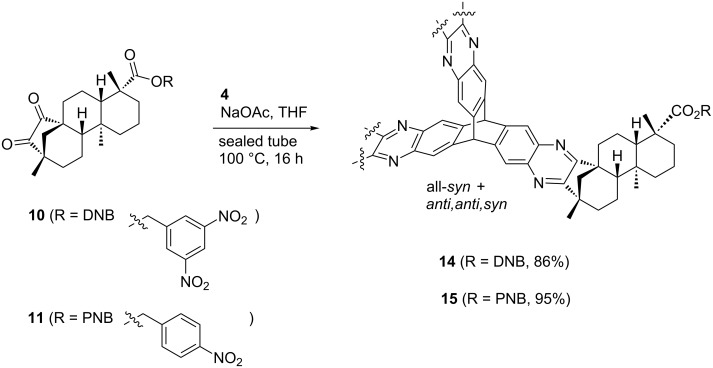
Condensation of the nitrobenzyl esters **10** and **11** with hexaammoniumtriptycene hexachloride **4**.

Following path **A**, (−)-isosteviol can be oxidized under Riley conditions (Table 1, reaction conditions **a**: selenium dioxide/xylene) [62–63] to give the corresponding diketone **9** [66]. Subsequent esterification with 3,5-dinitrobenzylic chloride under basic conditions (Table 1, reaction conditions **b** for R = DNB) [67] proceeds with a yield of 57% and leads to the formation of **10** with an overall yield of 46%. Alternatively, protection of the carboxylic acid function of (−)-isosteviol can be carried out first (path **B**), yielding dinitrobenzyl ester **12**. Subsequent Riley-oxidation renders the desired product **10** in an overall yield of 48%. Since path **A** and path **B** both proceed with almost identical yields, either pathway is suitable for the preparation of such protected 1,2-diketones. Apart from applying 3,5-dinitrobenzyl chloride in the reaction sequence, the conversion of the carboxylic acids **1** and **9** with 4-nitrobenzyl (PNB) chloride was carried out (Scheme 4). Again, both paths **A** and **B** lead to the formation of the desired PNB protected diketone **11**. Esterification was achieved by reaction with 4-nitrobenzyl chloride and cesium carbonate in DMF (Table 1, reaction conditions **b** for R = PNB) [68]. Both sequences **A** and **B** proceed with similar overall yields as well, rendering **11** in 54% and 57%, respectively. Upon employing 4-nitrobenzyl chloride, both reaction paths result in slightly higher yields than the analogous reactions with 3,5-dinitrobenzyl chloride. Diketones **10** and **11** were then brought to condensation with hexaammoniumtriptycene hexachloride **4**, following the protocol mentioned above (Scheme 5). Reaction of the 3,5-dinitrobenzyl ester **10** with **4** renders the corresponding triptycene structure **14** as a mixture of isomers in a good total yield of 86%. However, the impact of the nitro groups at the periphery of the molecule on the polarity of the isomers was far less than anticipated. After twofold column chromatography, the *anti,anti,syn-*isomer was isolated in a yield of 48% while the *all-syn-*isomer was obtained in 17% yield, again leaving the rest as a mixture of isomers.

**Table 1:** Oxidation and esterification sequence of **1**.

R =	Reaction conditions	Yield	Overall yield a + b

DNB 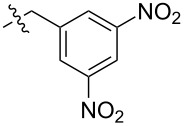	a: SeO_2_, xylenes, 145 °C, 2 d	**9**: 80% **10**: 78%	Path **A**: 46%
	b: 3,5-dinitrobenzyl chloride, NEt_3_, DMF, 25 °C, 16 h	**12**: 61% **10**: 57%	Path **B**: 48%
PNB 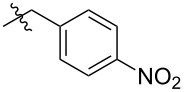	a: SeO_2_, xylenes, 145 °C, 2 d	**9**: 80% **11**: 74%	Path **A**: 54%
	b: 4-nitrobenzyl chloride, Cs_2_CO_3_, DMF, 25 °C, 5 h	**13**: 77% **11**: 68%	Path **B**: 57%

Reaction of 4-nitrobenzyl ester **11** with **4** leads to the formation of the corresponding triptycenes *anti,anti,syn-***15** and all*-syn****-*15** in an excellent total yield of 95%. Separation of the isomers results in the isolation of 34% of the *anti,anti,syn-*isomer and 12% of the all*-syn-*isomer, leaving the remaining 49% as a mixture of isomers.

**Scheme 6:**
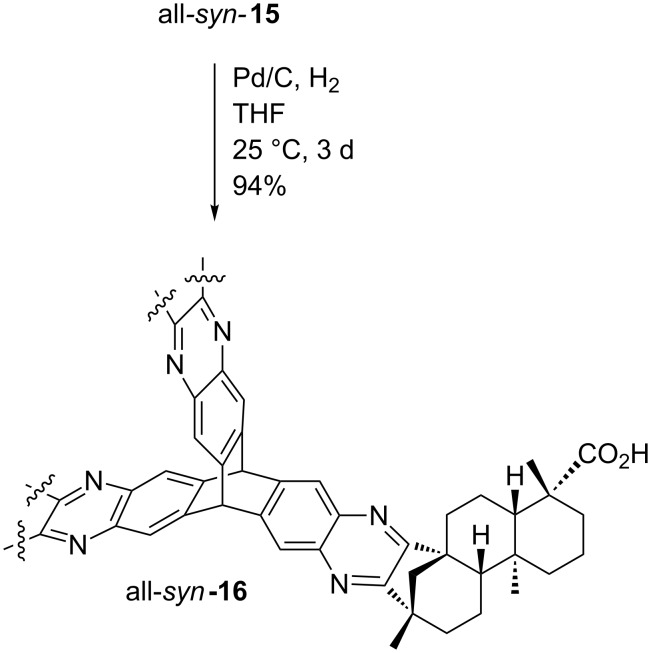
Hydrogenolysis to tricarboxylic acid all-*syn-***16**.

The benzyl ester moiety of all*-syn****-*15** was subsequently cleaved by hydrogenolysis using standard conditions (Scheme 6) [69]. The tricarboxylic acid all*-syn-***16** was isolated in almost quantitative yield. In this protocol the heterogeneous catalyst did not affect the quinoxaline moieties by over-reduction.

**Scheme 7:**
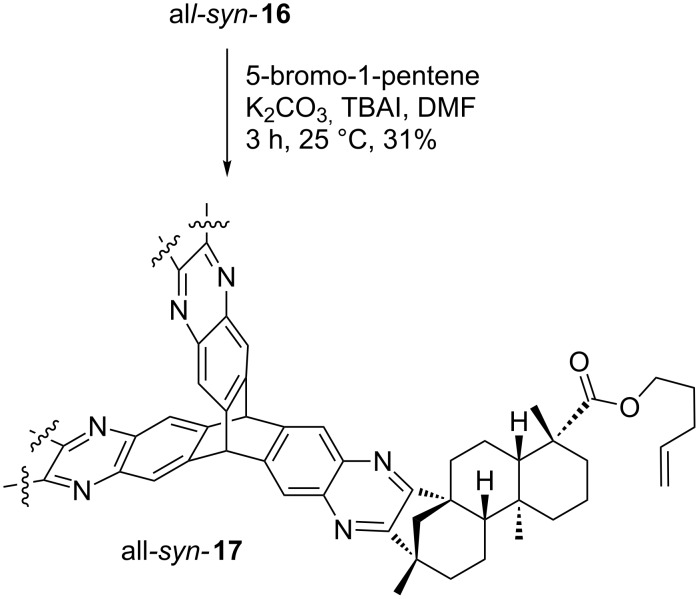
Alkylation of all*-syn-***16** renders terminal alkene all*-syn-***17**.

With this versatile precursor all*-syn-***16** in hands and the need for organic materials with pronounced cavities as potent affinity materials we envisioned the synthesis of a capsule-type architecture. Therefore, all*-syn-***16** was *O*-alkylated by treatment with 5-bromo-1-pentene using standard conditions [70] to yield the corresponding triptycene derivative all*-syn-***17** (Scheme 7). Alkylation proceeded with a moderate yield of 31%. Equipped with three terminal alkenes all-*syn-***17** seems to be a suitable precursor for subsequent olefin metathesis to form a capsule-like structure.

**Figure 6:**
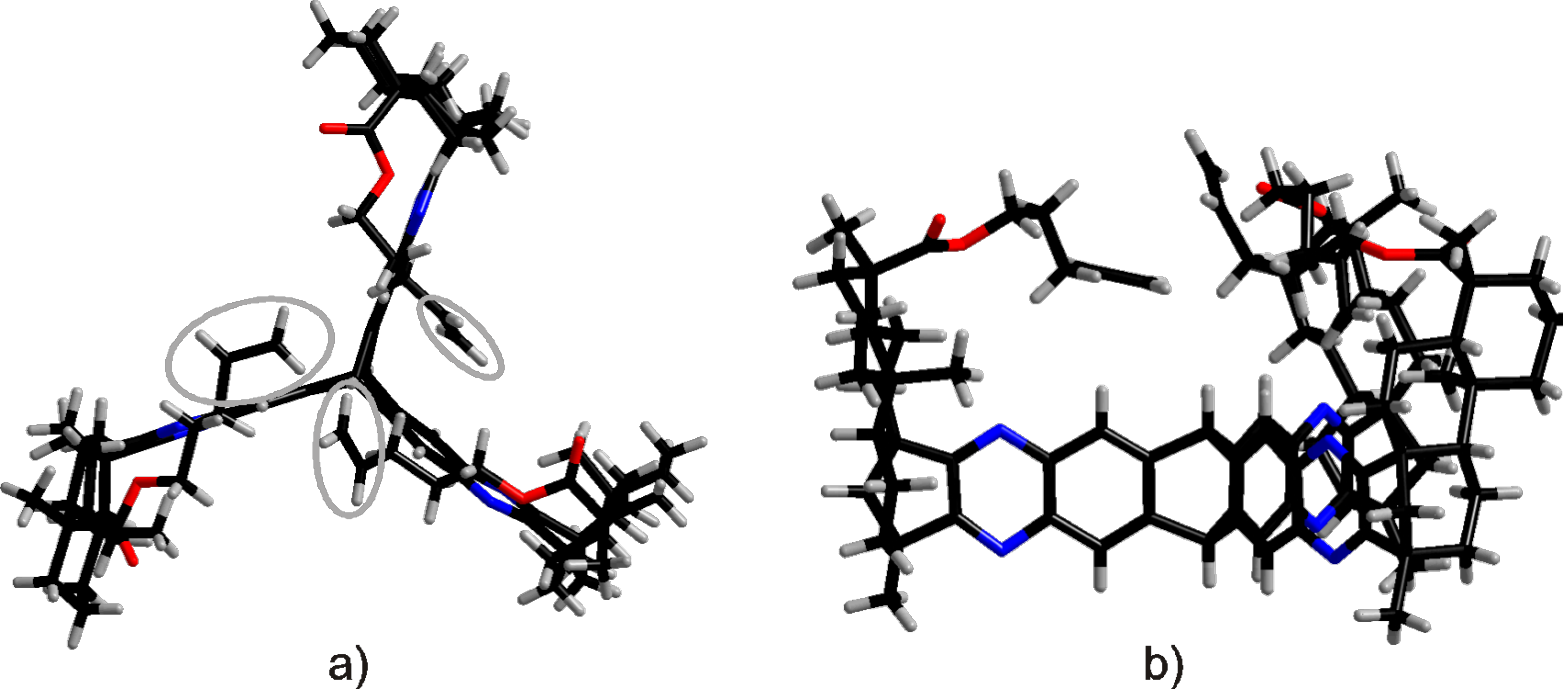
(a) Top view on the molecular structure of all*-syn-***17** with the terminal alkene fragments labelled in grey; (b) side view of all*-syn-***17**.

The structure of all*-syn-***17** was confirmed via X-ray analysis of a suitable single crystal. The molecular structure clearly reveals the spatial arrangement of the terminal alkene moieties. The side view of all-*syn-***17** (Figure 6b) unequivocally shows the cavity which is formed, its size ranging approximately to the quinoxaline-nitrogen atoms of the triptycene core which exhibit a mutual distance of 8.1 Å, 8.8 Å and 8.9 Å, whereas the carboxylic carbon atoms exhibit a distance of about 12 Å. The cavity of all-*syn-***17** is “roofed” by the alkyl chains of the (−)-isosteviol units (Figure 6a), whose terminal carbon atoms show a mutual distance in the range of 4.2–5.2 Å.

**Scheme 8:**
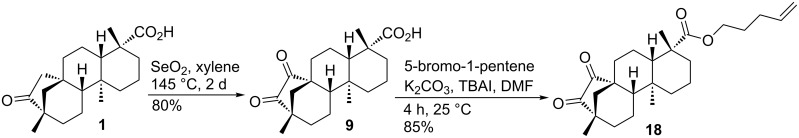
Alkylation of (−)-isosteviol diketone **9** with 5-bromo-1-pentene.

Since the introduction of nitrobenzyl esters as protecting groups for the (−)-isosteviol carboxylic acid moiety obviously does not have an enhanced effect on the polarity of the two isomers and, as a consequence, the chromatographic separation procedure does not correlate with the nature of the ester substituent, the detour via protecting groups in the synthesis of alkylated triptycene **17** was subsequently avoided. Therefore, alkylated diketone **18** was synthesized, starting from (−)-isosteviol (Scheme 8). Since alkenes are known to participate in Riley oxidations as well, rendering allylic alcohols, there is only one route to **18**. After Riley oxidation of **1**, alkylation was carried out under the same reaction conditions as before, providing the desired product in a yield of 85% [70].

**Scheme 9:**
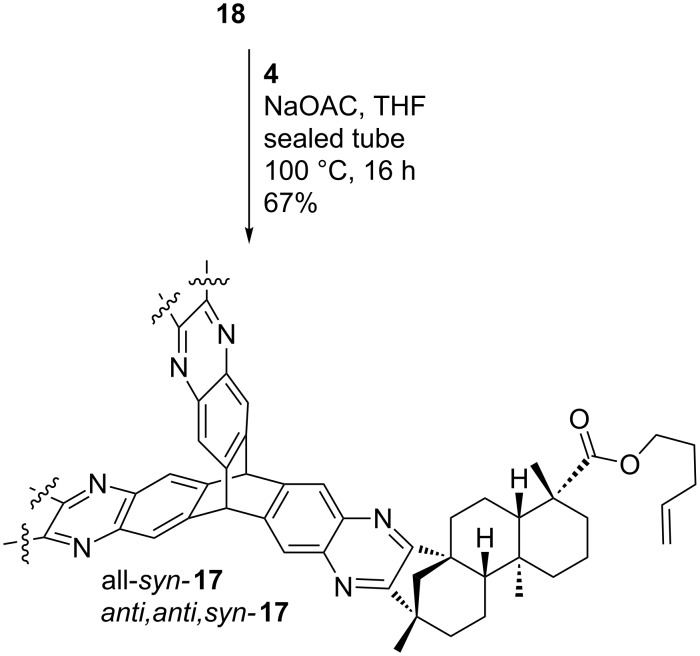
Direct synthesis of alkylated triptycene **17** by condensation of **18** with hexaammoniumtriptycene hexachloride **4**.

**18** was then brought to reaction with hexaammoniumtriptycene hexachloride **4** (Scheme 9). The corresponding all*-syn-* and *anti,anti,syn-*isomers of **17** were obtained in an overall yield of 67%.

**Scheme 10:**
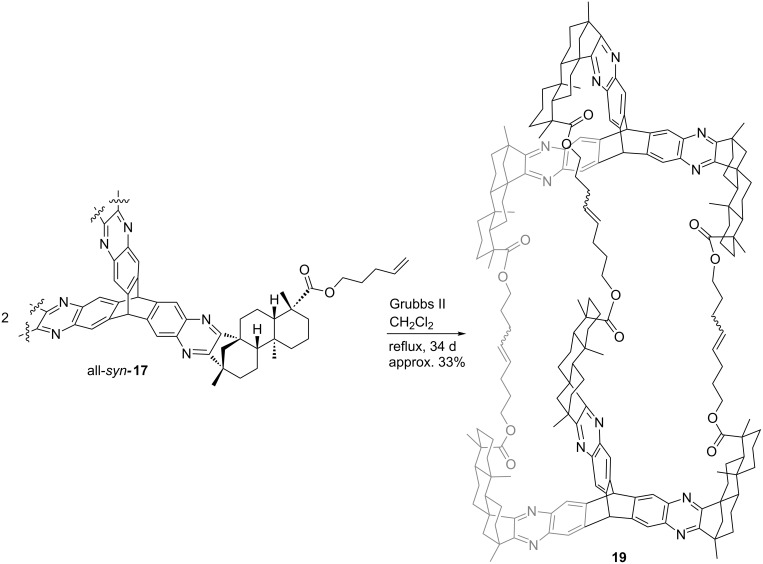
Olefin metathesis of all*-syn-***17**.

After twofold column chromatography, complete separation of the isomers was achieved. The *anti,anti,syn*-derivative was isolated in a yield of 44% whereas the all-*syn*-derivative was obtained in 23% yield. The molecular structure of all-*syn-***17** depicted in Figure 6 provides the rationale for the ameliorated separation of the isomers. The protection of one side with alkenyl esters blocks the access of the quinoxaline moieties from one side. The all*-syn* derivatives will show a better interaction with the stationary phase. The combination of entire isomer separation and shortcut in the reaction sequence (three steps instead of five, starting from (−)-isosteviol) makes this a highly attractive route for the construction of the desired triptycene derivatives. All*-syn-***17** was then brought to reaction in an olefin metathesis (Scheme 10), employing the 2^nd^ generation Grubbs catalyst in refluxing dichloromethane.

**Figure 7:**
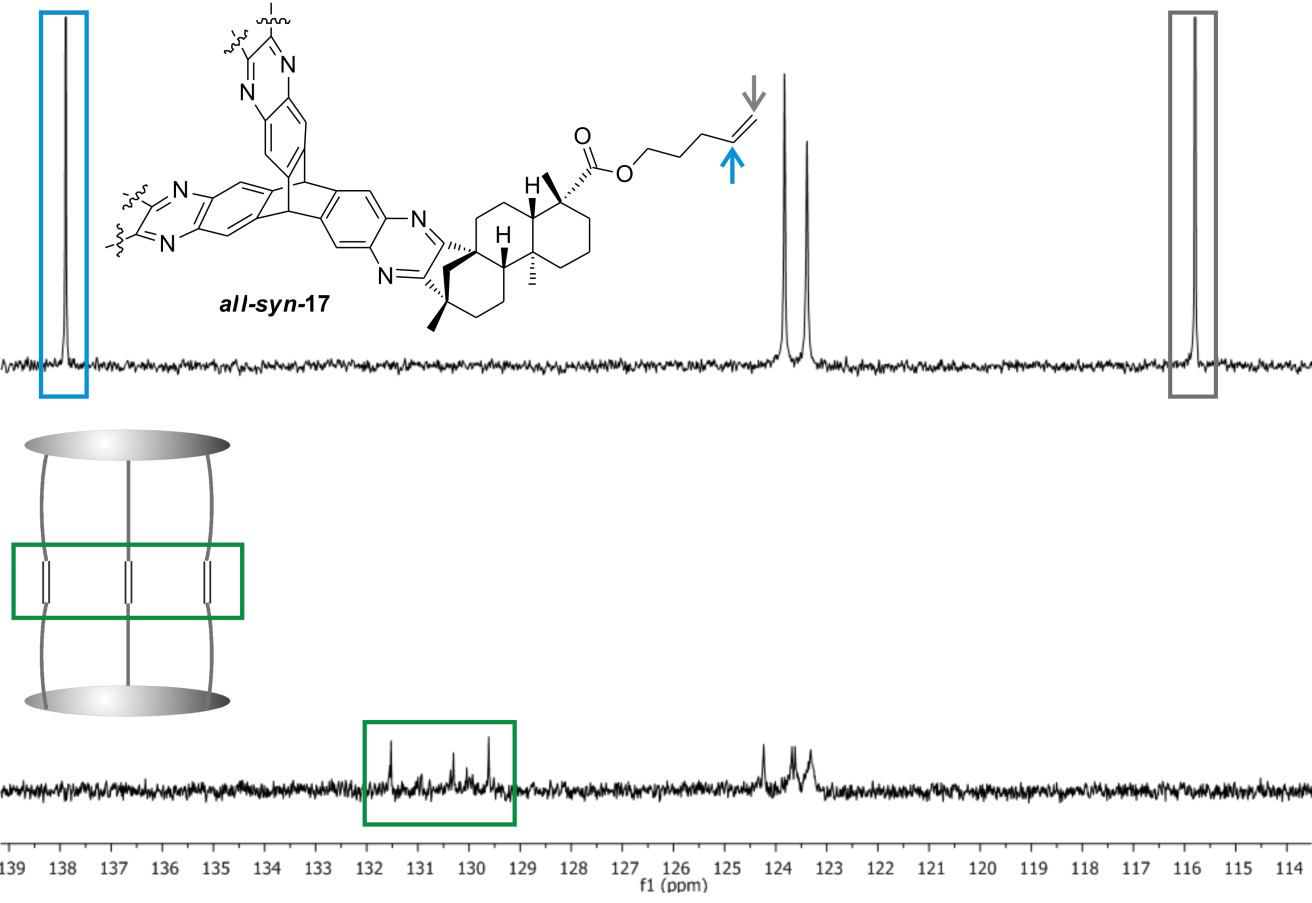
Extracts of the ^13^C NMR spectra of starting material and product.

Even after prolonged reaction times, complete conversion of the starting material could not be achieved. However, the formation of a sole product was observed. Upon chromatographic separation, the product was obtained, exhibiting a molecular weight corresponding to the cage-like structure **19**. Comparison of according sections from the carbon NMR spectra of starting material and product shows that the signal which corresponds to the terminal carbon atom in the starting material (Figure 7, grey) is not existent in the product spectrum. Furthermore, a new set of signals (Figure 7, green) can be seen in the product spectrum which show typical shifts of 1,2-disubstituted alkenes.

**Figure 8:**
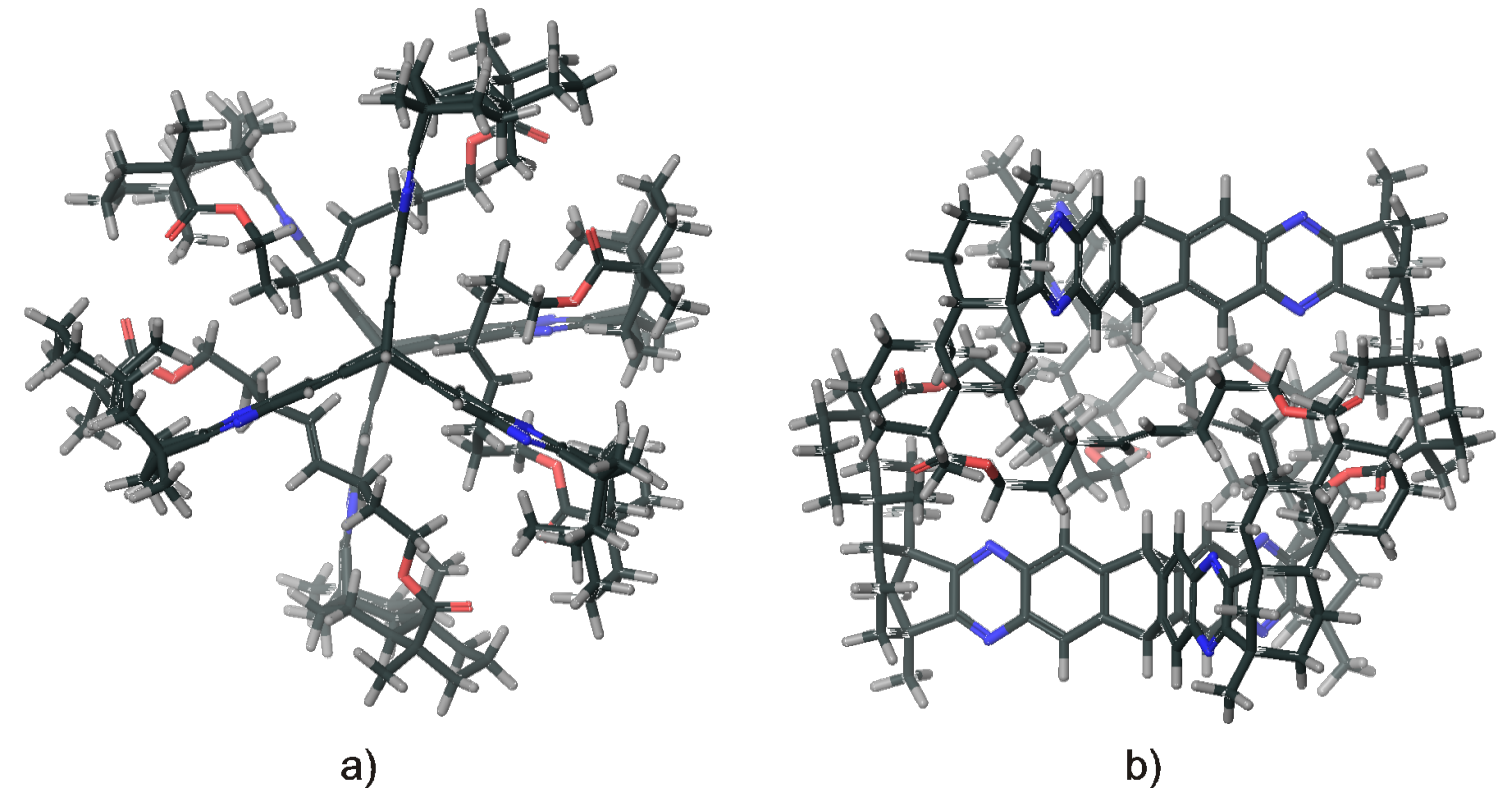
Molecular modelling structure (MacroModel 9.3.5) of collapsed **19**, (a) top view; (b) side view.

Since the obtained analytical data strongly indicate the existence of cage compound **19**, molecular modelling calculations (Figure 8, MacroModel 9.3.5) indicate a “collapsed” structure in which the triptycene units are twisted by 60° and approaching each other, prohibiting the formation of significant voids for intercalation of analytes.

### Results of the gravimetric measurements with HFF-QCMs

Triphenylene ketal **3** and the triptycene derivatives **8**, **14**, **15**, **17, 19** and the model compound **7** were subjected to QCM measurements in order to evaluate their properties to serve as affinity materials in the tracing of volatile aromatic compounds. For this purpose, a collection of chemically related aromatic analytes were screened. The analytes range from benzene to pseudocumene, exhibiting similar electronic properties but differing in their size. This allows a probing of cavity size of the individual affinity materials.

**Figure 9:**
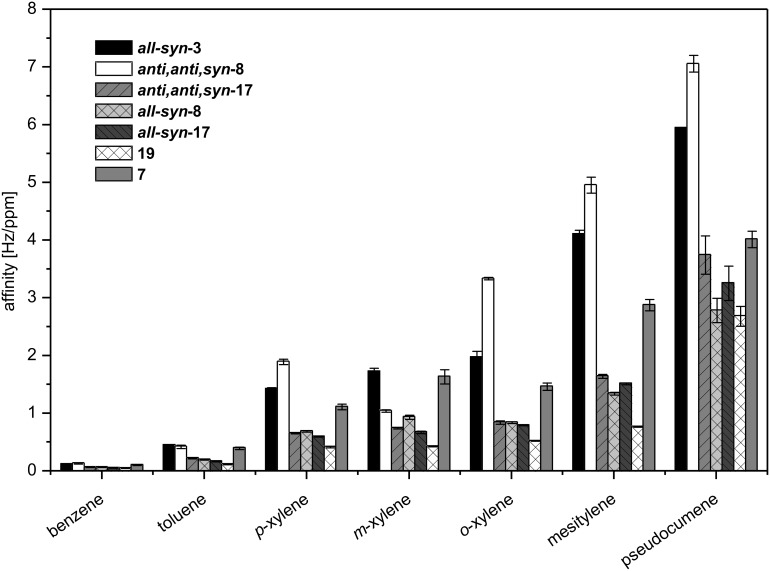
Screening of aromatic analytes with affinity materials **3**, **7**, **8**, **17** and **19**.

Triptycene based material *anti,anti,syn-***8** shows the highest affinity towards aromatic analytes, which can be rationalized with a more facile access of the analyte to the void created by the *anti,anti,syn-*isomer (Figure 9).

**Figure 10:**
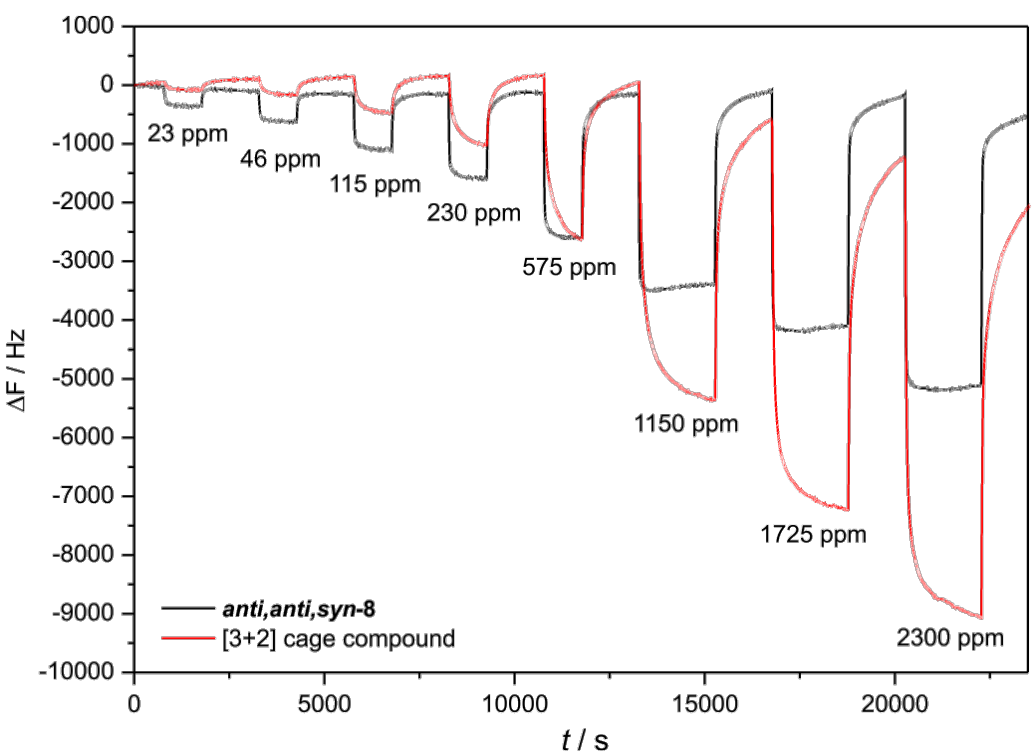
Primary data of *anti,anti,syn*-**8** and a [3 + 2] cage compound (increasing pseudocumene concentrations and recovery times between each step, for details see Supporting Information File 1).

This remarkable effect can already be observed when plotting the primary data (Figure 10).

For comparison, we included the primary data of an organic cage compound (for structure see Supporting Information File 1) which was published recently [71]. At small concentrations which are relevant for tracing, *anti,anti,syn*-**8** is creating unusual large signals which indicates a high affinity of the material for this analyte. For high concentrations, other materials like the [3 + 2] cage compound get ahead in means of signal depth, indicating more places for adsorption on/in the film. This behavior of *anti,anti,syn*-**8** is exclusively observed for the aromatic analytes. Our triptycene-based cage compound **19** exhibits only moderate affinities, which is in accordance with the calculated collapsed structure (Figure 8).

**Figure 11:**
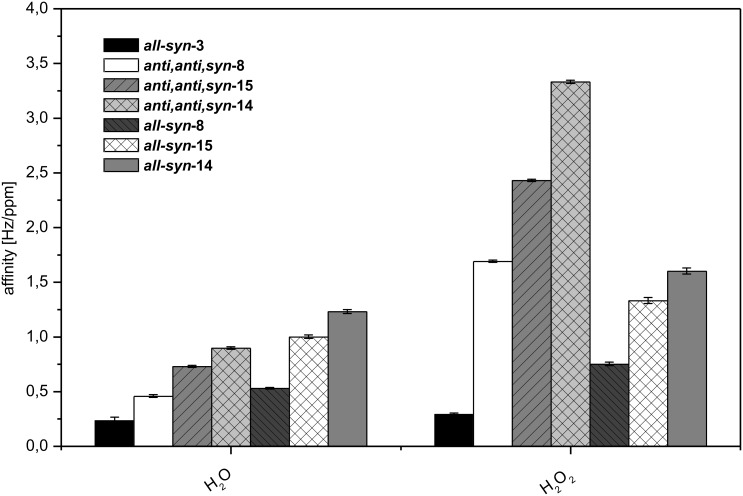
Screening of protic analytes with affinity materials **3**, **8**, **14** and **15**.

For protic analytes, *anti,anti,syn*-**8** is comparable or even inferior to other triptycene based materials (Figure 11). Therefore, *anti,anti,syn*-**8** is a very promising substance for the application as affinity material in a sensor array for the tracing of air-borne aromatic compounds at high dilution. Besides *anti,anti,syn*-**8**, the triphenylene based architecture all-*syn-***3** also exhibits excellent affinities towards the aromatic analytes as well as very inferior affinities to protic analytes (Figure 11).

The affinities of triptycene derivatives with different protecting groups on the carboxylic acid function to protic analytes were then compared (Figure 11). While the adsorption of aromatic analytes is dominated by the cavity size, the nature of the protection group dominates the adsorption of the protic analytes via hydrogen bonding [72]. A clear dependency on the number of nitro groups in the molecule, ascending from **8** with no nitro group to **14** with six nitro groups per molecule, is observed, indicating a strong influence of hydrogen bonding interactions between analyte and affinity material.

## Conclusion

Architectures based on triptycenes and (−)-isosteviol create molecular voids which can accommodate guest molecules. These molecular templates are the basis for powerful affinity materials of quartz crystal microbalances (QCM). In particular, the combination of 15-oxoisosteviol methyl ester with hexaaminotriptycene provides upon condensation reaction the statistically prone *anti,anti,syn*-derivative which shows unique properties as affinity material in QCM studies with aromatic analytes. Although this affinity material exhibits moderate performance at high analyte concentrations unusual strong signals are found at low concentrations of the analytes. This powerful and unique performance in the low ppm range is an essential prerequisite for latter tracing applications, e.g. gravimetric sensing. This superior property is attributed to well accessible molecular voids. As soon as such structures are allowed to collapse the outstanding affinity is not observed anymore.

## Experimental

For preparation and characterization of the compounds, see Supporting Information File 1.

## Supporting Information

File 1 Characterization data, spectra of synthesized compounds, QCM set up, and QCM screening details.
