# Supplementary material for: Novel supramolecular affinity materials based on (−)-isosteviol as molecular templates
Source: Beilstein J Org Chem. 2013 Dec 9;9:2821–33. doi: 10.3762/bjoc.9.317 (PMC3869372; doi:10.3762/bjoc.9.317)
Supplement: File 1 — Characterization data, spectra of synthesized compounds, QCM set up, and QCM screening details. [file Beilstein_J_Org_Chem-09-2821-s001.pdf]

Supporting Information

for

Novel supramolecular affinity materials based on (−)-isosteviol as molecular templates

Christina Lohoelter, Malte Brutschy, Daniel Lubczyk, and Siegfried R. Waldvogel*

Address: Institute for Organic Chemistry, Johannes Gutenberg University, Duesbergweg 10–14, 55128 Mainz, Germany

Email: Siegfried R. Waldvogel* - waldvogel@uni-mainz.de

*Corresponding author

Characterization data, spectra of synthesized compounds, QCM set up, and QCM screening details.

A. Materials and methods S2

B. Experimental procedures and characterization S3

C. ^{1}H and ^{13}C NMR spectra S33

D. Evaluation of affinity S57

E. References S65

A. Materials and methods

All reagents were used in analytical grades. Solvents were dried if necessary, by standard methods. Nitrogen which was used in the QMB screening experiments was used in a purity of 99.998%. Melting points were determined by a Melting Point apparatus B-545 (Büchi, Flawil, Switzerland) and were uncorrected. Microanalysis was performed with a VarioMICRO cube (Elementaranalysensysteme, Hanau, Germany). NMR spectra were recorded with a Bruker AC 300 or AV II 400 instrument (Bruker Analytische Messtechnik, Karlsruhe, Germany) by calibration on traces of $CHCl_3$ in the corresponding deuterated solvent with δ = 7.26 ppm for ^{1}H NMR and δ = 77.0 ppm for ^{13}C NMR, respectively; chemical shifts are expressed in ppm. The assignment of signals, if given, was determined via 2D NMR spectroscopy (COSY, HSQC, HMBC) or in accordance with literature [1]. Mass spectra and high resolution mass spectra were obtained on a QTof Ultima 3 apparatus (Waters, Milford, Massachusetts) employing ESI or on a MAT 95 (Thermo Finnigan, Bremen, Germany) employing FD. Optical rotations were measured using a JASCO P-2000 apparatus (Jasco Deutschland GmbH, Gross-Umstadt, Germany, path length 100 mm). All reactions were monitored by thin layer chromatography (TLC), visualization was effected by UV and heating with a 1% aqueous solution of $Ce(SO_4)_2{\cdot}4H_2O$ containing 2.5% of molybdato phosphoric acid and 6% of sulfuric acid. Column chromatography was performed on silica gel (particle size 63–200 µm, Merck, Darmstadt, Germany) or using a Büchi chromatography system (silica gel, particle size 40-63 µm, Macherey-Nagel GmbH, Düren, Germany) using mixtures of cyclohexane with ethyl acetate or dichloromethane with methanol as eluents. X-ray analysis data were collected on a STOE IPDS-2T diffractometer (Stoe, Darmstadt, Germany) using Incoatec microSource Cu Kα radiation (λ = 1.54186 Å). The structures were solved by direct methods and refined anisotropically by the least-

squares procedure implemented in the SHELX program system. CCDC-942549 (for *all-syn*-**17**) contains the supplementary crystallographic data for this paper. These data can be obtained free of charge from The Cambridge Crystallographic Data Centre via www.ccdc.cam.ac.uk/data_request/cif.

The experimental primary data of the QCM experiments were processed with Matlab 7.11.0 (R2010b) (The MathWorks Inc., Natick, Massachusetts, USA).

For the preparation of the diagrams OriginPro 8 SR0 (OriginLab Corporation, Northampton, Massachusetts, USA) were used.

HFF-QCMs with a fundamental frequency of 195 MHz were used (KVG Quartz Crystal Technology GmbH, Neckarbischofsheim, Germany. Type: XA 1600).

The QCM is excited using an aperiodic oscillator circuit and oscillates with its specific load resonance frequency [2]. Frequency counting is performed using a FPGA (field programmable gate array) which allows asynchronous 28-bit counting with an accuracy of ±0.5 Hz.

B. Experimental procedures and characterization

(+)-*all-syn*-Trispiro[tris-*ent*-beyerane-16,2´;16´,7´;16´´,12´-triphenyleno-[2,3-*d*;6,7-*d*´;10,11-*d*´´]tris[1,3]dioxole]-19,19´,19´´-nor-4,4´,4´´-tri-*N*-*p*-toluenesulfonamide (***all-syn*-3**)

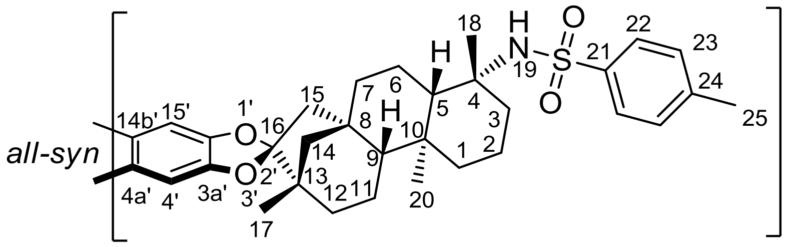

To a solution of all-*syn*-**2a** [1] (153 mg, 0.13 mmol) in pyridine (10 mL), *para*-toluenesulfonyl chloride (3.21 g, 16.8 mmol) was added at 25 °C. The reaction mixture was left to stand for seven days. After that, the solution was poured on ice

and left to stand for 45 minutes. 10% citric acid (10 mL) was added, the resulting orange precipitate was filtered off and washed with cold water (5 mL). The aqueous layer was extracted with dichloromethane (20 mL) and the organic fractions were washed with water (2 x 20 mL) and brine (20 mL), dried (Na_2SO_4) and concentrated under reduced pressure. The crude product was purified by column chromatography on silica (cyclohexane/ethyl acetate 9:1 to 75:25).

Yield: 68 mg (0.043 mmol, 32%) of a pale brown solid. R_f (CH:EE = 75:25): 0.39; Mp: decomposition >310 °C; ^{1}H-NMR (400 MHz, $CDCl_3$) δ [ppm] = 0.80 – 0.83 (m, 3H), 0.85 (s, 9H, H-20), 0.87 – 0.94 (m, 8H), 1.08 (s, 9H, H-17), 1.11 – 1.16 (m, 3H), 1.20 (s, 9H, H-18), 1.23 – 1.28 (m, 11H), 1.31 – 1.34 (m, 5H), 1.49 (dt, *J* = 4.0 Hz, 13.3 Hz, 3H), 1.63 – 1.66 (m, 9H), 1.70 – 1.72 (m, 3H), 1.79 (d, *J* = 11.9 Hz, 3H), 1.90 (d, *J* = 10.2 H, 3H), 2.01 (d, *J* = 15.4 Hz, 3H), 2.36 (d, $^2J_{15α,15β}$ = 13.9 Hz, 3H, H-$15_α$), 2.42 (s, 9H, H-25), 2.70 (d, $^2J_{3ax,3eq}$ = 15.1 Hz, 3H, H-3_{eq}), 4.30 (s, 3H, 19-N*H*), 7.27 (d, $^3J_{22,23}$ = 8.8 Hz, 6H, H-23), 7.65, 7.68 (every s, every 3H, H-4’, H-15’), 7.75 (d, $^3J_{22,23}$ = 8.3 Hz, 6H, H-22); ^{13}C-NMR (100 MHz, $CDCl_3$) δ [ppm] = 14.8 (C20), 17.1 (C2), 19.1 (C11), 19.4 (C17), 19.6 (C6), 21.5 (C25), 28.9 (C18), 34.8 (C12), 37.1 (C3), 37.5 (C10), 38.7 (C1), 40.6 (C7), 40.7 (C13), 46.8 (C8), 48.0 (C15), 54.2 (C14), 55.7, 56.9 (C5, C9), 58.7 (C4), 99.9, 100.0 (C4’, C15’), 124.1, 124.5 (C4a’, C14b’), 126.9 (C22), 127.8 (C16), 129.5 (C23), 140.5 (C24), 142.8 (C21), 147.4, 148.4 (C3a’, C15a’); MS (ESI pos. mode): m/z =1623.8 $[M+Na]^+$; HRMS (ESI, pos. mode): m/z for $C_{96}H_{117}NaN_3O_{12}S_3$ $[M+Na]^+$ calc.: 1622.7692, found: 1622.7686; elem. anal. $C_{96}H_{117}N_3O_{12}S_3$ (1601.16): calc. C 72.01 H 7.37 N 2.62 S 6.01, found: C 69.78 H 8.63 N 2.41 S 6.12; optical rotation $[α]_D^{20}$ = +55.9° (c 1.00, $CHCl_3$).

Deviations in the elemental analysis are probably caused by solvent molecules within the cavities of the molecules. The measurement of the optical rotation was therefore carried out to determine the orientation thereof.

Methyl (+)-*ent*-beyeran-19-oate-[15,16-*b*]quinoxaline (**6**)

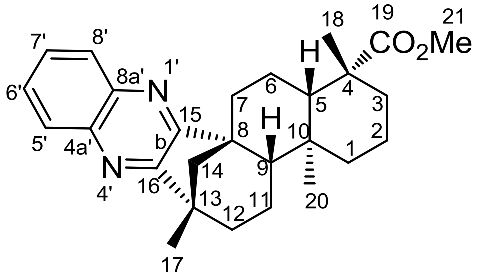

To a suspension of (+)-*ent*-15,16-dioxobeyeran-19-oic acid methyl ester **5b** [3] (100 mg, 0.29 mmol) in glacious acetic acid (10 mL), *o*-phenylenediamine (31 mg, 0.29 mmol) was added. The reaction mixture was refluxed for 4 hours. After bringing to room temperature, the mixture was fractionated between aqueous $NaHCO_3$ (50 mL) and *tert*-butyl methyl ether (TBME, 50 mL). The aqueous layer was extracted with TBME (2 x 20 mL), the combined organic fractions were washed with water (5 x 50 mL) and brine (50 mL), dried ($MgSO_4$), and concentrated under reduced pressure. The crude product was purified by column chromatography on silica (Büchi chromatography system, cyclohexane/ethyl acetate 98:2).

Yield: 50 mg (0.12 mmol, 41%) of a colorless solid. R_f (SiO_2, cyclohexane/ethyl acetate, 9:1) = 0.37; Mp. = 155 °C (ethyl acetate); ^{1}H-NMR (300 MHz, $CDCl_3$) δ [ppm] = 0.52 (s, 3H, H-20), 0.61 – 0.77 (m, 1H), 0.92 (dt, $^2J_{1ax,1eq}$ = $^3J_{1ax,2ax}$ = 13.1 Hz, $^3J_{1ax,2eq}$ = 4.1 Hz, 1H, H-1_{ax}), 1.05 (dt, $^2J_{3ax,3eq}$ = $^3J_{2ax,3ax}$ = 13.4 Hz, $^3J_{2eq,3ax}$ = 4.1 Hz, 1H, H-3_{ax}), 1.23 – 1.28 (m, 1H), 1.26 (s, 3H; H-17), 1.35 – 1.43 (m, 1H), 1.41 (s, 3H, H-18), 1.45 – 1.91 (m, 9H), 2.01 – 2.08 (m, 1H), 2.18 – 2.25 (m, 2H), 2.79 (dq,

$^{3}J_{6ax,7eq}$ = 3.4 Hz, $^{2}J_{6ax,6eq}$ = $^{3}J_{6ax,7ax}$ = $^{3}J_{6ax,5}$ = 13.7 Hz, 1H, H-6_{ax}), 3.72 (s, 3H, H-21), 7.60 – 7.66 (m, 2H, H-6‘, H-7‘), 8.00 – 8.06 (m, 2H, H-5‘, H-8‘); ^{13}C-NMR (75 MHz, $CDCl_3$) δ [ppm] = 11.7 (C20), 19.0 (C2), 21.2 (C6), 21.7 (C11), 22.1 (C17), 29.1 (C18), 36.7 (CH_2), 37.9 (CH_2), 38.4 (CH_2), 38.9 (C10), 40.5 (CH_2), 43.1, 44.1, 46.1 (C4, C8, C13), 51.3 (C21), 56.2, 57.1 (C5, C9), 58.8 (C14), 128.3, 128.6, 128.8, 129.6 (C5’ – C8’), 141.4, 141.9 (C4a’, C8a’), 165.7, 166.1 (C15, C16), 178.2 (C19); MS (ESI, pos. mode): m/z = 441.24 $[M+Na]^+$; 859.5 $[2M+Na]^+$; HRMS (ESI, pos. mode): m/z for $C_{27}H_{34}N_2NaO_2$ $[M+Na]^+$ calc.: 441.2518, found 441.2518; elem. anal. $C_{27}H_{34}N_2O_2$ (418.57): calc. C 77.48 H 8.19 N 6.69, found C 77.80 H 8.56 N 6.67; optical rotation $[\alpha]_D^{20}$ = +104.9° (c 1.00, $CHCl_3$).

Methyl (+)-*ent*-beyeran-19-oate-[15,16-*b*]-6´,7´-dimethylquinoxaline (**7**)

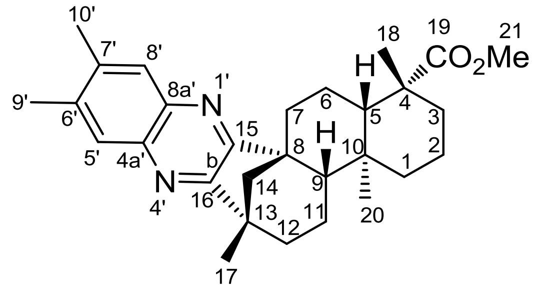

To a suspension of (+)-*ent*-15,16-dioxobeyeran-19-oic acid methyl ester **5b** [3] (500 mg, 1.45 mmol) in glacious acetic acid (50 mL), 4,5-dimethyl-1,2-phenylenediamine (195 mg, 1.45 mmol) was added. The reaction mixture was refluxed for 4 hours. After bringing to room temperature, the mixture was fractionated between aqueous $NaHCO_3$ (100 mL) and ethyl acetate (50 mL). The aqueous layer was extracted with EtOAc (2 x 50 mL), the combined organic fractions were washed with water (5 x 50 mL) and brine (50 mL), dried ($MgSO_4$), and concentrated under reduced pressure.

The crude product was purified by column chromatography on silica (Büchi chromatography system, cyclohexane/ethyl acetate 99:1).

Yield: 373 mg (0.87 mmol, 60%) of a colorless foam. R_f (SiO_2, cyclohexane/ethyl acetate, 4:1) = 0.63; ^{1}H-NMR (400 MHz, $CDCl_3$) δ [ppm] = 0.51 (s, 3H, H-20), 0.62 – 0.71 (m, 1H), 0.92 (dt, $^2J_{1ax,1eq}$ = $^3J_{1ax,2ax}$ = 13.3 Hz, $^3J_{1ax,2eq}$ = 4.2 Hz, 1H, H-1_{ax}), 1.05 (dt, $^2J_{3ax,3eq}$ = $^3J_{3ax,2ax}$ = 13.5 Hz, $^3J_{3ax,2eq}$ = 4.1 Hz, 1H, H-3_{ax}), 1.23 – 1.27 (m, 1H), 1.26 (s, 3H; H-17), 1.37 – 1.42 (m, 1H), 1.41 (s, 3H, H-18), 1.43 – 1.54 (m, 2H), 1.61 – 1.88 (m, 7H), 2.02 – 2.06 (m, 1H), 2.17 – 2.23 (m, 2H), 2.45, 2.46 (every s, every 3H, H-9‘, H-10‘), 2.77 (dq, $^3J_{6ax,7eq}$ = 3.5 Hz, $^2J_{6ax,6eq}$ = $^3J_{6ax,7ax}$ = $^3J_{6ax,5}$ =13.7 Hz, 1H, H-6_{ax}), 3.72 (s, 3H, H-21), 7.78, 7.83 (every s, every 1H, H-5‘, H-8‘); ^{13}C-NMR (100 MHz, $CDCl_3$) δ [ppm] = 11.8 (C20), 19.0 (CH_2), 20.06, 20.11 (C9’, C10’), 21.2 (CH_2), 21.7 (CH_2), 22.2 (C17), 28.9 (C18), 36.5 (CH_2), 37.6 (CH_2), 38.2 (CH_2), 38.6 (C), 40.3 (CH_2), 42.7 (C), 43.9 (C), 45.7 (C), 51.2 (C21), 55.9, 56.9 (C5, C9), 58.7 (C14), 128.7 (C5’, C8’), 138.1, 138.4 (C4a’, C8a’), 140.4 (C6’, C7’), 164.2, 164.8 (C15, C16), 178.1 (C19); MS (ESI, pos. mode): m/z = 447.32 $[M+H]^+$; HRMS (ESI, pos. mode): m/z for $C_{29}H_{39}N_2O_2$ $[M+H]^+$ calc. :447.3012, found 447.3003; elem. anal. $C_{29}H_{38}N_2O_2$ (446.62): calc. C 77.99 H 8.58 N 6.27, found C 77.90 H 9.06 N 6.16; optical rotation $[\alpha]_D^{20}$ = +140.0° (c 1.00, $CHCl_3$).

Methyl (+)-tris-*ent*-beyeran-19-oate-[16,15-*e*:15´,16´-*e*´:16´´,15´´-*e*´´]triptyceno*-[2*,3*-*b*:6*,7*-*b*´:14*,15*-*b*´´]tripyrazine (***all-syn*-8**)

Methyl (+)-tris-*ent*-beyeran-19-oate-[15,16-*e*:15´,16´-*e*´:16´´,15´´-*e*´´]triptyceno*-[2*,3*-*b*:6*,7*-*b*´:14*,15*-*b*´´]tripyrazine (***anti,anti,syn*-8**)

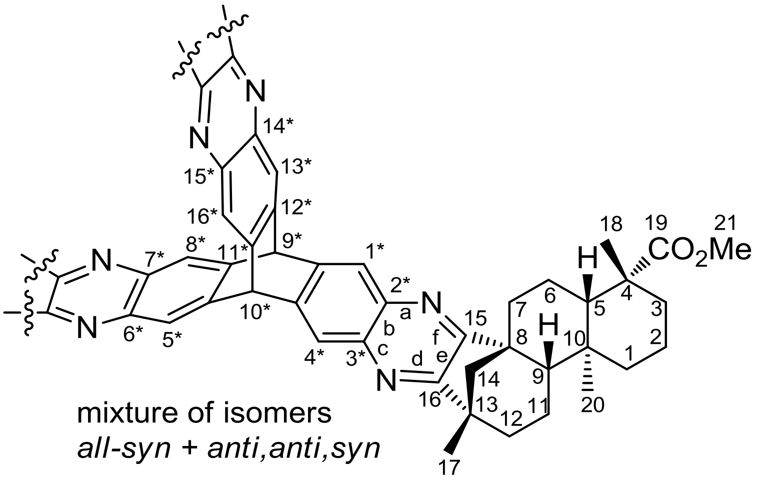

Hexaammoniumtriptycene hexachloride **4** [4] (54 mg, 0.07 mmol), (+)-*ent*-15,16-dioxobeyeran-19-oic acid methyl ester **5b** [3] (225 mg, 0.65 mmol), sodium acetate (71 mg, 0.87 mmol) and THF (2 mL) were placed in a sealed tube and heated to 100 °C for 16 h. After cooling to room temperature, the reaction mixture was fractionated between H_2O (50 mL) and CH_2Cl_2 (50 mL). The aqueous layer was extracted with CH_2Cl_2 (20 mL). The combined organic fractions were washed with H_2O (2 x 50 mL) and brine (50 mL), dried (Na_2SO_4), and concentrated under reduced pressure. The crude product was purified by column chromatography on silica (CH_2Cl_2/MeOH, 99.5:0.5 to 99:1).

Combined yield: 55 mg (0.042 mmol, 59% [all-*syn* + *anti,anti,syn*]) of an orange glassy solid.

Chromatographic separation of isomers:	*anti,anti,syn*	23 mg (25%)
	all-*syn*	3 mg (3%)

The remaining 31% were reisolated as isomeric mixture after column chromatography.

all-*syn*:

R_f (SiO_2, dichloromethane/methanol, 98:2) = 0.21; ^{1}H-NMR (600 MHz, $CDCl_3$) δ [ppm] = 0.42 (s, 9H, H-20), 0.52 – 0.59 (m, 3H), 0.85 – 0.90 (m, 5H), 1.03 (dt, $^{2}J_{3ax,3eq}$ = $^{3}J_{3ax,2ax}$ = 13.5 Hz, $^{3}J_{3ax,2eq}$ = 4.1 Hz, 3H, H-3_{ax}), 1.22 (d, *J* = 12.5 Hz, 3H), 1.26 (s, 9H, H-17), 1.38 (s, 9H, H-18), 1.41 – 1.49 (m, 7H), 1.56 (d, *J* = 12.6 Hz, 3H), 1.60 – 1.63 (m, 6H), 1.68 – 1.77 (m, 9H), 1.83 – 1.84 (m, 3H), 2.00 (d, *J* = 12.6 Hz, 3H), 2.10 (d, *J* = 13.2 Hz, 3H), 2.18 (d, *J* = 13.9 Hz, 3H), 2.73 (dq, $^{3}J_{6ax,7eq}$ = 3.5 Hz, $^{2}J_{6ax,6eq}$ = $^{3}J_{6ax,7ax}$ = $^{3}J_{6ax,5}$ =13.6 Hz, 3H, H-6_{ax}), 3.76 (s, 9H, H-21), 5.96, 5.99 (every s, every 1H, H-9*, H-10*), 8.11 – 8.19 (m, 6H, H-ar*); ^{13}C-NMR (150 MHz, $CDCl_3$) δ [ppm] = 11.6 (C20), 19.0 (C2), 21.1 (C6), 21.6 (C11), 22.1 (C17), 28.9 (C18), 29.7 (CH_2), 31.9 (C), 36.4 (CH_2), 37.4 (CH_2), 38.2 (CH_2), 38.6 (C), 40.2 (CH_2), 42.8 (C), 43.9 (C), 45.8 (C), 51.2 (C21), 52.9, 53.1, 55.8, 56.8 (C5, C9, C9*, C10*), 58.7 (C14), 123.3, 123.9 (C1*, C4*, C5*, C8*, C13*, C16*), 140.5 (C*), 143.0 (C*), 143.2 (C*), 165.0, 165.7 (C15, C16), 178.1 (C19); MS (MALDI-TOF, pos. mode): m/z = 1275.47 $[M+H]^+$; HRMS (ESI, pos. mode): m/z for $C_{83}H_{99}N_6O_6$ $[M+H]^+$ calc. 1275.7626, found 1275.7592; elem. anal. $C_{83}H_{98}N_6O_6$ (1275.70): calc. C 78.14 H 7.74 N 6.59, found C 75.42 H 8.33 N 5.48; optical rotation $[\alpha]_D^{20}$ = +64.7° (c 1.00, $CHCl_3$).

Deviations in the elemental analysis are probably caused by solvent molecules within the cavities of the molecules. The measurement of the optical rotation was therefore carried out to determine the orientation thereof.

anti,anti,syn

R_f (SiO_2, dichloromethane/methanol, 98:2) = 0.25; ^{1}H-NMR (600 MHz, $CDCl_3$) δ [ppm] = 0.40, 0.41 (every s, 9H, H-20), 0.47 – 0.55 (m, 3H), 0.85 – 1.05 (m, 9H), 1.18 – 1.22 (m, 5H), 1.25 (s, 9H; H-17), 1.34 – 1.37 (m, 12H), 1.40 – 1.49 (m, 6H), 1.53 – 1.63 (m, 9H), 1.65 – 1.73 (m, 6H), 1.79 – 1.80 (m, 5H), 1.98 – 2.19 (m, 9H), 2.68 –

2.77 (m, 3H, H-6_{ax}), 3.74, 3.75, 3.77 (every s, 9H, H-21), 5.94, 5.96 (every s, every 1H, H-9*, H-10*), 8.04 – 8.17 (m, 6H, H-ar*); ^{13}C-NMR (150 MHz, $CDCl_3$) δ [ppm] = 11.6, 11.7 (C20), 18.9 (CH_2), 19.0 (CH_2), 21.02 (CH_2), 21.05 (CH_2), 21.07 (CH_2), 21.54 (CH_2), 21.60 (CH_2), 21.62 (CH_2), 22.1 (C17), 28.87, 28.89 (C18), 36.35 (CH_2), 36.42 (CH_2), 37.39 (CH_2), 37.47 (CH_2), 37.49 (CH_2), 38.1 (CH_2), 38.5 (C), 40.2 (CH_2), 42.71 (C), 42.75 (C), 42.76 (C), 43.8 (C), 45.72 (C), 45.74 (C), 45.76 (C), 51.18, 51.20, 51.21 (C21), 52.9, 53.0, 55.7, 55.8, 56.80, 56.83 (C5, C9, C9*, C10*), 58.64, 58.66, 58.72 (C14), 123.2, 123.3, 123.8, 123.96, 124.0 (C1*, C4*, C5*, C8*, C13*, C16*), 140.2 (C*), 140.46 (C*), 140.48 (C*), 140.55 (C*), 142.8 (C*), 142.9 (C*), 143.0 (C*), 143.1 (C*), 143.3 (C*), 165.1, 165.4, 165.5, 165.6 (C15, C16), 178.00, 178.01 (C19); MS (MALDI-TOF, pos. mode): m/z = 1275.47 $[M+H]^+$; HRMS (ESI, pos. mode): m/z for $C_{83}H_{99}N_6O_6$ $[M+H]^+$ calc. 1275.7626, found 1275.7592; elem. anal. $C_{83}H_{98}N_6O_6$ (1275.70): calc. C 78.14 H 7.74 N 6.59, found C 75.49 H 9.27 N 5.69; optical rotation $[\alpha]_D^{20}$ = +87.2° (c 1.00, $CHCl_3$).

General remarks for the *anti,anti,syn*-isomers:

Due to isochronic effects caused by pseudo-symmetries within the *anti,anti,syn*-isomers, the number of signals in the corresponding ^{13}C NMR spectra does not necessarily correlate with the number of C-atoms in the molecules.

Deviations in the elemental analysis are probably caused by solvent molecules within the cavities of the molecules. The measurement of the optical rotation was therefore carried out to determine the orientation thereof.

3,5-Dinitrobenzyl-(−)-*ent*-16-oxobeyeran-19-oate (**12**)

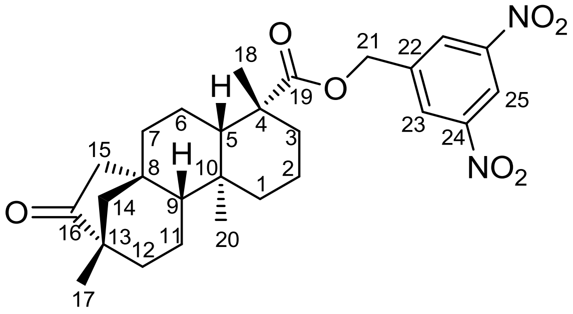

To a solution of (−)-isosteviol **1** (3.09 g, 9.7 mmol) in DMF (20 mL), triethylamine (2.3 mL, 17.1 mmol) and 3,5-dinitrobenzyl chloride (2.1 g, 9.7 mmol) were added at 25 °C. The solution was stirred at 25 °C for 16 h. After fractionation between 0.5 M HCl (50 mL) and EtOAc (150 mL), the aqueous layer was extracted with EtOAc (2 x 100 mL). The combined organic fractions were washed with aqueous $NaHCO_3$ (100 mL), H_2O (2 x 100 mL) and brine (100 mL), dried ($MgSO_4$), and concentrated under reduced pressure. The crude product was purified by column chromatography on silica (Büchi chromatography system, cyclohexane/ethyl acetate 99:1 to 96:4).

Yield: 2.94 g (5.9 mmol, 61%) of a colorless solid. R_f (SiO_2, cyclohexane/ethyl acetate, 4:1) = 0.38; Mp. 132 °C (ethyl acetate); ^{1}H NMR (300 MHz, $CDCl_3$) δ [ppm] = 0.66 (s, 3H, H-20), 0.84 – 0.94 (m, 1H, H-1_{ax}), 0.96 (s, 3H, H-17), 1.05 – 1.15 (m, 1H, H-3_{ax}), 1.16 – 1.23 (m, 3H, H-5, H-9, H-11_{ax}), 1.26 (s, 3H, H-18), 1.33 – 1.40 (m, 1H, H-12_{ax}), 1.42 – 1.50 (m, 2H, H-2_{eq}, H-14_{ax}), 1.53 – 1.58 (m, 2H, H-7_{ax}, H-14_{eq}), 1.61 – 1.83 (m, 7H, H-1_{eq}, H-2_{ax}, H-6_{ax}, H-7_{eq}, H-11_{eq}, H-12_{eq}, H-15_{β}), 1.94 – 1.98 (m, 1H, H-6_{eq}), 2.22 (d, $^2J_{3ax,3eq}$ = 13.3 Hz, 1H, H-3_{eq}), 2.56 (dd, $^2J_{15\alpha,15\beta}$ = 18.7 Hz, $^4J_{15\alpha,14}$ = 3.8 Hz, 1H, H-15_{α}), 5.19 – 5.36 (m, 2H, H-21), 8.55 (d, $^4J_{23,25}$ = 2.1 Hz, 2H, H-23), 9.00 (dd, $^4J_{23,25}$ = 2.1 Hz, 1H, H-25); ^{13}C NMR (75 MHz, $CDCl_3$) *δ* [ppm] = 13.5 (C20), 18.8 (C2), 19.8 (C17), 20.3 (C11), 21.9 (C6), 28.9 (C18), 37.2 (C12), 37.8 (C3), 38.0 (C10), 39.4 (C13), 39.5 (C1), 41.3 (C7), 44.0 (C4), 48.3 (C15), 48.6 (C8), 54.1 (C14),

54.6, 57.0 (C5, C9), 63.6 (C21), 118.3 (C25), 127.5 (C23), 140.8 (C22), 148.6 (C24), 176.7 (C19), 219.5 (C16); MS (ESI, pos. mode): m/z = 499.28 $[M+H]^+$; HRMS (ESI, pos. mode): m/z for $C_{27}H_{35}N_2O_7$ $[M+H]^+$ calc. 499.2444, found 499.2436; elem. anal. $C_{27}H_{34}N_2O_7$ (498.57): calc. C 65.04 H 6.87 N 5.62, found C 65.08 H 6.98 N 5.46; optical rotation $[\alpha]_D^{20}$ = –45.5° (c 1.00, $CHCl_3$).

3,5-Dinitrobenzyl-(−)-*ent*-15,16-dioxobeyeran-19-oate (**10**)

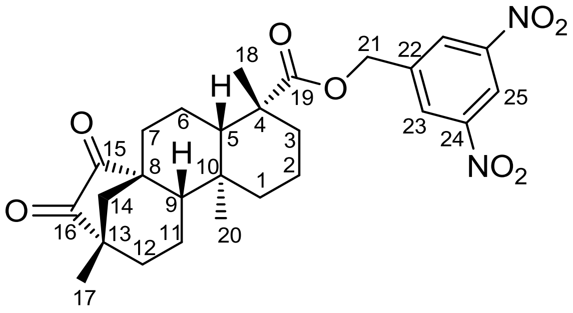

Procedure A

To a solution of (–)-*ent*-15,16-dioxobeyeran-19-oic acid (**9**, 100 mg, 0.3 mmol) in DMF (3 mL), triethylamine (0.07 mL, 0.52 mmol) and 3,5-dinitrobenzyl chloride (65 mg, 0.3 mmol) were added at 25 °C. The solution was stirred at 25 °C for 16 h. After fractionation between 0.5 M HCl (10 mL) and EtOAc (20 mL), the aqueous layer was extracted with EtOAc (2 x 10 mL). The combined organic fractions were washed with aqueous $NaHCO_3$ (10 mL), H_2O (2 x 50 mL) and brine (50 mL), dried ($MgSO_4$), and concentrated under reduced pressure. The crude product was purified by column chromatography on silica (Büchi chromatography system, cyclohexane/ethyl acetate 98:2 to 94:6) to give an orange foam (85 mg, 57%).

Procedure B

To a solution of 3,5-dinitrobenzyl-(−)-*ent*-16-oxobeyeran-19-oate **12** (2.9 g, 5.81 mmol) in *p*-xylene (100 mL), SeO_2 (0.95 g, 11.6 mmol) was added at 25 °C. The reaction mixture was heated to reflux for 2 d. After cooling to room temperature, the crude product was filtered by the aid of Celite™ and rinsed with dichloromethane (100 mL). The solvent was removed under reduced pressure and the product was purified by column chromatography on silica (Büchi chromatography system, cyclohexane/ethyl acetate 1:0 to 92:8).

Yield: 2.31 g (4.51 mmol, 78%) of an orange foam. R_f (SiO_2, cyclohexane/ethyl acetate, 4:1) = 0.32; ^{1}H NMR (300 MHz, $CDCl_3$) δ [ppm] = 0.47 (s, 3H, H-20), 0.84 – 1.10 (m, 3H), 1.12 (s, 3H, H-17), 1.18 – 1.26 (m, 3H), 1.29 (s, 3H, H-18), 1.44 – 1.55 (m, 2H), 1.58 – 1.65 (m, 2H), 1.73 – 1.82 (m, 3H), 1.90 – 2.03 (m, 3H), 2.19 – 2.38 (m, 2H, H-3_{eq}, H-6_{eq}), 5.26 – 5.37 (m, 2H, H-21), 8.60 (d, $^4J_{23,25}$.= 2.1 Hz, 2H, H-23), 9.02 (dd, $^4J_{23,25}$ = 2.1 Hz, 1H, H-25); ^{13}C NMR (75 MHz, $CDCl_3$) *δ* [ppm] = 11.5 (C20), 18.8 (C2), 20.1 (C17), 21.1 (C11), 21.4 (C6), 28.7 (C18), 33.9 (C7), 37.9 (C3), 38.8 (C10), 39.6, 39.7 (C1, C12), 44.0 (C4), 46.9 (C13), 47.1 (C14), 50.4 (C8), 56.1, 59.3 (C5, C9), 63.7 (C21), 118.8 (C25), 127.8 (C23), 140.6 (C22), 148.6 (C24), 176.5 (C19), 208.6, 210.1 (C15, C16); MS (ESI, pos. mode): m/z = 535.2 $[M+Na]^+$; HRMS (ESI, pos. mode):m/z for $C_{27}H_{32}NaN_2O_8$ $[M+Na]^+$ calc. 535.2056, found 535.2080; elem. anal. $C_{27}H_{32}N_2O_8$ (512.55): calc. C 63.27 H 6.29 N 5.47, found C 63.37 H 7.07 N 5.20; optical rotation $[\alpha]_D^{20}$ = –2.7° (c 1.00, $CHCl_3$).

4-Nitrobenzyl-(+)-*ent*-16-oxobeyeran-19-oate (**13**)

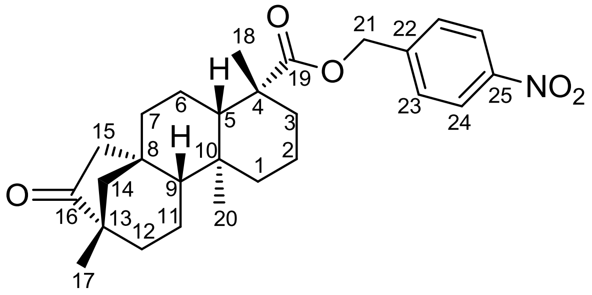

To a solution of (–)-isosteviol **1** (9.1 g, 28.2 mmol) in DMF (100 mL), cesium carbonate (13.8 g, 42.3 mmol) and 4-nitrobenzyl chloride (9.68 g, 56.4 mmol) were added at 25 °C under argon atmosphere. The solution was stirred at 25 °C for 5 h. After fractionation between H_2O (150 mL) and EtOAc (150 mL), the aqueous layer was extracted with EtOAc (2 x 150 mL). The combined organic fractions were washed with H_2O (2 x 150 mL) and brine (100 mL), dried ($MgSO_4$), and concentrated under reduced pressure. The crude product was purified by column chromatography on silica (cyclohexane/ethyl acetate 95:5 to 9:1).

Yield: 9.98 g (22 mmol, 77%) of a colorless solid. R_f (SiO_2, cyclohexane/ethyl acetate, 4:1) = 0.44; Mp. 118 °C (ethyl acetate); ^{1}H NMR (300 MHz, $CDCl_3$) δ [ppm] = 0.61 (s, 3H, H-20), 0.84 – 0.91 (m, 1H, H-1_{ax}), 0.92 (s, 3H, H-17), 1.03 (dt, $^2J_{3ax,3eq}$ = $^3J_{3ax,2ax}$ = 13.3 Hz, $^3J_{3ax,2eq}$ = 4.1 Hz, 1H, H-3_{ax}), 1.12 – 1.17 (m, 3H, H-5, H-9, H-11_{ax}), 1.20 (s, 3H, H-18), 1.29 – 1.45 (m, 4H), 1.47 – 1.49 (m, 1H), 1.52 – 1.54 (m, 1H), 1.65 – 1.68 (m, 4H), 1.71 – 1.79 (m, 2H), 1.88 (d, J = 14.1 Hz, 1H, H-6_{eq}), 2.17 (d, $^2J_{3ax,3eq}$ = 13.3 Hz, 1H, H-3_{eq}), 2.52 (dd, $^2J_{15α,15β}$ = 18.6 Hz, $^4J_{15α,14}$ = 3.7 Hz, 1H, H-$15_α$), 5.06 (d, $^2J_{21,21'}$ = 9.9 Hz, 1H, H-21), 5.21 (d, $^2J_{21,21'}$ = 9.9 Hz, 1H, H-21´), 7.49 (d, $^3J_{23,24}$ = 8.6 Hz, 2H, H-23), 8.18 (d, $^3J_{23,24}$ = 8.7 Hz, 2H, H-24); ^{13}C NMR (75 MHz, $CDCl_3$) δ [ppm] = 13.3 (C20), 18.8 (C2), 19.7 (C17), 20.2 (C11), 21.6 (C6), 28.8 (C18), 37.1 (C12), 37.7 (C3), 37.9 (C10), 39.3 (C13), 39.5 (C1), 41.2 (C7), 43.8 (C4),

48.2 (C15), 48.5 (C8), 54.1 (C14), 54.4, 56.9 (C5, C9), 64.5 (C21), 123.7 (C24), 128.4 (C23), 143.2 (C22), 147.5 (C25), 176.6 (C19), 219.3(C16); MS (ESI, pos. mode): m/z = 476.27 $[M+Na]^+$; HRMS (ESI, pos. mode): m/z for $C_{27}H_{35}NaNO_5$ $[M+Na]^+$ calc. 476.2413, found 476.2414; elem. anal. $C_{27}H_{35}NO_5$ (453.57): calc. C 71.50 H 7.78 N 3.09, found C 71.10 H 7.95 N 3.16; optical rotation $[\alpha]_D^{20}$ = +36.3° (c 1.00, $CHCl_3$).

4-Nitrobenzyl-(−)-*ent*-15,16-dioxobeyeran-19-oate (**11**)

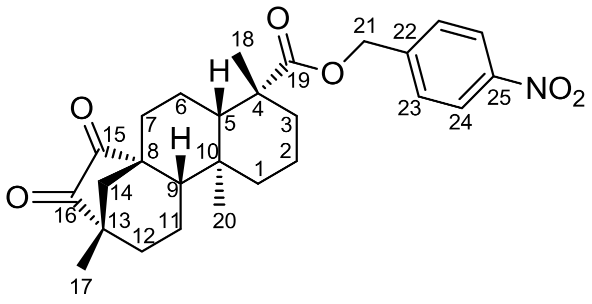

Procedure A

To a solution of (−)-*ent*-15,16-dioxobeyeran-19-oic acid (**9**, 911 mg, 2.73 mmol) in DMF (50 mL), cesium carbonate (1.34 g, 4.1 mmol) and 4-nitrobenzyl chloride (937 mg, 5.46 mmol) were added at 25 °C under argon atmosphere. The solution was stirred at 25 °C for 5 h. After fractionation between H_2O (100 mL) and EtOAc (150 mL), the aqueous layer was extracted with EtOAc (2 x 150 mL). The combined organic fractions were washed with H_2O (2 x 50 mL) and brine (50 mL), dried ($MgSO_4$), and concentrated under reduced pressure. The crude product was purified by column chromatography on silica (Büchi chromatography system, cyclohexane/ethyl acetate 98:2 to 96:4) to give an orange foam (863 mg, 68%).

Procedure B

To a solution of 4-nitrobenzyl-(+)-*ent*-16-oxobeyeran-19-oate (**13**, 8.97 g, 19.8 mmol) in *p*-xylene (150 mL), SeO_2 (3.24 g, 39.6 mmol) was added at 25 °C. The reaction mixture was heated to reflux for 2 d. After cooling to room temperature, the crude product was filtered by the aid of Celite™ and rinsed with dichloromethane (150 mL). The solvent was removed under reduced pressure and the product was purified by column chromatography on silica (Büchi chromatography system, cyclohexane/ethyl acetate 98:2 to 94:6).

Yield: 6.86 g (14.7 mmol, 74%) of an orange foam. R_f (SiO_2, cyclohexane/ethyl acetate, 4:1) = 0.33; ^{1}H NMR (300 MHz, $CDCl_3$) δ [ppm] = 0.53 (s, 3H, H-20), 0.90 (dt, $^2J_{1ax,1eq}$ = $^3J_{1ax,2ax}$ = 13.2 Hz, $^3J_{1ax,2eq}$ = 4.1 Hz, 1H, H-1_{ax}), 1.05 (dt, $^2J_{3ax,3eq}$ = $^3J_{3ax,2ax}$ = 13.5 Hz, $^3J_{3ax,2eq}$ = 4.1 Hz, 1H, H-3_{ax}), 1.13 (s, 3H, H-17), 1.19 – 1.23 (m, 2H, H-5, H-7_{ax}), 1.24 (s, 3H, H-18), 1.26 – 1.28 (m, 1H, H-11_{ax}), 1.42 – 1.47 (m, 1H, H-2_{eq}), 1.55 – 1.66 (m, 3H, H-1_{eq}, H-9, H-14_{ax}), 1.69 – 1.85 (m, 4H, H-2_{ax}, H-11_{eq}, H-12_{ax}, H-14_{eq}), 1.89 – 1.95 (m, 2H, H-6_{ax}, H-7_{eq}), 1.99 – 2.03 (m, 1H, H-12_{eq}), 2.21 (d, $^2J_{3ax,3eq}$ = 13.5 Hz, 1H, H-3_{eq}), 2.30 – 2.41 (m, 1H, H-6_{eq}), 5.12 – 5.21 (m, 2H, H-21), 7.59 (d, $^3J_{23,24}$ = 8.7 Hz, 2H, H-23), 8.25 (d, $^3J_{23,24}$ = 8.7 Hz, H-24); ^{13}C NMR (75 MHz, $CDCl_3$) *δ* [ppm] = 11.7 (C20), 18.8 (C2), 20.1 (C17), 20.9 (C11), 21.4 (C6), 28.7 (C18), 34.0 (C7), 38.0 (C3), 38.9 (C10), 39.7 (C1, C12), 43.9 (C4), 46.9 (C13), 47.2 (C14), 50.5 (C8), 56.2, 59.3 (C5, C9), 65.0 (C21), 123.8 (C24), 128.9 (C23), 143.1 (C22), 147.7 (C25), 176.7 (C19), 208.8, 210.2 (C15, C16); MS (ESI, pos. mode): m/z = 957.51 $[2M+Na]^+$; HRMS (ESI, pos. mode): m/z for $C_{27}H_{33}NaNO_6$ $[M+Na]^+$ calc. 490.2206, found 490.2190; elem. anal. $C_{27}H_{34}NO_6$ (467.55): calc. C 69.36 H 7.11 N 3.00, found C 68.77 H 7.25 N 3.11; optical rotation $[\alpha]_D^{20}$ = –12.5° (c 1.00, $CHCl_3$).

3,5-Dinitrobenzyl (+)-tris-*ent*-beyeran-19-oate-[16,15-*e*:15´,16´-*e*´:16´´,15´´-*e*´´]tripty-ceno*-[2*,3*-*b*:6*,7*-*b*´:14*,15*-*b*´´]tripyrazine (all-*syn*-**14**)

3,5-Dinitrobenzyl (+)-tris-*ent*-beyeran-19-oate-[15,16-*e*:15´,16´-*e*´:16´´,15´´-*e*´´]tripty-ceno*-[2*,3*-*b*:6*,7*-*b*´:14*,15*-*b*´´]tripyrazine (*anti,anti,syn*-**14**)

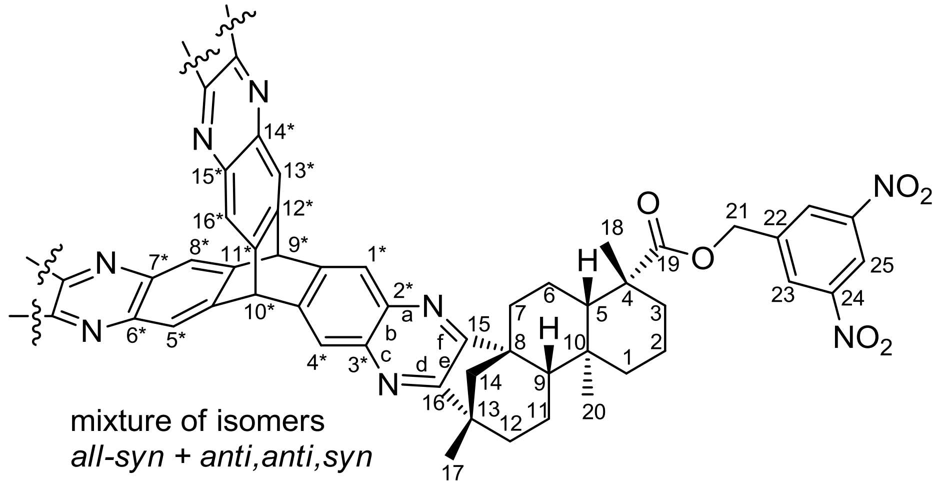

Hexaammoniumtriptycene hexachloride **4** [4] (344 mg, 0.42 mmol), 3,5-dinitrobenzyl-(–)-*ent*-15,16-dioxobeyeran-19-oate (**10**, 1.93 g, 3.76 mmol), sodium acetate (411 mg, 5.04 mmol) and THF (15 mL) were placed in a sealed tube and heated to 100 °C for 16 h. After cooling to room temperature, the reaction mixture was fractionated between H_2O (150 mL) and CH_2Cl_2 (150 mL). The aqueous layer was extracted with CH_2Cl_2 (100 mL). The combined organic fractions were washed with H_2O (3 x 50 mL) and brine (50 mL), dried (Na_2SO_4), and concentrated under reduced pressure. The crude product was purified by column chromatography on silica (**A**: Büchi chromatography system, cyclohexane/ethyl acetate 95:5 to 4:1 [removal of excess starting material]; **B**: CH_2Cl_2/MeOH, 99.5:0.5 [separation of isomers]).

Combined yield: 642 mg (0.36 mmol, 86% % [all-*syn* + *anti,anti,syn*]) of an orange glassy solid.

Chromatographic separation of isomers:	*anti,anti,syn*	361 mg (48%)
	all-*syn*	117 mg (17%)

The remaining 21% were reisolated as isomeric mixture after column chromatography.

all-*syn*

R_f (SiO_2, dichloromethane/methanol, 98:2) = 0.22; ^{1}H NMR (400 MHz, $CDCl_3$) δ [ppm] = 0.41 (s, 9H, H-20), 0.48 – 0.54 (m, 3H), 0.84 – 0.91 (m, 3H), 1.05 – 1.13 (m, 3H), 1.23 – 1.30 (m, 5H), 1.32, 1.34 (every s, every 9H, H-17, H-18), 1.36 – 1.48 (m, 9H), 1.52 – 1.60 (m, 9H), 1.65 – 1.76 (m, 10H), 2.04 – 2.11 (m, 6H), 2.20 – 2.23 (m, 3H), 2.72 – 2.81 (m, 3H, H-6_{ax}), 5.30 (d, $^2J_{21,21'}$= 13.7 Hz, 3H, H-21), 5.60 (d, $^2J_{21,21'}$= 13.7 Hz, 3H, H-21´), 5.85, 5.88 (ervery s, every 1H, H-9*, H-10*), 7.80, 8.07 (every s, every 3H, H-ar*), 8.76 (d, $^4J_{23,25}$ = 2.0 Hz, 6H, H-23), 8.98 (dd, $^4J_{23,25}$ = $^4J_{23',25}$ = 2.0 Hz, 3H, H-25); ^{13}C NMR (100 MHz, $CDCl_3$) δ [ppm] = 11.8 (C20), 18.8 (C2), 21.0 (C11), 21.8 (C6), 22.0 (C17), 28.7 (C18), 36.3 (CH_2), 37.3 (CH_2), 38.1 (CH_2), 38.6 (C), 39.9 (CH_2), 42.7 (C), 44.0 (C), 45.6 (C), 52.8, 53.0, 55.5, 56.7 (C5, C9, C9*, C10*), 58.5 (C14), 63.8 (C21), 118.2 (C25), 123.4, 123.6 (C1*, C4*, C5*, C8*, C13*, C16*), 128.1 (C23), 140.3, 140.4, 141.3, 142.9, 143.1 (C22, C*), 148.6 (C24), 165.1, 165.3 (C15, C16), 177.0 (C19); MS (MALDI-TOF, pos. mode): m/z = 1774.2 $[M+H]^+$ (Deviation from HRMS lies within the error margin of the instrument); HRMS (ESI, pos. mode): m/z for $C_{101}H_{105}N_{12}O_{18}$ $[M+H]^+$ calc. 1773.7670, found 1773.7678; elem. anal. $C_{101}H_{104}N_{12}O_{18}$ (1773.98): calc. C 68.38 H 5.91 N 9.47, found C 66.13 H 6.36 N 8.17; optical rotation $[\alpha]_D^{20}$ = +67.5° (c 1.00, $CHCl_3$).

Deviations in the elemental analysis are probably caused by solvent molecules within the cavities of the molecules. The measurement of the optical rotation was therefore carried out to determine the orientation thereof.

anti,anti,syn

R_f (SiO_2, dichloromethane/methanol, 98:2) = 0.32; ^{1}H NMR (400 MHz, $CDCl_3$) δ [ppm] = 0.40, 0.41 (every s, 9H, H-20), 0.45 – 0.55 (m, 3H), 0.83 – 0.94 (m, 3H), 1.07 – 1.15 (m, 3H), 1.24 – 1.31 (m, 5H), 1.33 – 1.38 (m, 16H), 1.41 – 1.52 (m, 9H), 1.58 – 1.63 (m, 9H), 1.69 – 1.73 (m, 6H), 1.76 – 1.81 (m, 6H), 2.03 – 2.15 (m, 6H), 2.18 – 2.24 (m, 3H), 2.67 – 2.84 (m, 3H, H-6_{ax}), 5.23 – 5.34 (m, 3H, H-21), 5.44 – 5.57 (m, 3H, H-21'), 5.91, 5.94 (every s, every 1H, H-9*, H-10*), 7.73 (s, 1H, H-ar*), 7.83 (s, 1H, H-ar*), 7.85 (s, 1H, H-ar*), 8.10 (s, 1H, H-ar*), 8.20 (s, 1H, H-ar*), 8.23 (s, 1H, H-ar*), 8.70, 8.71, 8.76 (every d, $^4J_{23,25}$ = 2.1 Hz, 6H, H-23), 9.02, 9.09, 9.11 (every dd, $^4J_{23,25}$ = $^4J_{23',25}$ = 2.1 Hz, 3H, H-25); ^{13}C NMR (100 MHz, $CDCl_3$) δ [ppm] = 11.8, 11.9, 12.0 (C20), 18.8 (CH_2), 18.9 (CH_2), 21.05 (CH_2), 21.08 (CH_2), 21.8 (CH_2), 21.9 (CH_2), 22.1 (C17), 28.8, 28.9, 29.0 (C18), 36.2 (CH_2), 36.3 (CH_2), 37.36 (CH_2), 37.41 (CH_2), 38.0 (CH_2), 38.1 (CH_2), 38.63 (C), 38.65 (C), 38.66 (C), 40.0 (CH_2), 42.74 (C), 42.78 C), 42.82 (C), 44.1 (C), 45.7 (C), 45.8 (C), 52.8, 52.9, 55.6, 55.7, 56.74, 56.78, 56.83 (C5, C9, C9*, C10*), 58.55, 58.62, 58.66 (C14), 63.8 (C21), 118.36, 118.37, 118.48 (C25), 123.4, 123.5, 123.6 (C1*, C4*, C5*, C8*, C13*, C16*), 127.9, 128.0, 128.3 (C23), 140.3, 140.40, 140.43, 140.5, 141.0; 141.1; 141.3; 142.9, 143.1, 143.3, 143.5 (C22, C*), 148.59, 148.67, 148.71 (C24), 165.0, 165.1, 165.2, 165.3, 165.5 (C15, C16), 176.88, 176.98, 177.02 (C21); MS (MALDI-TOF, pos. mode): m/z = 1774.2 $[M+H]^+$ (Deviation from HRMS lies within the error margin of the instrument); HRMS (ESI, pos. mode): m/z for $C_{101}H_{105}N_{12}O_{18}$ $[M+H]^+$ calc. 1773.7670, found 1773.7678; elem. anal. $C_{101}H_{104}N_{12}O_{18}$ (1773.98): calc. C 68.38 H 5.91 N 9.47, found C 67.80 H 6.00 N 9.17; optical rotation $[\alpha]_D^{20}$ = +106.9° (c 1.00, $CHCl_3$).

4-Nitrobenzyl (+)-tris-*ent*-beyeran-19-oate-[16,15-*e*:15´,16´-*e*´:16´´,15´´-*e*´´]triptyceno*-[2*,3*-*b*:6*,7*-*b*´:14*,15*-*b*´´]tripyrazine (all-*syn*-**15**)

4-Nitrobenzyl (+)-tris-*ent*-beyeran-19-oate-[15,16-*e*:15´,16´-*e*´:16´´,15´´-*e*´´]triptyceno*-[2*,3*-*b*:6*,7*-*b*´:14*,15*-*b*´´]tripyrazine (*anti,anti,syn*-**15**)

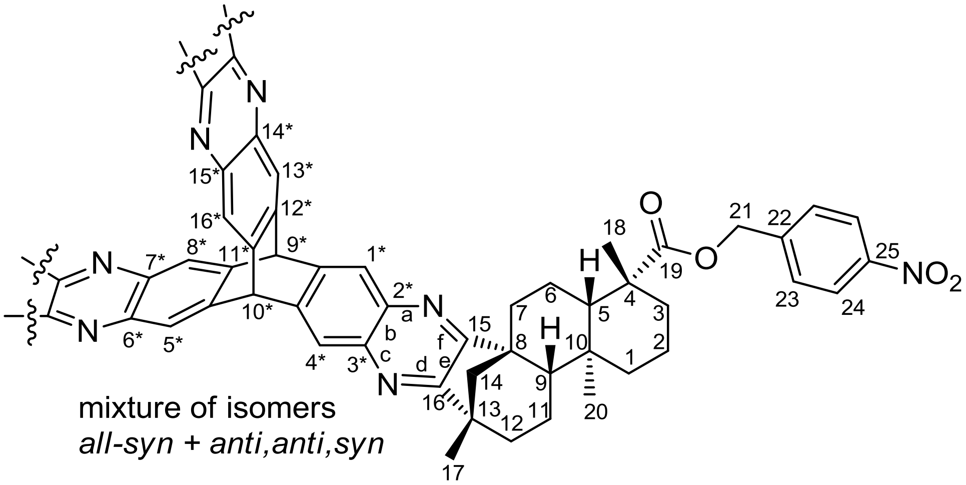

Hexaammoniumtriptycene hexachloride **4** [4] (1.34 g, 1.63 mmol), 4-nitrobenzyl-(−)-*ent*-15,16-dioxobeyeran-19-oate (**11**, 6.86 g, 14.7 mmol), sodium acetate (1.6 g, 19.6 mmol) and THF (10 mL) were placed in a sealed tube and heated to 100 °C for 16 h. After cooling to room temperature, the reaction mixture was fractionated between H_2O (150 mL) and CH_2Cl_2 (150 mL). The aqueous layer was extracted with CH_2Cl_2 (100 mL). The combined organic fractions were washed with H_2O (3 x 50 mL) and brine (50 mL), dried (Na_2SO_4), and concentrated under reduced pressure. The crude product was purified by column chromatography on silica (**A**: Büchi chromatography system, cyclohexane/ethyl acetate 98:2 to 88:12 [removal of excess starting material]; **B**: CH_2Cl_2/MeOH, 99.5:0.5 [separation of isomers]).

Combined yield: 2.47 g (1.55 mmol, 95% [all-*syn* + *anti,anti,syn*]) of an orange glassy solid.

Chromatographic separation of isomers:	*anti,anti,syn*	861 mg (34%)
	all-*syn*	303 mg (12%)

The remaining 49% were reisolated as isomeric mixture after column chromatography.

all-*syn*

R_f (SiO_2, dichloromethane/methanol, 98:2) = 0.23; ^{1}H NMR (600 MHz, $CDCl_3$) δ [ppm] = 0.45 (s, 9H, H-20), 0.49 – 0.58 (m, 3H), 0.90 (dt, $^2J_{1ax,1eq}$ = $^3J_{1ax,2ax}$ = 13.0 Hz, $^3J_{1ax,2eq}$ = 3.9 Hz, 3H, H-1_{ax}), 1.08 (dt, $^2J_{3ax,3eq}$ = $^3J_{3ax,2ax}$ = 13.5 Hz, $^3J_{3ax,2eq}$ = 3.9 Hz, 3H, H-3_{ax}), 1.27 – 1.29 (m, 3H), 1.31, 1.36 (every s, every 9H, H-17, H-18), 1.40 – 1.50 (m, 9H), 1.57 – 1.63 (m, 9H), 1.67 – 1.76 (m, 9H), 1.81 – 1.83 (m, 3H), 2.06 (dd, *J* = 2.8 Hz, 14.6 Hz, 3H), 2.13 (d, *J* = 13.2 Hz, 3H), 2.21 (d, $^2J_{3ax,3eq}$ = 13.5 Hz, 3H, H-3_{eq}), 2.80 (dq, $^3J_{6ax,7eq}$ = 3.5 Hz, $^2J_{6ax,6eq}$ = $^3J_{6ax,7ax}$ = $^3J_{6ax,5}$ =13.5 Hz, 3H, H-6_{ax}), 5.26 (d, $^2J_{21,21'}$ = 13.9 Hz, 3H, H-21), 5.36 (d, $^2J_{21,21'}$ = 13.8 Hz, 3H, H-21´), 5.80, 5.91 (every s, every 1H, H-9*, H-10*), 7.72 (d, $^3J_{23,24}$ = 8.7 Hz, 6H, H-23), 7.88, 8.13 (every s, every 3H, H-ar*), 8.30 (d, $^3J_{23,24}$ = 8.7 Hz, 6H, H-24); ^{13}C NMR (150 MHz, $CDCl_3$) δ [ppm] = 11.9 (C20), 18.9 (C2), 21.1 (C11), 21.8 (C6), 22.1 (C17), 28.9 (C18), 36.3 (CH_2), 37.4 (CH_2), 38.2 (CH_2), 38.7 (C), 40.1 (CH_2), 42.8 (C), 44.0 (C), 45.9 (C), 52.9, 53.0 (C9*, C10*), 53.4 (CH_2), 55.8, 56.8 (C5, C9), 58.7 (C14), 65.0 (C21), 123.7 (CH*, C24), 128.6 (C23), 140.5, 142.9, 143.2, 144.0 (C22, C*), 147.5 (C25), 164.9, 165.6 (C15, C16), 177.2 (C19); MS (MALDI-TO*F*, pos. mode): m/z = 1638.97 $[M]^+$; HRMS (ESI, pos. mode): m/z for $C_{101}H_{108}N_9O_{12}$ $[M+H]^+$ calc. 1638.8117, found 1638.8156; elem. anal. $C_{101}H_{107}N_9O_{12}$ (1638.98): calc. C 74.01 H 6.58, N 7.69, found C 73.38 H 7.68 N 7.27; optical rotation $[\alpha]_D^{20}$ = +126.6° (c 1.00, $CHCl_3$).

anti,anti,syn

R_f (SiO_2, dichloromethane/methanol, 98:2) = 0.33; ^{1}H NMR (600 MHz, $CDCl_3$) δ [ppm] = 0.35, 0.36, 0.44 (every s, every 3H, H-20), 0.50 – 0.60 (m, 3H), 0.82 – 0.92 (m, 5H), 1.04 – 1.10 (m, 3H), 1.24 – 1.29 (m, 5H), 1.30, 1.31, 1.32 (every s, every 3H, CH_3), 1.38 (s, 7H), 1.41 (s, 3H, CH_3), 1.44 – 1.50 (m, 5H), 1.55 – 1.64 (m, 10H), 1.67 – 1.73 (m, 5H), 1.79 – 1.83 (m, 5H), 2.06 – 2.23 (m, 9H), 2.78 – 2.85 (m, 3H, H-6_{ax}), 5.19 – 5.30 (m, 5 H, H-21), 5.41 – 5.43 (d, 1H, H-21), 5.84, 5.91 (every s, every 1H, H-9*, H-10*), 7.68 – 7.73 (m, 7H, H-ar), 7.87 (s, 1H, H-ar), 8.00 (s, 1H, H-ar), 8.16 (s, 1H, H-ar), 8.21 (s, 2H, H-ar), 8.28 – 8.31 (m, 4H, H-ar), 8.33 – 8.34 (d, 2H, H-ar); ^{13}C NMR (150 MHz, $CDCl_3$) δ [ppm] = 11.9, 12.0, 12.1 (C20), 18.86 (CH_2), 18.89 (CH_2), 18.91 (CH_2), 21.1 (CH_2), 21.7 (CH_2), 21.8 (CH_2), 22.1, 22.15, 22.18 (C17), 26.9 (CH_2), 28.81, 28.87, 28.93 (C18), 36.29 (CH_2), 36.33 (CH_2), 37.36 (CH_2), 37, 43 (CH_2), 38.18 (CH_2), 38.21 (CH_2), 38.66 (C), 38.67 (C), 40.1 (CH_2), 42.80 (C), 42.89 (C), 42.99 (C), 44.01 (C), 44.09 (C), 44.12 (C), 45.8 (C), 45.9 (C), 46.0 (C), 52.89, 52.92, 55.6, 55.7, 55.8, 56.76, 56.78, 56.80 (C5, C9, C9*, C10*), 58.6, 58.7 (C14), 64.9, 65.0, 65.1 (C21), 123.4 (CH), 123.6 (CH), 123.7 (CH), 123.76 (CH), 123.77 (CH), 123.8 (CH), 128.5, 128.68, 128.74 (C23), 140.54 (C), 140.56 (C), 142.6 (C), 142.9 (C), 143.6 (C), 143.7 (C), 144.1 (C), 147.49, 147.54, 147.6 (C25), 165.0, 165.5, 165.7, 165.8 (C15, C16), 177.1, 177.2, 177.3 (C19); MS (MALDI-TO*F*, pos. mode): m/z = 1638.97 $[M]^+$; HRMS (ESI, pos. mode): m/z for $C_{101}H_{108}N_9O_{12}$ $[M+H]^+$ calc. 1638.8117, found 1638.8156; elem. anal. $C_{101}H_{107}N_9O_{12}$ (1638.98): calc. C 74.01 H 6.58 N 7.69, found C 73.72 H 7.07 N 7.39; optical rotation $[\alpha]_D^{20}$ = +137.5° (c 1.00, $CHCl_3$).

(+)-Tris-*ent*-beyeran-19-oic acid-[16,15-*e*:15´,16´-*e*´:16´´,15´´-*e*´´]triptyceno*-[2*,3*-*b*:6*,7*-*b*´:14*,15*-*b*´´]tripyrazine (all-*syn*-**16**)

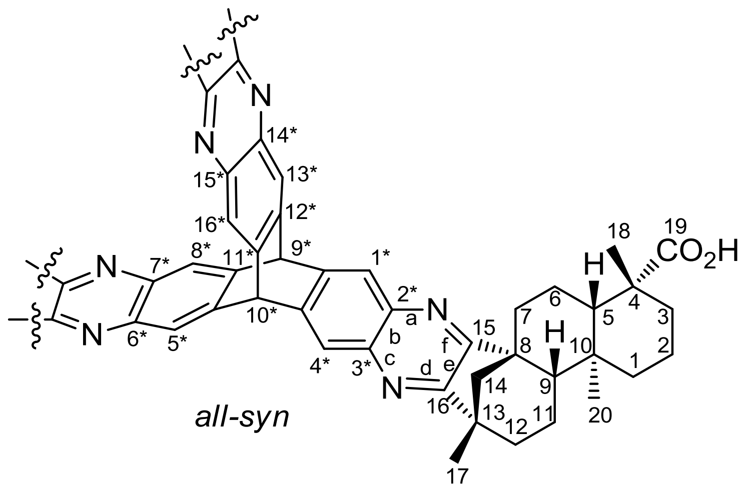

To a solution of all-*syn*-**15** (283 mg, 0.17 mmol) in THF (10 mL), palladium on activated charcoal (10% Pd on charcoal, 50% H_2O, 30 mg) was added under an argon atmosphere. The argon atmosphere was replaced by hydrogen atmosphere and the mixture was stirred at 25 °C for 3 d. The catalyst was then filtered off by the aid of Celite™. The filter cake was rinsed with CH_2Cl_2 (50 mL) and the filtrate was concentrated under reduced pressure. The crude product was purified by column chromatography on silica (Büchi chromatography system, cyclohexane/ethyl acetate 95:5 to 4:1).

Yield: 197 mg (0.16 mmol, 94%) of a colorless solid. R_f (SiO_2, dichloromethane/methanol, 95:5) = 0.35; ^{1}H NMR (400 MHz, THF-d8) δ [ppm] = 0.58 (s, 9H, H-20), 0.88 – 0.93 (m, 4H), 0.97 – 1.03 (m, 4H), 1.12 – 1.22 (m, 3H), 1.25 (s, 9H, H-17), 1.25 – 1.29 (m, 3H), 1.30 (s, 9H, H-18), 1.40 (s, 3H), 1.45 – 1.50 (m, 6H), 1.56 – 1.58 (m, 3H), 1.61 – 1.71 (m, 12H), 1.82 – 1.86 (m, 3H), 1.98 – 2.00 (m, 3H), 2.10 – 2.12 (m, 6H), 2.82 – 2.89 (m, 4H), 6.01, 6.13 (every s, every 1H, H-9*, H-10*), 8.06, 8.08 (every s, every 3H, H-ar*); ^{13}C NMR (100 MHz, THF-d8) δ [ppm] = 12.6

(C20), 20.2 (CH_2), 22.2 (CH_2), 22.6 (C17), 22.9 (CH_2), 29.7 (C18), 37.6 (CH_2), 38.5 (CH_2), 39.4 (CH_2), 39.8 (C), 41.4 (CH_2), 43.7 (C), 44.3 (C), 46.9 (C), 53.8, 54.1, 57.1, 57.8 (C5, C9, C9*, C10*), 60.0 (C14), 124.7 (CH-ar*), 141.6 (C*), 142.0 (C*), 144.5 (C*), 144.7 (C*), 165.9, 166.3 (C15, C16), 178.9 (C19) (The product is contaminated with about two equivalents of *p*-toluidine which is formed as side product by reductive cleavage of the *p*-nitrobenzyl protecting group); MS (MALDI-TOF, pos. mode): m/z = 1234.99 $[M+H]^+$ (Deviation from HRMS can be explained by the isotope distribution; 1:1-ratio of the two most intensive signals); HRMS (ESI, pos. mode): m/z for $C_{80}H_{93}N_6O_6$ $[M+H]^+$ calc. 1233.7157, found 1233.7184; elem. anal. $C_{80}H_{92}N_6O_6$ (1233.62): calc. C 77.89 H 7.52 N 6.81, found C 66.02 H 8.44 N 5.18; optical rotation $[\alpha]_D^{20}$ = +100.8° (c 1.00, $CHCl_3$).

Deviations in the elemental analysis are probably caused by solvent molecules within the cavities of the molecules. The measurement of the optical rotation was therefore carried out to determine the orientation thereof.

Pent-4-enyl (+)-tris-*ent*-beyeran-19-oate-[16,15-*e*:15´,16´-*e*´:16´´,15´´-*e*´´]triptyceno*-[2*,3*-*b*:6*,7*-*b*´:14*,15*-*b*´´]tripyrazine (all-*syn*-**17**)

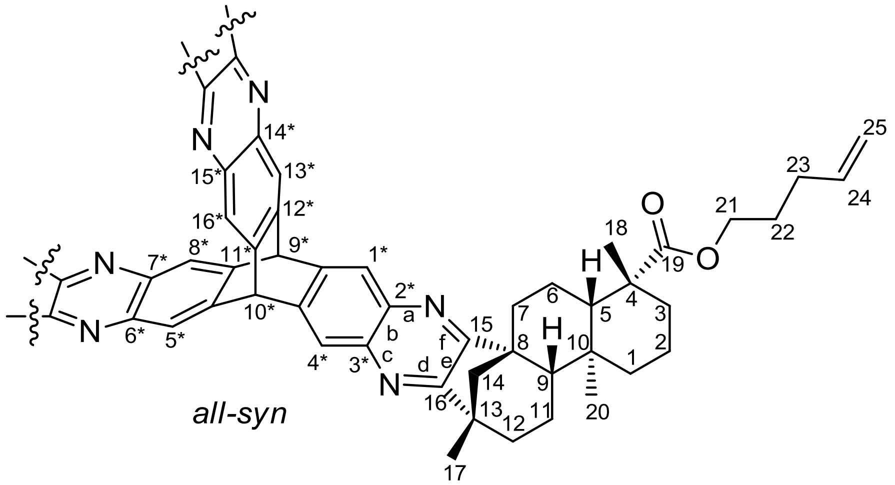

To a solution of all-*syn*-**16** (84 mg, 0.07 mmol) in DMF (5 mL), potassium carbonate (87 mg, 0.63 mmol) was added at 25 °C under argon atmosphere. The solution was stirred at 25 °C for 10 min. 5-Bromo-1-pentene (0.03 mL, 0.23 mmol) and TBAI (10 mg) were added, the reaction mixture was stirred at 25 °C for 3 h. After fractionation between H_2O (20 mL) and DCM (20 mL), the aqueous layer was extracted with DCM (2 x 20 mL). The combined organic fractions were washed with H_2O (2 x 50 mL) and brine (50 mL), dried (Na_2SO_4), and concentrated under reduced pressure. The crude product was purified by column chromatography on silica (Büchi chromatography system, cyclohexane/ethyl acetate 95:5 to 4:1).

Yield: 31 mg (0.022 mmol, 31%) of a pale yellow solid. R_f (SiO_2, dichloromethane/methanol, 98:2) = 0.22; ^{1}H NMR (400 MHz, $CDCl_3$) δ [ppm] = 0.44 (s, 9H, H-20), 0.49 – 0.60 (m, 3H), 0.86 (dt, $^2J_{1ax,1eq}$ = $^3J_{1ax,2ax}$ = 12.6 Hz, $^3J_{1ax,2eq}$ = 3.4 Hz, 3H, H-1$_{ax}$), 1.00 (dt, $^2J_{3ax,3eq}$ = $^3J_{3ax,2ax}$ = 13.3 Hz, $^3J_{3ax,2eq}$ = 3.9 Hz, 3H, H-3$_{ax}$), 1.20 – 1-23 (m, 3H), 1.26 (s, 9H, H-17), 1.33 (s, 9H, H-18), 1.35 – 1.46 (m, 9H), 1.53 – 1.73 (m, 19H), 1.79 – 1.82 (m, 3H), 1.92 – 2.05 (m, 9H), 2.09 – 2.20 (m, 6H), 2.38 – 2.44 (m, 6H), 2.78 (dq, $^3J_{6ax,7eq}$ = 3.0 Hz, $^2J_{6ax,6eq}$ = $^3J_{6ax,7ax}$ = $^3J_{6ax,5}$ = 13.1 Hz, 3H, H-6$_{ax}$), 4.08 (dt, $^2J_{21,21'}$ = 11.0 Hz, $^3J_{21,22}$ = 6.6 Hz, 3H, H-21), 4.20 (dt, $^2J_{21,21'}$ = 11.0 Hz, $^3J_{21,22}$ = 6.4 Hz, 3H, H-21´), 5.19 – 5.30 (m, 6H, H-25), 5.86, 5.88 (every s, every 1H, H-9*, H-10*), 6.05 (ddt, *J* = 6.7 Hz, 10.1 Hz, 16.9 Hz, 3H, H-24), 8.04, 8.08 (every s, every 3H, H-ar*); ^{13}C NMR (100 MHz, $CDCl_3$) δ [ppm] = 11.9 (C20), 19.0 (C2), 21.1 (C6), 21.7 (C11), 22.1 (C17), 28.0 (C22), 29.0 (C18), 30.7 (C23), 36.5 (CH_2), 37.4 (CH_2), 38.3 (CH_2), 38.7 (C), 40.3 (CH_2), 42.7 (C), 43.9 (C), 45.8 (C), 52.9, 53.1, 55.8, 56.9 (C5, C9, C9*, C10*), 58.8 (C14), 63.7 (C21), 115.8 (C25), 123.4, 123.8 (CH*), 137.9 (C24), 140.3, 140.5, 142.9, 143.1 (C*), 165.2, 165.5 (C15, C16), 177.7 (C19); MS (MALDI-TOF, pos. mode): m/z = 1440.55 $[M+H]^+$ (Deviation from HRMS can be

explained by the isotope distribution; 1:1-ratio of the two most intensive signals); HRMS (ESI, pos. mode): m/z for $C_{95}H_{117}N_6O_6$ $[M+H]^+$ calc. 1437.9035, found 1437.9037; elem. anal. $C_{95}H_{116}N_6O_6$ (1437.97): calc. C 79.35 H 8.13 N 5.84, found C 79.00 H 9.89 N 5.60; optical rotation $[\alpha]_D^{20}$ = +86.1° (c 1.00, $CHCl_3$).

Crystal structure determination:

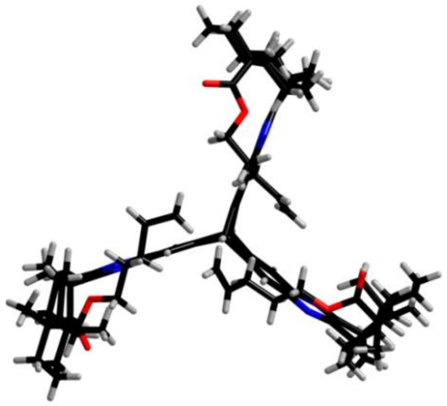

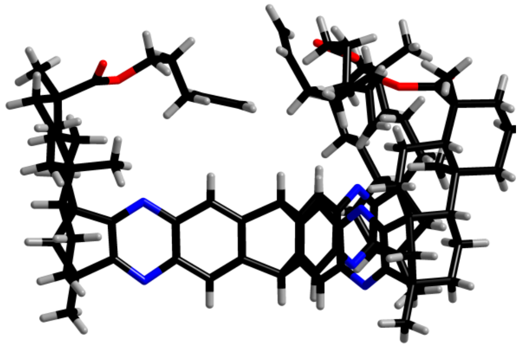

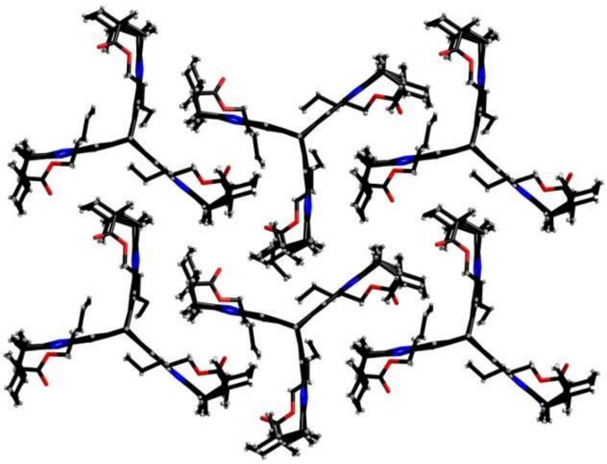

A suitable single crystal for X-ray analysis of all-*syn*-**17** was obtained by diffusion of *n*-heptane into a solution of **17** in CH_2Cl_2/MeOH at ambient conditions to give colorless needles.

Formula $C_{95}H_{116}N_6O_6$, M = 1437.94, 0.02 x 0.13 x 0.35 mm^3, orthorhombic, space group $P2_12_12_1$, a = 14.1535(6), b = 23.4332(16), c = 24.6827(11) Å, α = 90°, β = 90°, γ = 90°, V = 8186.3(8) Å^3, ρ_{calc} = 1.167 gcm^{-3}, μ = 0.56 mm^{-1}, Z = 4, λ= 1.54178 Å, T = 193(2) K, F(000) = 3104, 111207 reflections collected, 14263 independent (R_{int} = 0.2386), 14263 observed, 974 refined parameters, GOF = 1.157, R^1 = 0.1344 for

observed and 0.2440 for all reflections, wR^2 = 0.4470, largest difference peak and hole: 0.53 and −0.48 eÅ^{-3}.

The size of the cavity which is formed ranges approximately to the quinoxaline-nitrogen atoms of the triptycene core which exhibit a mutual distance of 8.1 Å, 8.8 Å and 8.9 Å, whereas the carboxylic carbon atoms exhibit a distance of about 12 Å. The cavity of all-*syn*-**17** is “roofed” by the alkyl chains of the (−)-isosteviol units, whose terminal carbon atoms show a mutual distance in the range of 4.2–5.2 Å. Within a plane, the voids which are created by the aromatic faces of the triptycene are filled by (−)-isosteviol fragments of a neighbouring molecule.

Pent-4-enyl-(−)-*ent*-15,16-dioxobeyeran-19-oate (**18**)

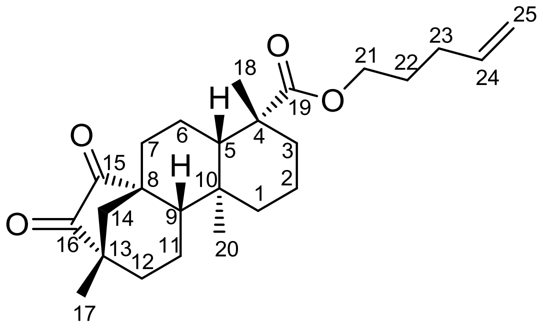

To a solution of (−)-*ent*-15,16-dioxobeyeran-19-oic acid (**9**, 7.07 g, 21.3 mmol) in DMF (200 mL), potassium carbonate (8.82 g, 63.9 mmol) was added at 25 °C under argon atmosphere. The solution was stirred at 25 °C for 10 min. 5-Bromo-1-pentene (3.03 mL, 23.4 mmol) and TBAI (20 mg) were added, the reaction mixture was stirred at 25 °C for 4 h. After fractionation between H_2O (20 mL) and EtOAc (200 mL), the aqueous layer was extracted with EtOAc (2 x 150 mL). The combined organic fractions were washed with H_2O (2 x 150 mL) and brine (100 mL), dried ($MgSO_4$), and concentrated under reduced pressure. The crude product was purified by column chromatography on silica (cyclohexane/ethyl acetate 96:4).

Yield: 7.3 g (18.2 mmol, 85%) of an orange solid. R_f (SiO_2, cyclohexane/ethyl acetate, 4:1) = 0.5; Mp. = 100 °C (ethyl acetate); ^{1}H NMR (300 MHz, $CDCl_3$) δ [ppm] = 0.55 (s, 3H, H-20), 0.86 (dt, $^2J_{1ax,1eq}$ = $^3J_{1ax,2ax}$ = 13.1 Hz, $^3J_{1ax,2eq}$ = 4.2 Hz, 1H, H-1_{ax}), 0.98 (dt, $^2J_{3ax,3eq}$ = $^3J_{3ax,2ax}$ = 13.5 Hz, $^3J_{3ax,2eq}$ = 4.1 Hz, 1H, H-3_{ax}), 1.11 (s, 3H, H-17), 1.17 – 1.18 (m, 1H, H-5), 1.19 (s, 3H, H-18), 1.21 – 1.28 (m, 2H, H-7_{ax}, H-11_{ax}), 1.36 – 1.44 (m, 1H, H-2_{eq}), 1.52 – 1.65 (m, 3H), 1.70 – 1.84 (m, 6H), 1.88 – 1.90 (m, 1H), 1.93 – 2.01 (m, 2H), 2.13 – 2.21 (m, 3H), 2.28 – 2.42 (m, 1H), 4.03 (t, $^3J_{21,22}$ = 6.6 Hz, 2H, H-21), 4.95 – 5.09 (m, 2H, H-25), 5.81 (ddt, J = 6.7 Hz, 10.2 Hz, 16.9 Hz, 1H, H-24); ^{13}C NMR (75 MHz, $CDCl_3$) δ [ppm] = 11.6 (C20), 18.9 (C2), 20.1 (C17), 20.8 (C6), 21.4 (C11), 27.6 (C22), 28.8 (C18), 30.2 (C23), 34.1 (C7), 37.9 (C3), 38.8 (C10), 39.7, 39.8 (C1, C12), 43.7 (C4), 46.9 (C13), 47.2 (C14), 50.5 (C8), 56.2, 59.4 (C5, C9), 63.7 (C21), 115.3 (C25), 137.4 (C24), 177.1 (C19), 208.9, 210.0 (C15, C16); MS (FD): m/z = 400.6 $[M]^{+\bullet}$ (Deviation from HRMS lies within the error margin of the instrument); HRMS (ESI, pos. mode): m/z for $C_{25}H_{36}NaO_4$ $[M+Na]^+$ calc. 423.2511, found 423.2514; elem. anal. $C_{25}H_{36}O_4$ (400.55): calc. C 74.96 H 9.06, found C 74.78 H 9.03; optical rotation $[\alpha]_D^{20}$ = –116.7° (c 1.00, $CHCl_3$).

Pent-4-enyl (+)-tris-*ent*-beyeran-19-oate-[16,15-*e*:15´,16´-*e*´:16´´,15´´-*e*´´]triptyceno*-[2*,3*-*b*:6*,7*-*b*´:14*,15*-*b*´´]tripyrazine (all-*syn*-**17**)

Pent-4-enyl (+)-tris-*ent*-beyeran-19-oate-[15,16-*e*:15´,16´-*e*´:16´´,15´´-*e*´´]triptyceno*-[2*,3*-*b*:6*,7*-*b*´:14*,15*-*b*´´]tripyrazine (*anti,anti,syn*-**17**)

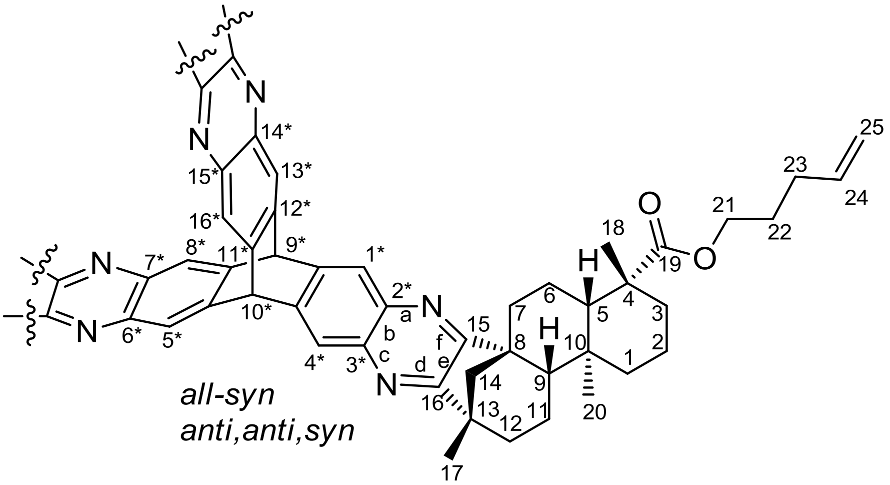

Hexaammoniumtriptycene hexachloride **4** [4] (1.17 g, 1.42 mmol), pent-4-enyl-(–)-*ent*-15,16-dioxobeyeran-19-oate (**18**, 5.12 g, 12.9 mmol), sodium acetate (1.39 g, 17.0 mmol) and THF (20 mL) were placed in a sealed tube and heated to 100 °C for 16 h. After cooling to room temperature, the reaction mixture was fractionated between H_2O (150 mL) and CH_2Cl_2 (150 mL). The aqueous layer was extracted with CH_2Cl_2 (100 mL). The combined organic fractions were washed with H_2O (3 x 50 mL) and brine (50 mL), dried (Na_2SO_4), and concentrated under reduced pressure. The crude product was purified by column chromatography on silica (**A**: Büchi chromatography system, cyclohexane/ethyl acetate 98:2 to 4:1 [removal of excess starting material]; **B**: CH_2Cl_2/MeOH, 99.5:0.5 to 99:1 [separation of isomers]). Combined yield: 1.34 g (0.96 mmol, 67% [all-*syn* + *anti,anti,syn*]) of a pale yellow solid.

Chromatographic separation of isomers:	*anti,anti,syn*	879 mg (44%)
	all-*syn*	461 mg (23%)

all-*syn:* see above

anti,anti,syn

R_f (SiO_2, dichloromethane/methanol, 98:2) = 0.24; ^{1}H NMR (400 MHz, $CDCl_3$) δ [ppm] = 0.39, 0.43 (every s, 9H, H-20), 0.46 – 0.56 (m, 3H), 0.79 – 0.87 (m, 3H), 0.93 – 1.04 (m, 3H), 1.14 – 1.22 (m, 3H), 1.25, 1.26 (every s, 9H, H-17), 1.32, 1.35 (every s, 9H, H-18), 1.32 – 1.48 (m, 9H), 1.53 – 1.59 (m, 9H), 1.62 – 1.71 (m, 6H), 1.74 – 1.76 (m, 6H), 1.91 – 2.05 (m, 9H), 2.09 – 2.20 (m, 6H), 2.37 – 2.45 (m, 6H), 2.73 – 2.79 (m, 3H, H-6_{ax}), 4.03 – 4.09 (m, 3H, H-21), 4.17 – 4.23 (m, 3H, H-21'), 5.23 – 5.32 (m, 6H, H-25), 5.86, 5.87 (every s, every 1H, H-9*, H-10*), 6.02 – 6.13 (m, 3H, H-24), 8.01 (s, 1H, H-ar*), 8.05 (s, 1H, H-ar*), 8.06 (s, 1H, H-ar*), 8.07 (s, 1H, H-ar*), 8.11 (s, 2H, H-ar*); ^{13}C NMR (100 MHz, $CDCl_3$) δ [ppm] = 11.9, 12.0 (C20), 18.90 (CH_2), 18.94 (CH_2), 21.1 (CH_2), 21.6 (CH_2), 21.7 (CH_2), 22.1 (C17), 27.9 (CH_2), 28.00 (CH_2), 28.04 (CH_2), 28.9 (C18), 30.60 (CH_2), 30.68 (CH_2), 30.73 (CH_2), 36.4 (CH_2), 37.4 (CH_2), 37.5 (CH_2), 38.2 (CH_2), 38.3 (CH_2), 38.62 (C), 38.63 (C), 38.65 (C), 40.2 (CH_2), 40.3 (CH_2), 42.68 (C), 42.71 (C), 43.88 (C), 43.91 (C), 45.8 (C), 52.9, 53.0, 55.75, 55.77, 56.8 (C5, C9, C9*, C10*), 58.7, 58.8 (C14), 63.70, 63.75, 63.83 (C21), 115.80, 115.83, 115.86 (C24), 123.2, 123.3, 123.8, 123.99, 124.00 (CH*), 137.88, 137.91, 137.98 (C24), 140.3, 140.46, 140.51, 142.7, 142.9, 143.0, 143.08, 143.10 (C*), 165.09, 165.16, 165.19, 165.4, 165.5, 165.6 (C15, C16), 177.6, 177.7 (C19); MS (MALDI-TOF, pos. mode): m/z = 1440.55 $[M+H]^+$ (Deviation from HRMS can be explained by the isotope distribution; 1:1-ratio of the two most intensive signals); HRMS (ESI, pos. mode): m/z for $C_{95}H_{117}N_6O_6$ $[M+H]^+$ calc. 1437.9035, found 1437.9037; elem. anal. $C_{95}H_{116}N_6O_6$ (1437.97): calc. C 79.35 H 8.13 N 5.84, found C 79.34 H 9.18 N 6.03; optical rotation $[\alpha]_D^{20}$ = +120.9° (c 1.00, $CHCl_3$).

Cage structure (**19**)

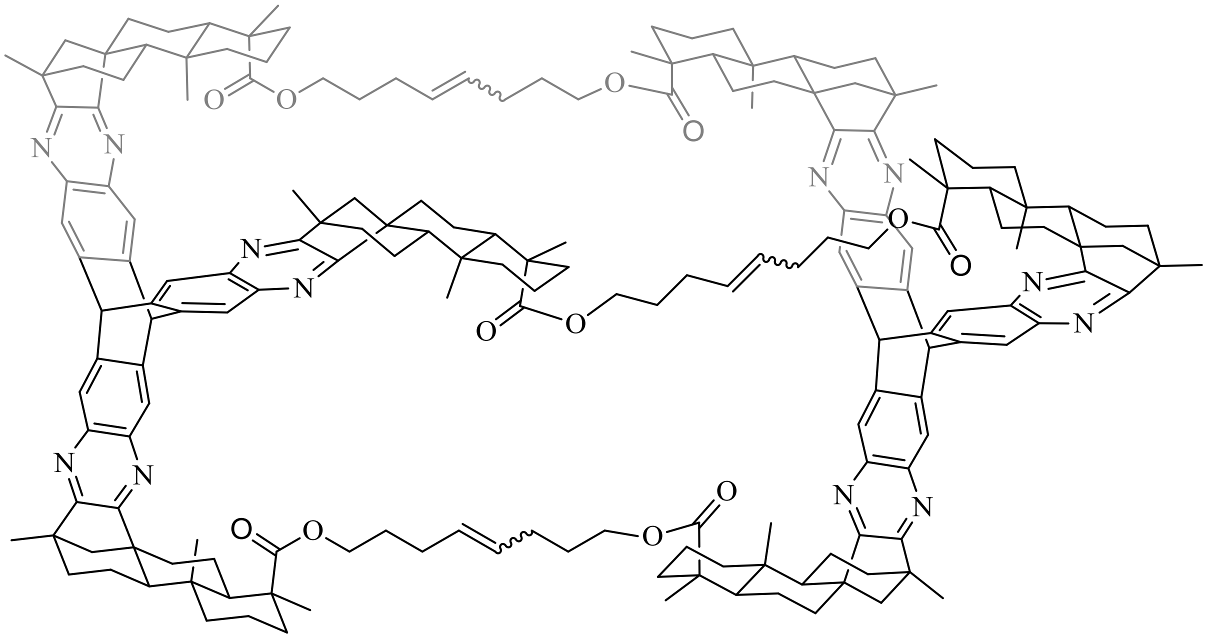

Under argon atmosphere, all-*syn*-**17** (50 mg, 0.036 mmol) and Grubbs catalyst (2nd generation, 12 mg, 0.012 mmol) were solved in degassed dichloromethane (100 mL each) in separated flasks. The solution of all-*syn*-**17** was then added to the catalyst solution and the resulting mixture was refluxed for 34 days. After removal of the solvent under reduced pressure, the crude product was purified by column chromatography on silica (dichloromethane/methanol, 99.5:0.5 to 98:2) to yield a brown solid.

R_f (dichloromethane/methanol, 98:2) = 0.20; ^{13}C NMR (150 MHz, $CDCl_3$) δ [ppm] = 11.6, 12.0, 12.3, 13.9 (every CH_3), 18.8, 19.2, 21.0, 21.1, 21.4, 21.7 (every CH_2), 21.9, 22.1, 22.2 (every CH_3), 28.48, 28.57 (every CH_2), 28.61, 28.9, 29.2 (every CH_3), 29.3, 29.7, 30.2, 30.6, 30.9, 31.6 (every CH_2), 31.9 (C), 34.9, 35.3 (every CH_3), 36.4, 36.5, 36.6, 37.4, 37.6, 38.5 (every CH_2), 38.8 (C), 40.0, 40.3 (every CH_2), 42.9, 43.7, 43.9, 44.2, 45.6, 45.8 (every C), 50.8, 53.0, 53.4, 55.1, 55.3, 55.6, 56.6, 56.7, 56.8 (every CH), 58.3, 58.5, 58.7, 61.8, 63.2, 63.3, 64.1, 64.3, 65.2, 70.2, 70.5, 71.3,

72.5 (every CH_2), 123.3, 123.6, 124.2, 129.6, 130.3, 131.5 (every CH), 140.4, 140.6, 142.2, 143.4, 165.1, 165.4, 177.0, 178.1 (every C); MS (MALDI-TOF, pos. mode): m/z = 2793.59 $[M+H]^+$.

Note: Complete conversion of the starting material could not be achieved. The brown color of the product as well corresponding signals in the NMR spectra indicate the presence of catalyst fragments within the product. Due to a very small amount of product with a comparatively high molecular weight, a complete characterization by NMR methods has not been achieved yet. For discussion on this topic, see publication.

C. ^{1}H and ^{13}C NMR spectra

Compound all-*syn*-**3**:

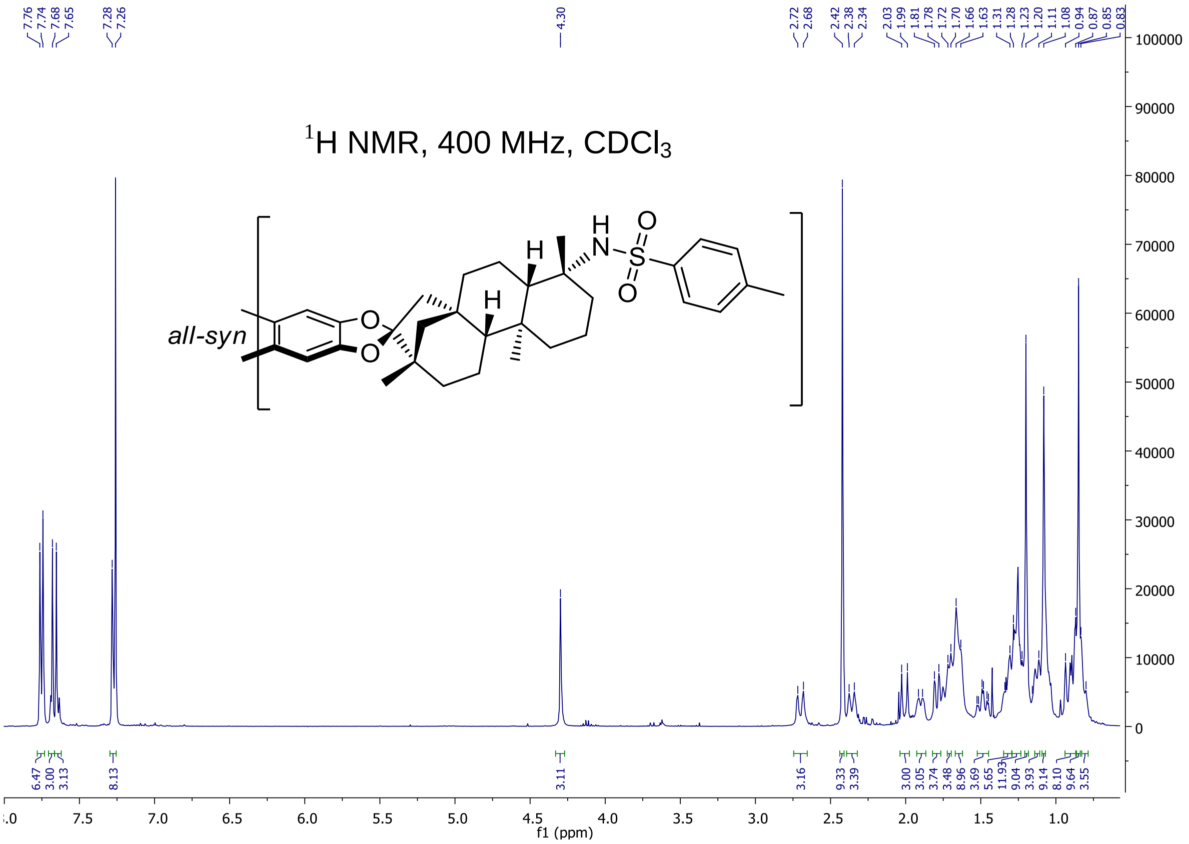

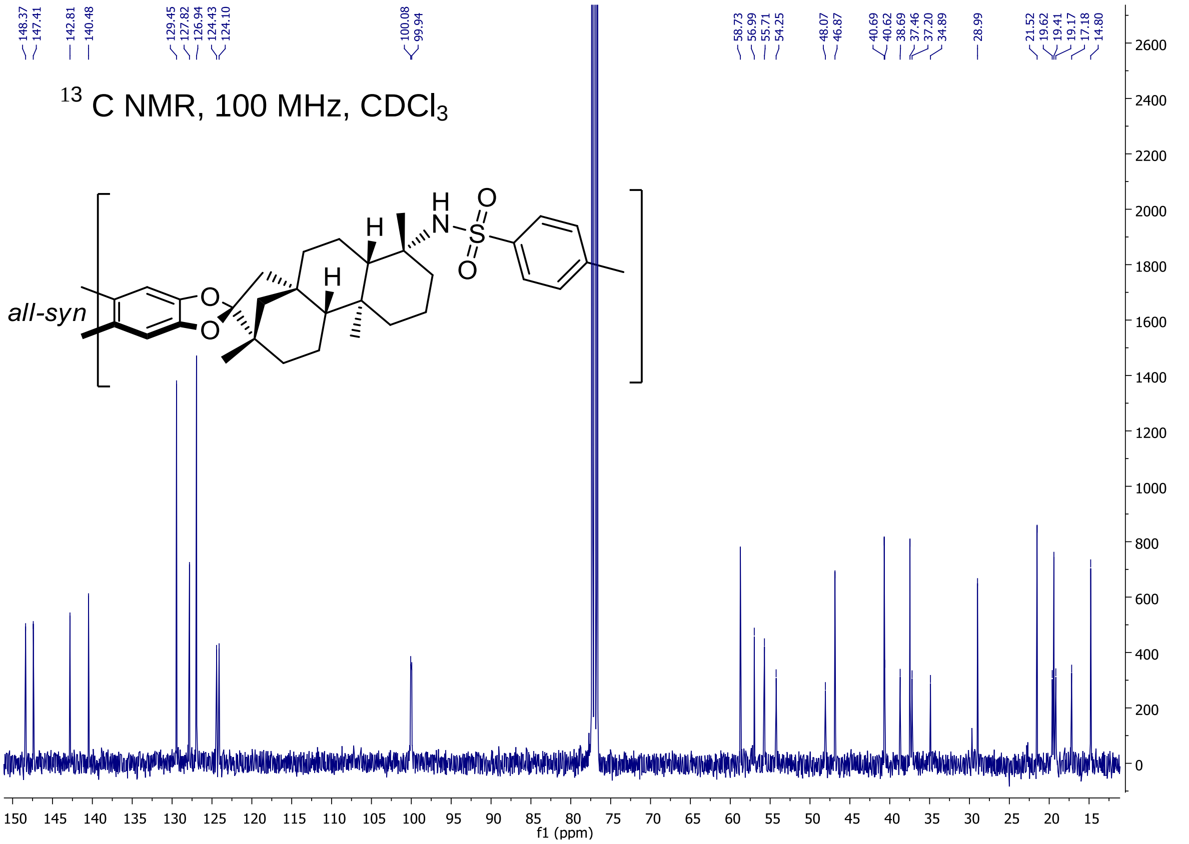

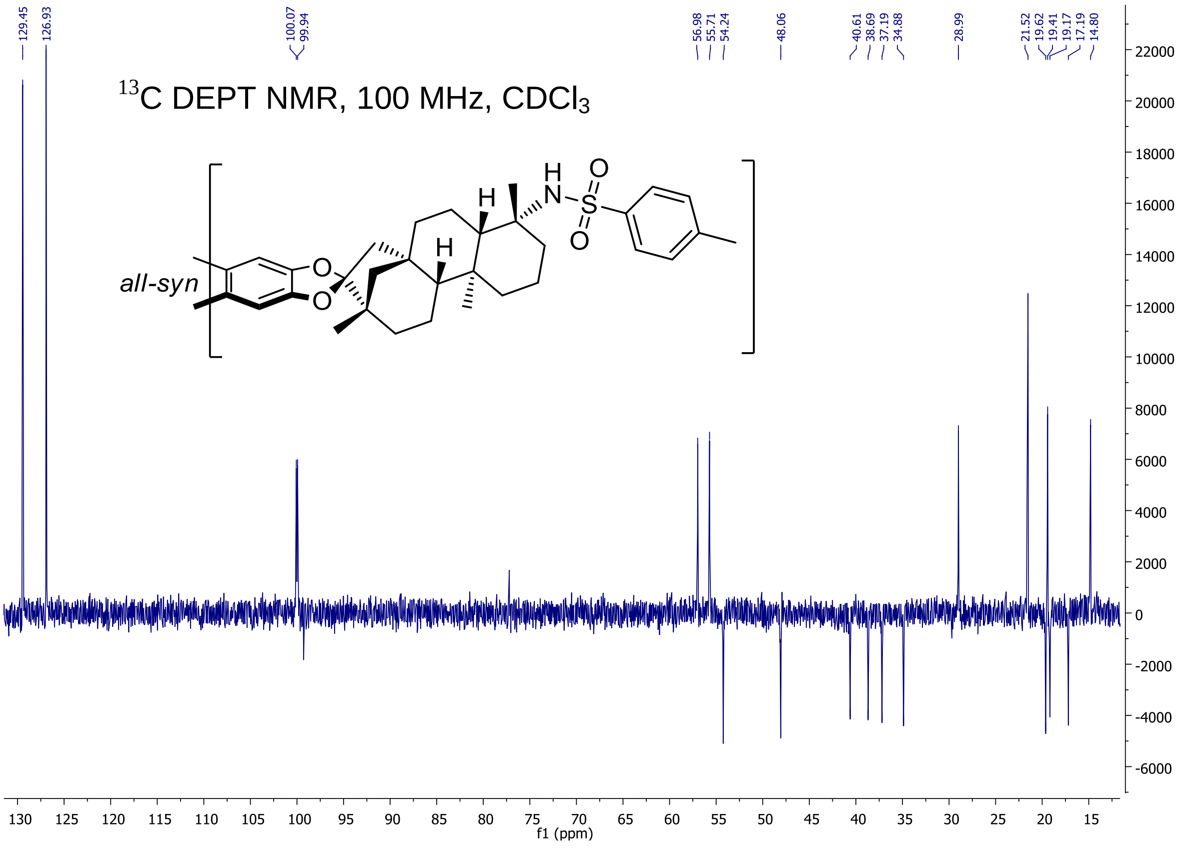
13C DEPT NMR, 100 MHz, CDCl3
all-syn
f1 (ppm)

Compound **6**:

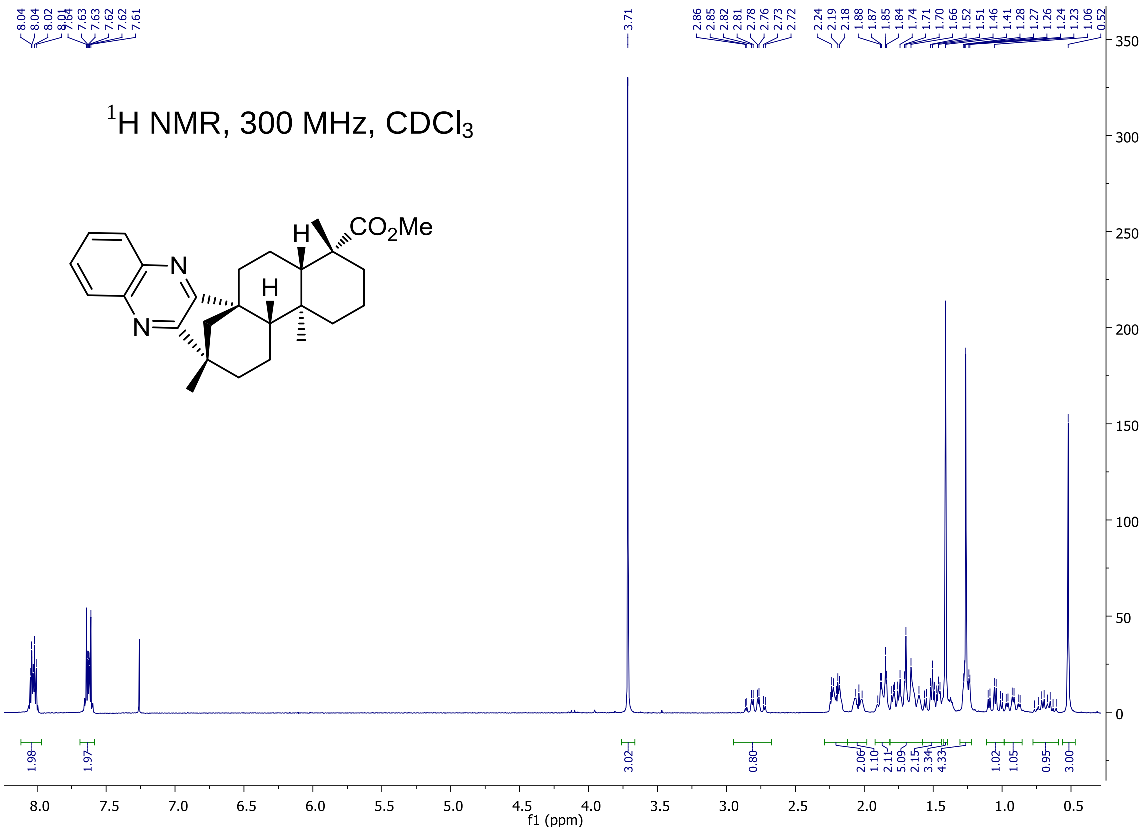
1H NMR, 300 MHz, CDCl3
CO2Me
f1 (ppm)

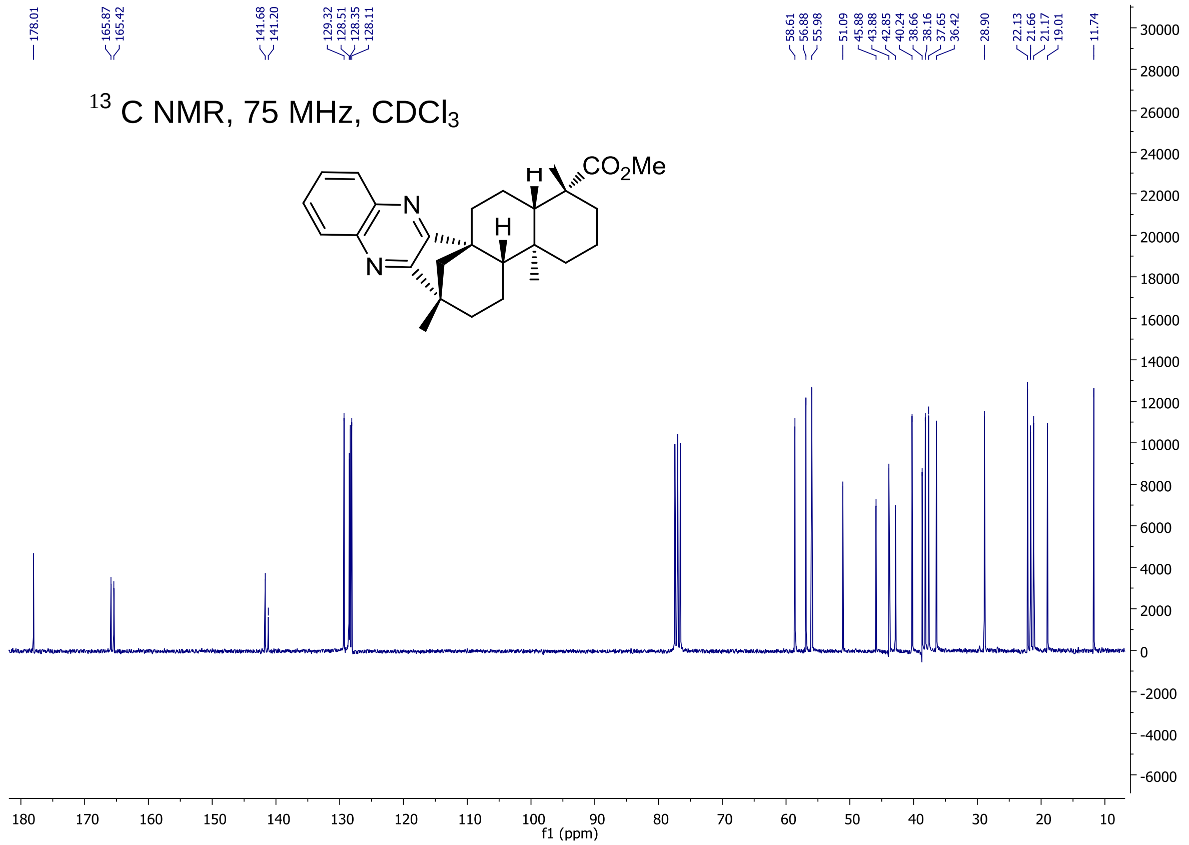
178.01
165.87
165.42
141.68
141.20
129.32
128.51
128.35
128.11
58.61
56.88
55.98
51.09
45.88
43.88
42.85
40.24
38.66
38.16
37.65
36.42
28.90
22.13
21.66
21.17
19.01
11.74
^{13} C NMR, 75 MHz, $CDCl_3$
N
N
H
H
CO_2Me
f1 (ppm)

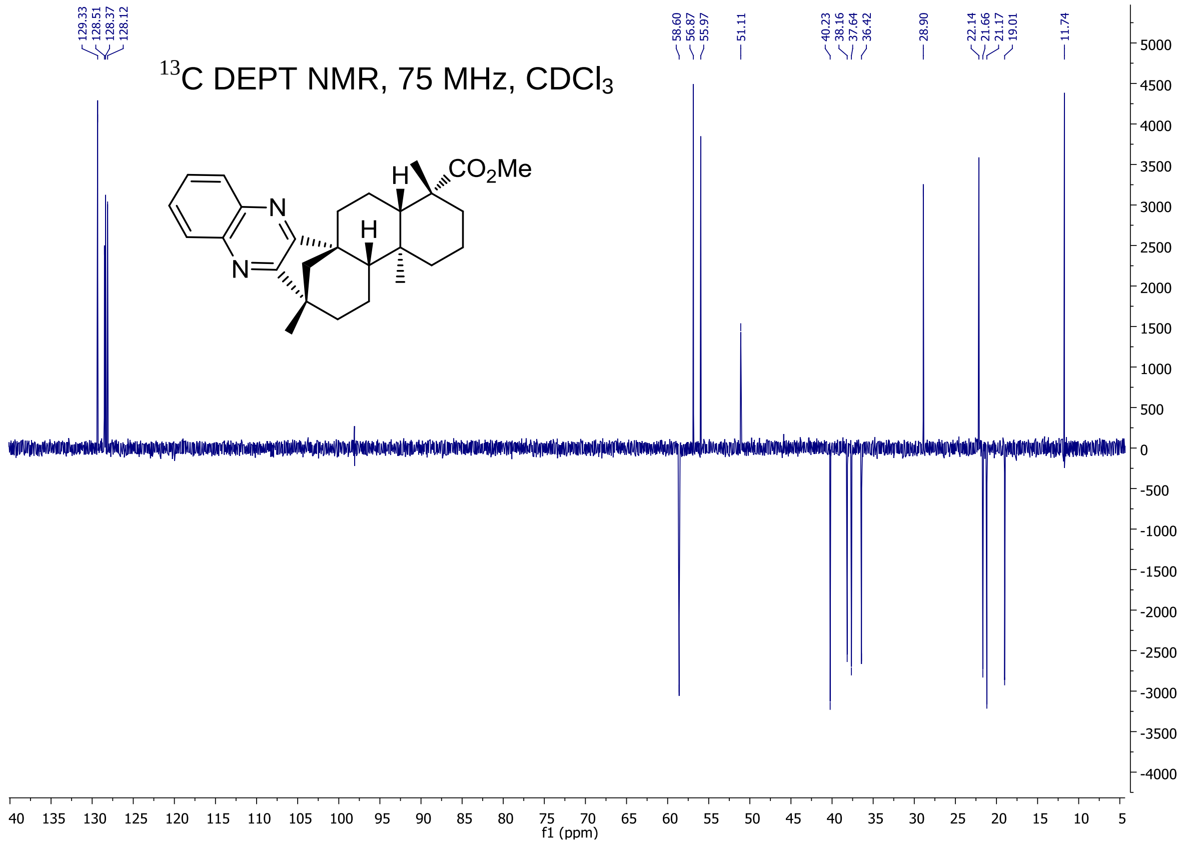
129.33
128.51
128.37
128.12
58.60
56.87
55.97
51.11
40.23
38.16
37.64
36.42
28.90
22.14
21.66
21.17
19.01
11.74
^{13}C DEPT NMR, 75 MHz, $CDCl_3$
N
N
H
H
CO_2Me
f1 (ppm)

Compound **7**:

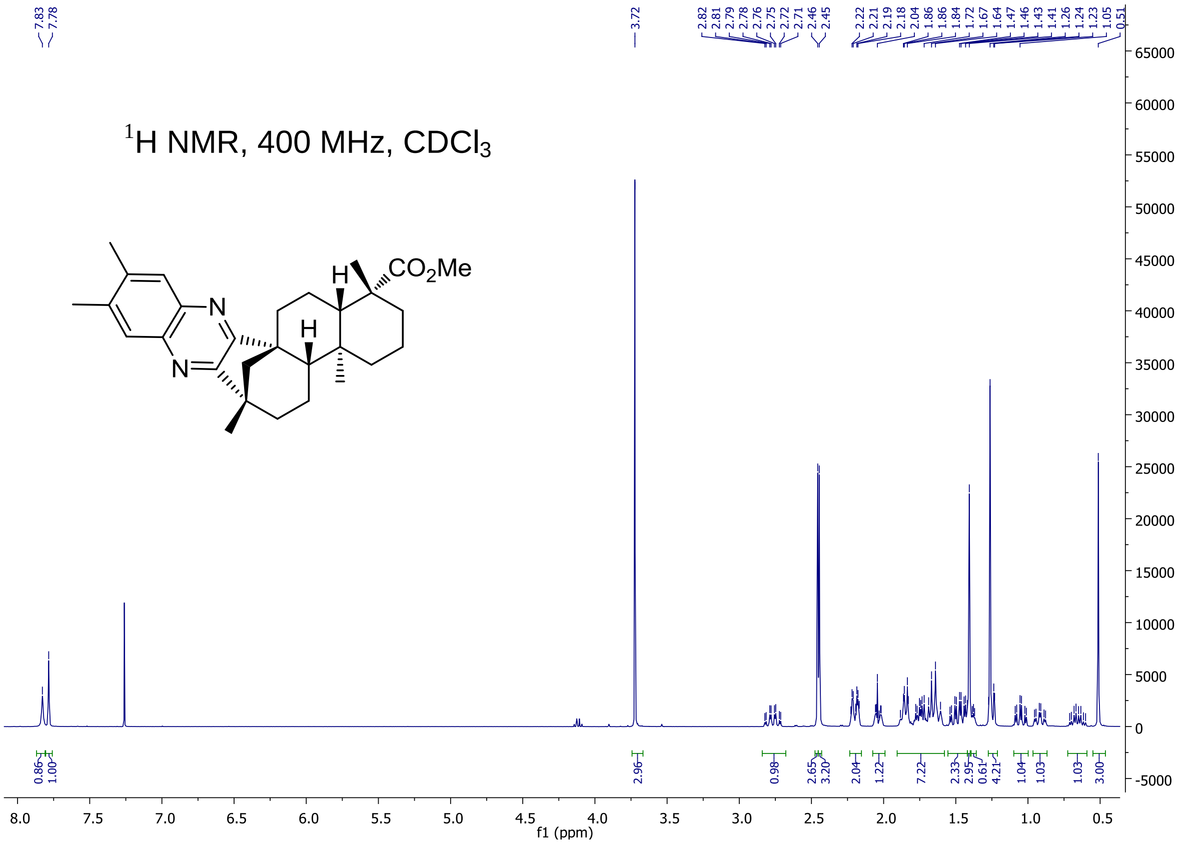

^{1}H NMR, 400 MHz, CDCl$_3$
N
N
H
H
CO$_2$Me
f1 (ppm)

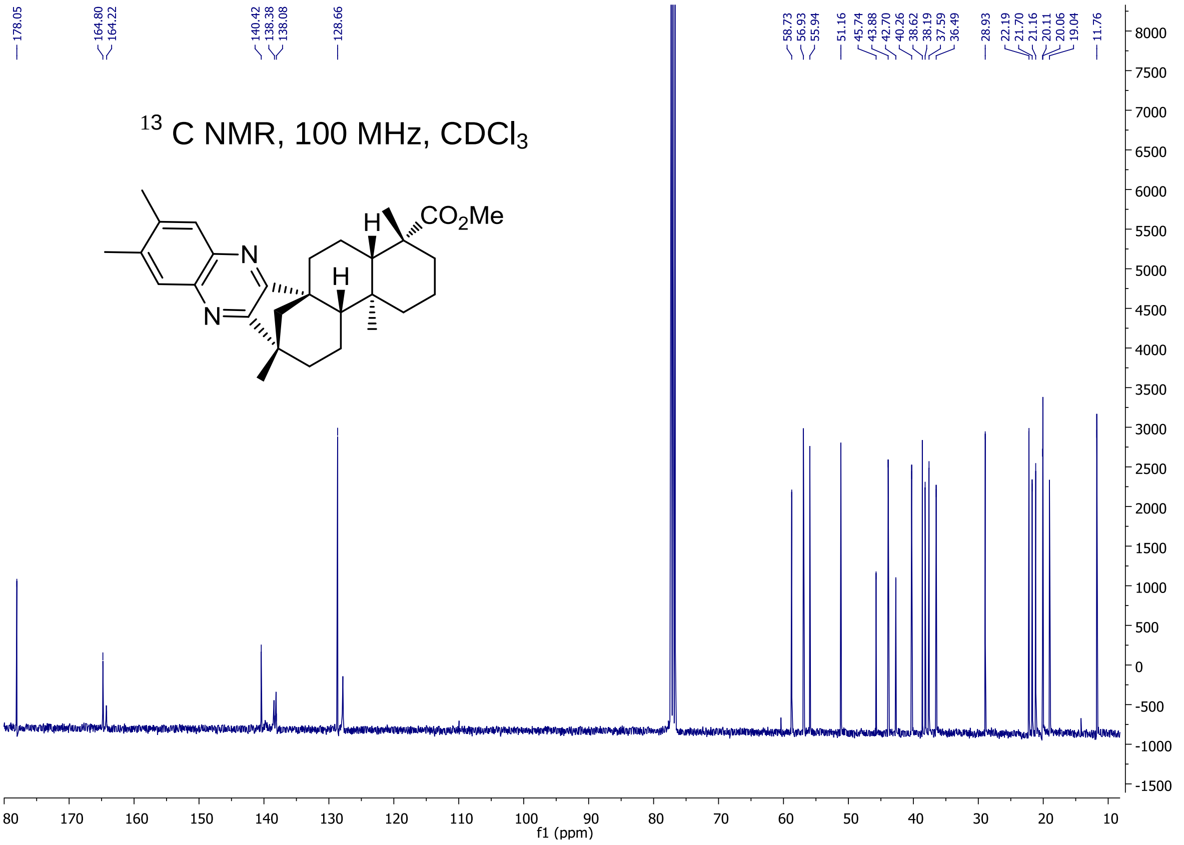

^{13} C NMR, 100 MHz, CDCl$_3$
N
N
H
H
CO$_2$Me
f1 (ppm)

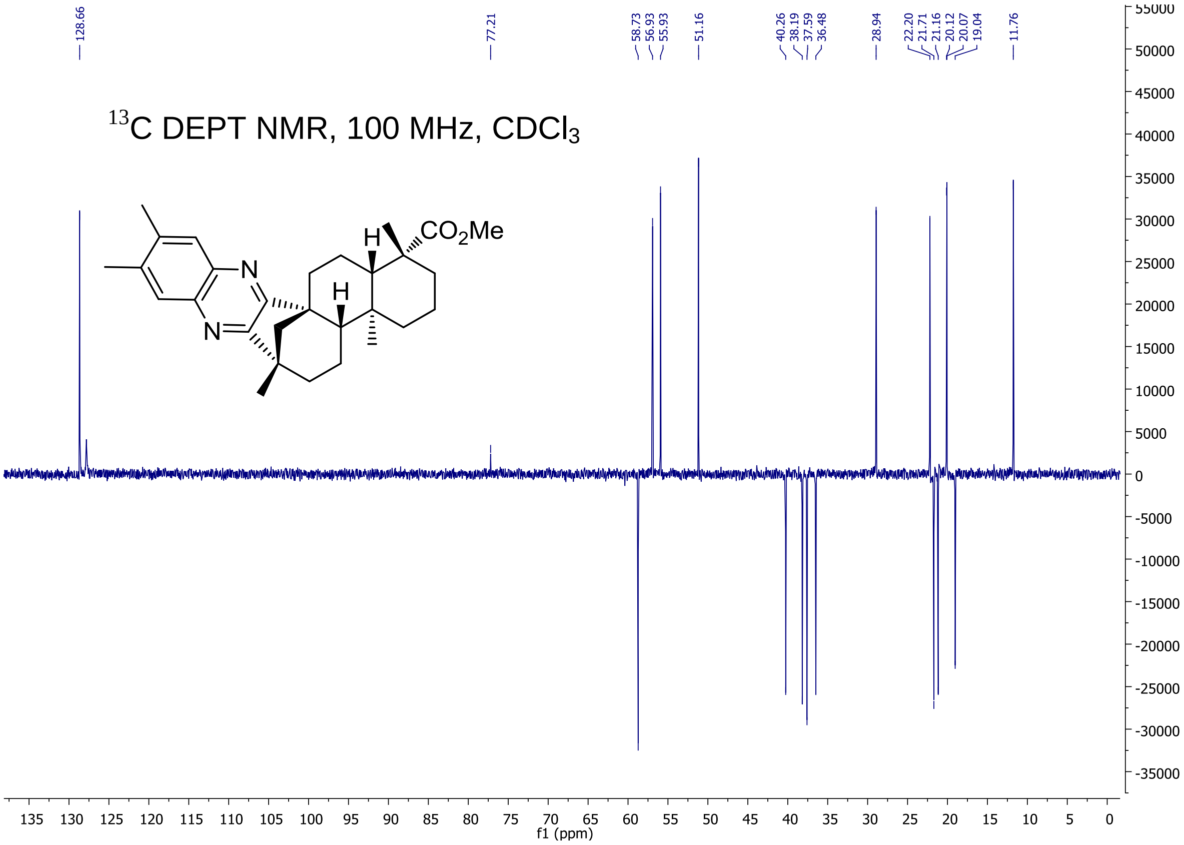
13C DEPT NMR, 100 MHz, CDCl3
CO2Me
f1 (ppm)

Compound **8**:

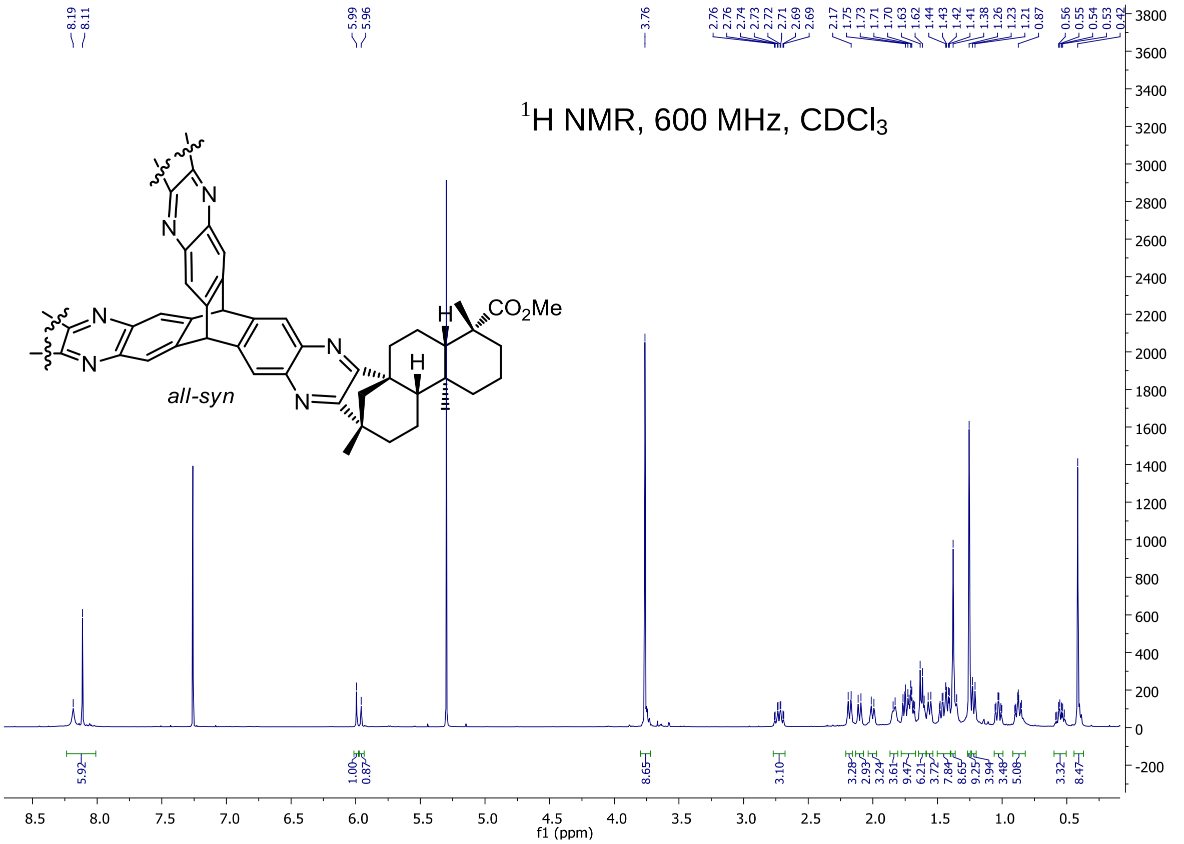
1H NMR, 600 MHz, CDCl3
all-syn
CO2Me
f1 (ppm)

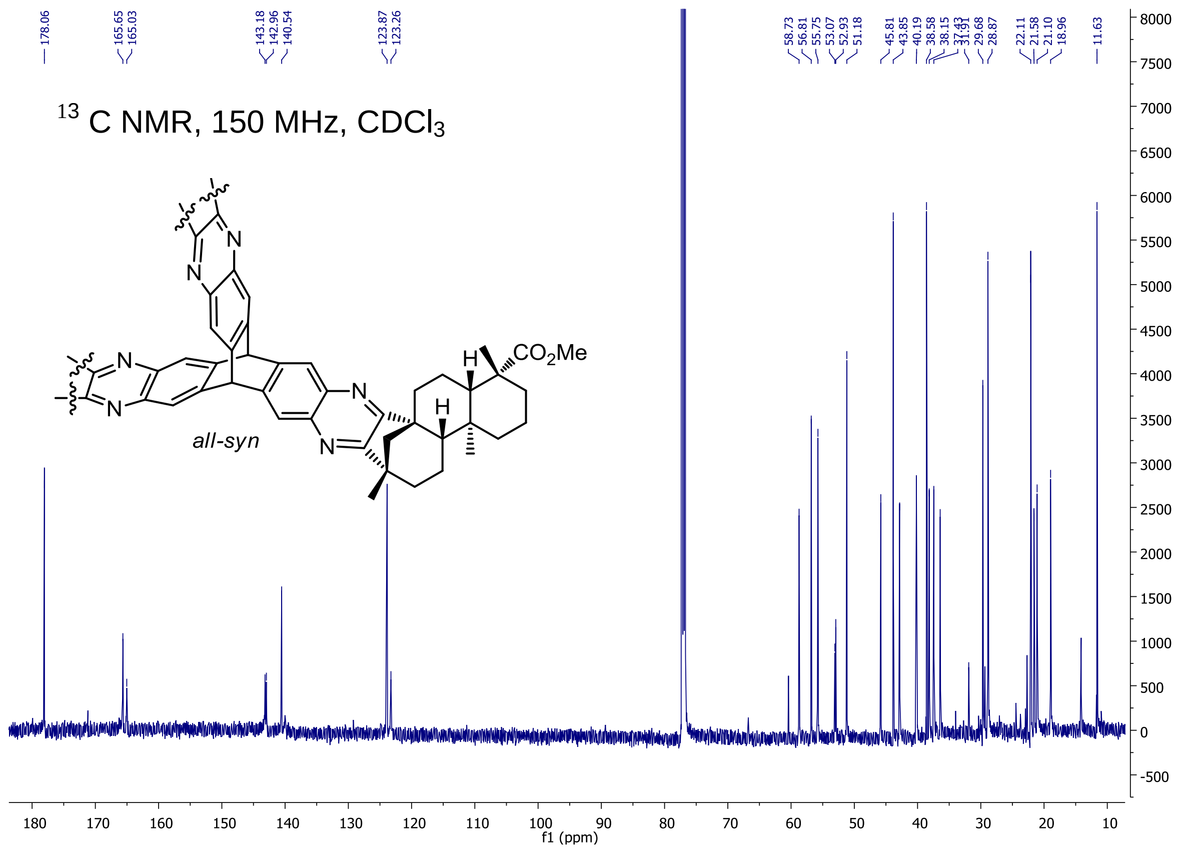
13 C NMR, 150 MHz, CDCl3
all-syn
CO2Me
f1 (ppm)

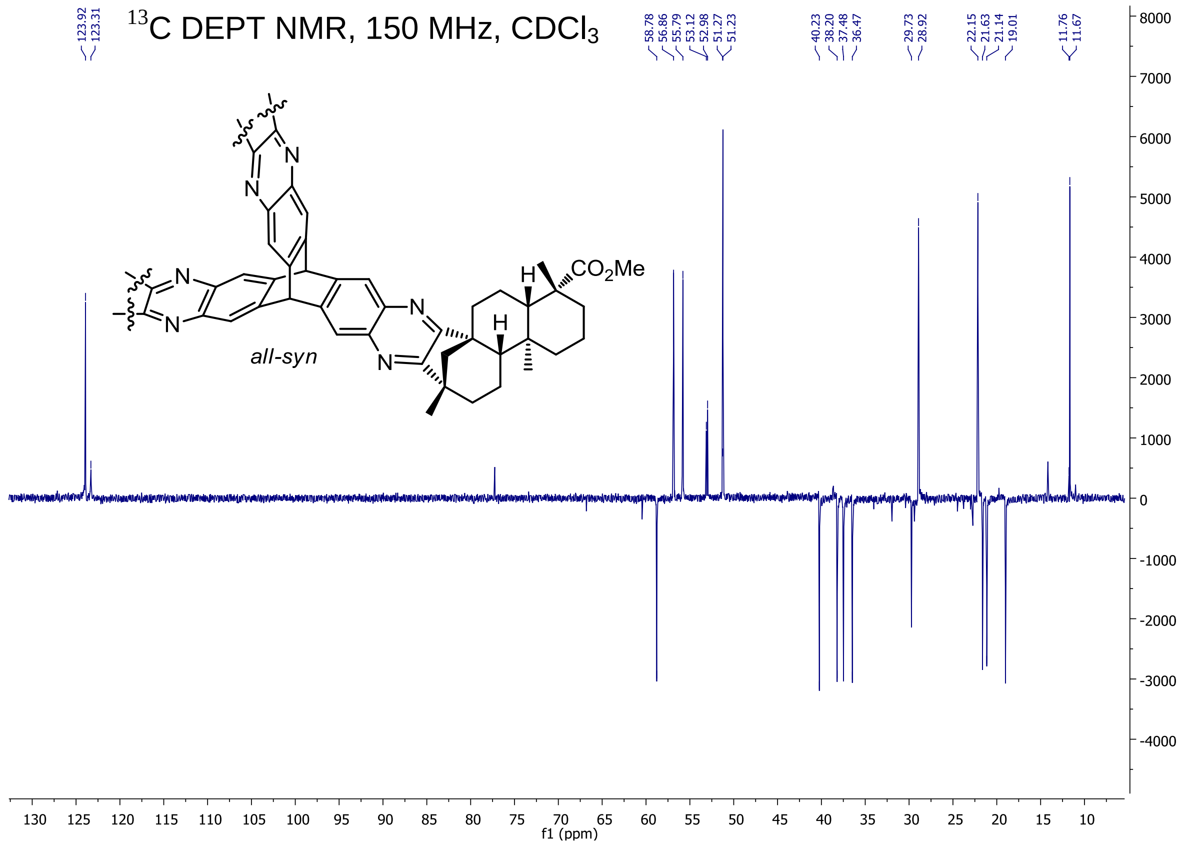
13C DEPT NMR, 150 MHz, CDCl3
all-syn
CO2Me
f1 (ppm)

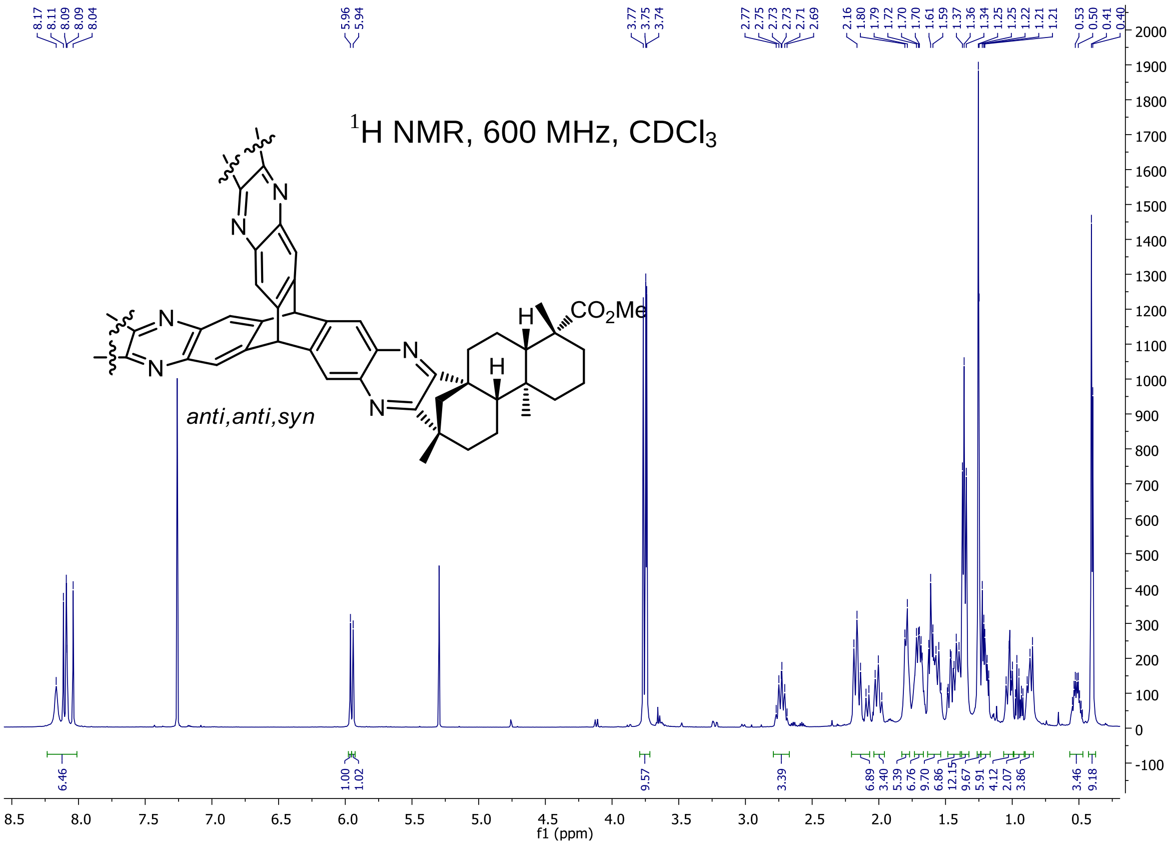
1H NMR, 600 MHz, CDCl3
CO2Me
anti,anti,syn
f1 (ppm)

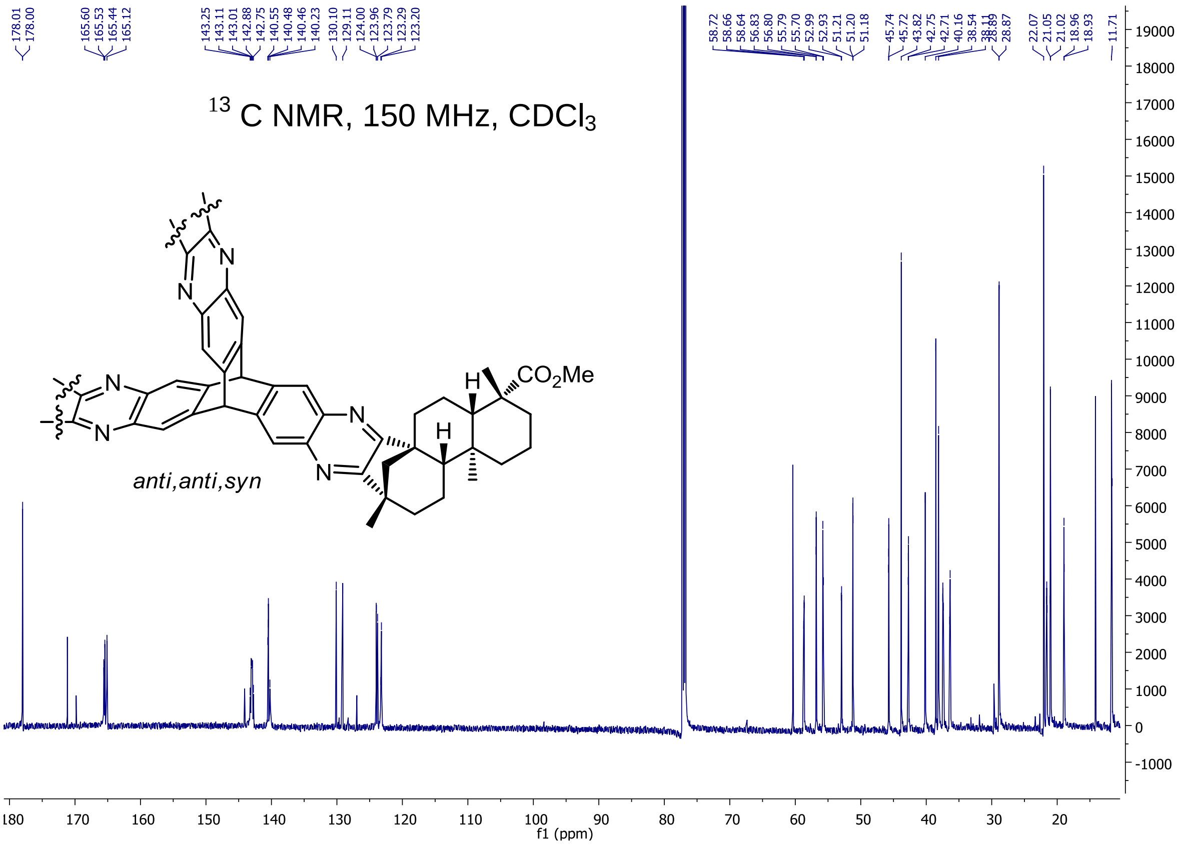
13 C NMR, 150 MHz, CDCl3
CO2Me
anti,anti,syn
f1 (ppm)

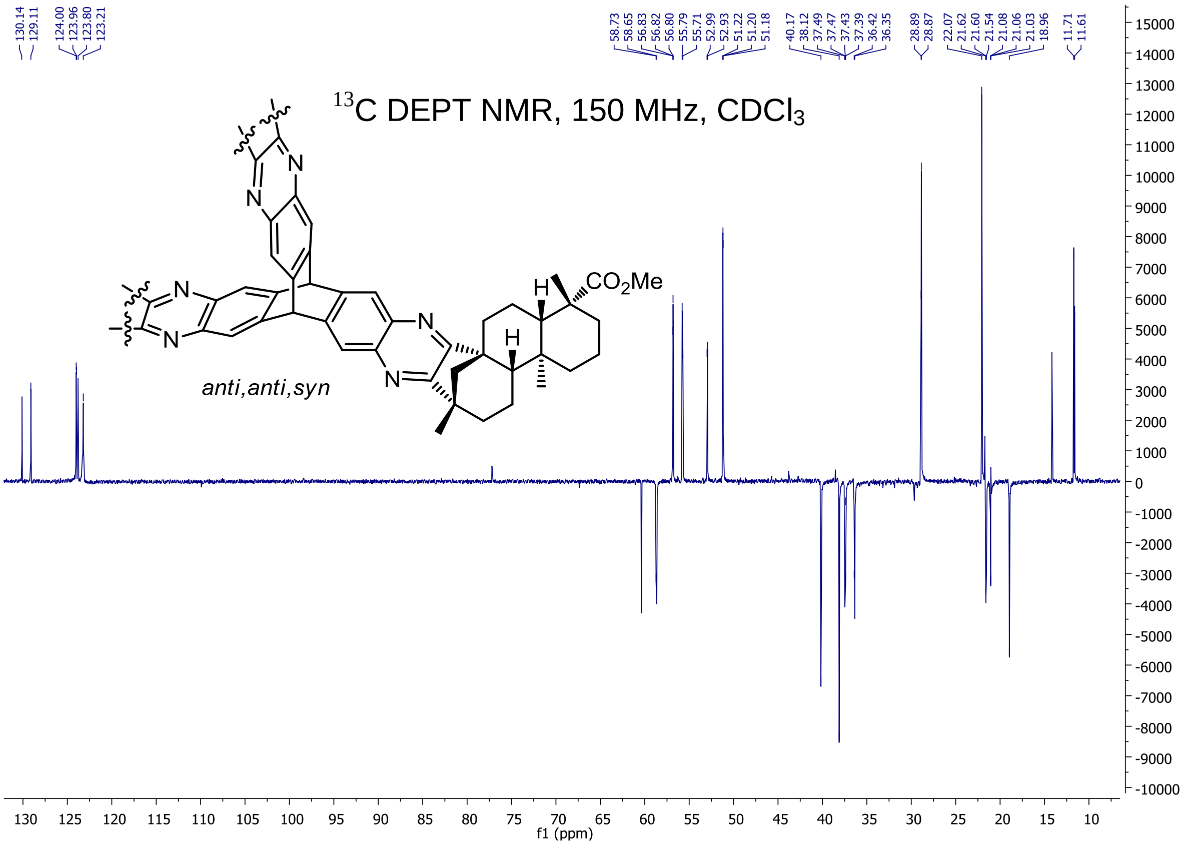
13C DEPT NMR, 150 MHz, CDCl3
anti,anti,syn
CO2Me
f1 (ppm)

Compound **12**:

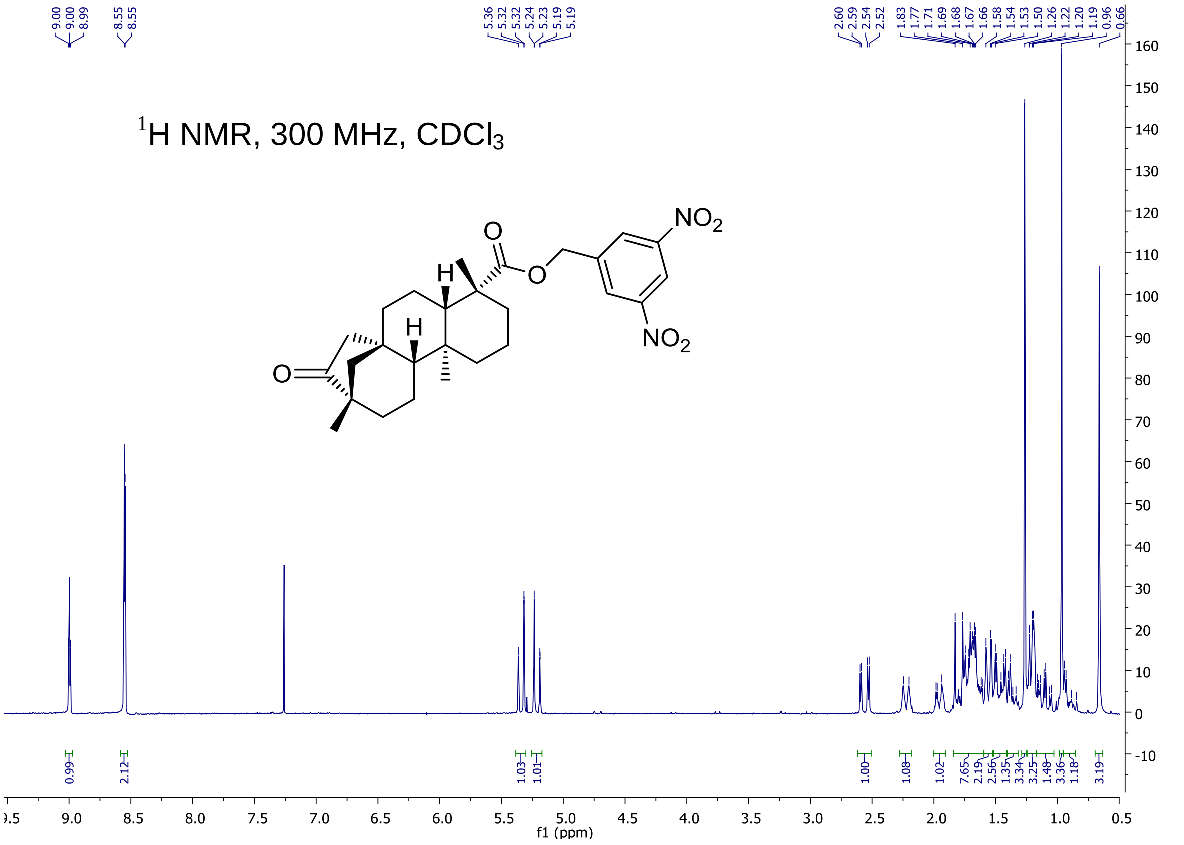
1H NMR, 300 MHz, CDCl3
NO2
NO2
f1 (ppm)

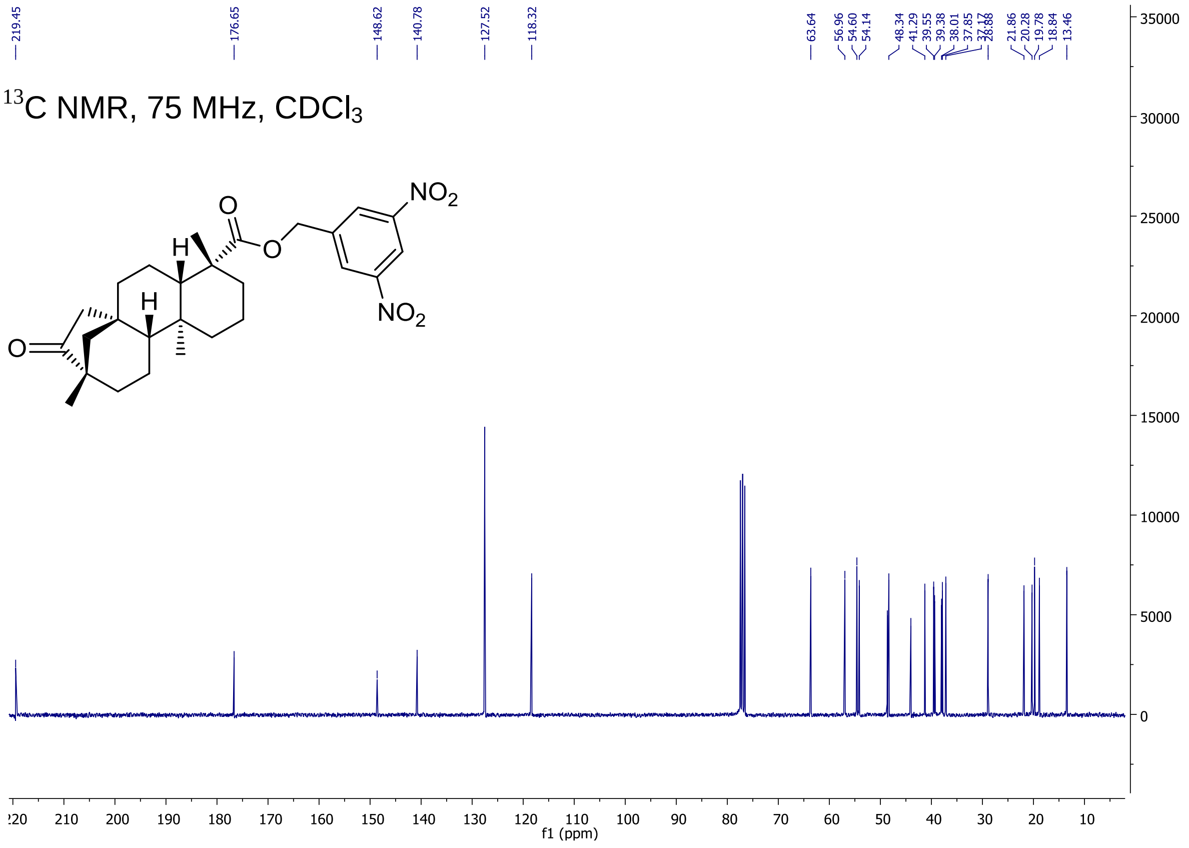

219.45
176.65
148.62
140.78
127.52
118.32
63.64
56.96
54.60
54.14
48.34
41.29
39.55
39.38
38.01
37.85
37.17
28.88
21.86
20.28
19.78
18.84
13.46
^{13}C NMR, 75 MHz, $CDCl_3$
f1 (ppm)

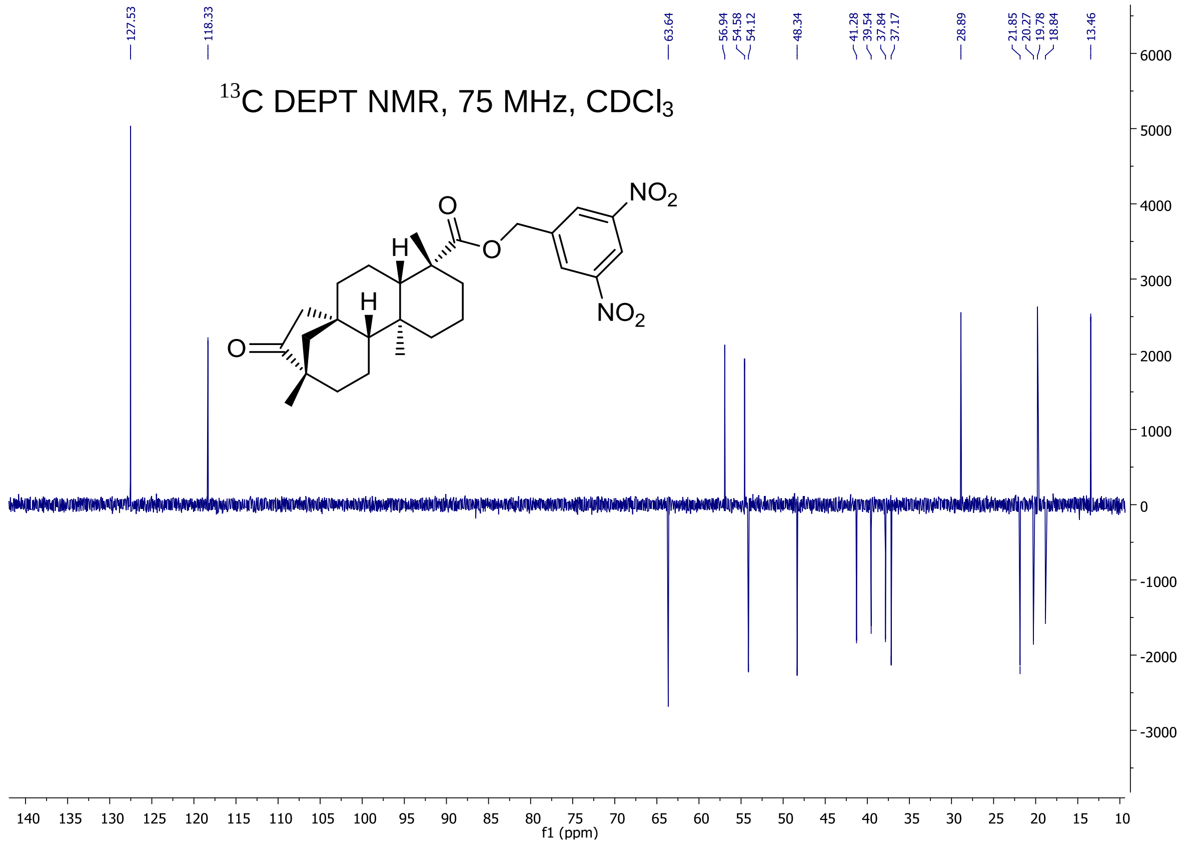

127.53
118.33
63.64
56.94
54.58
54.12
48.34
41.28
39.54
37.84
37.17
28.89
21.85
20.27
19.78
18.84
13.46
^{13}C DEPT NMR, 75 MHz, $CDCl_3$
f1 (ppm)

Compound **10**:

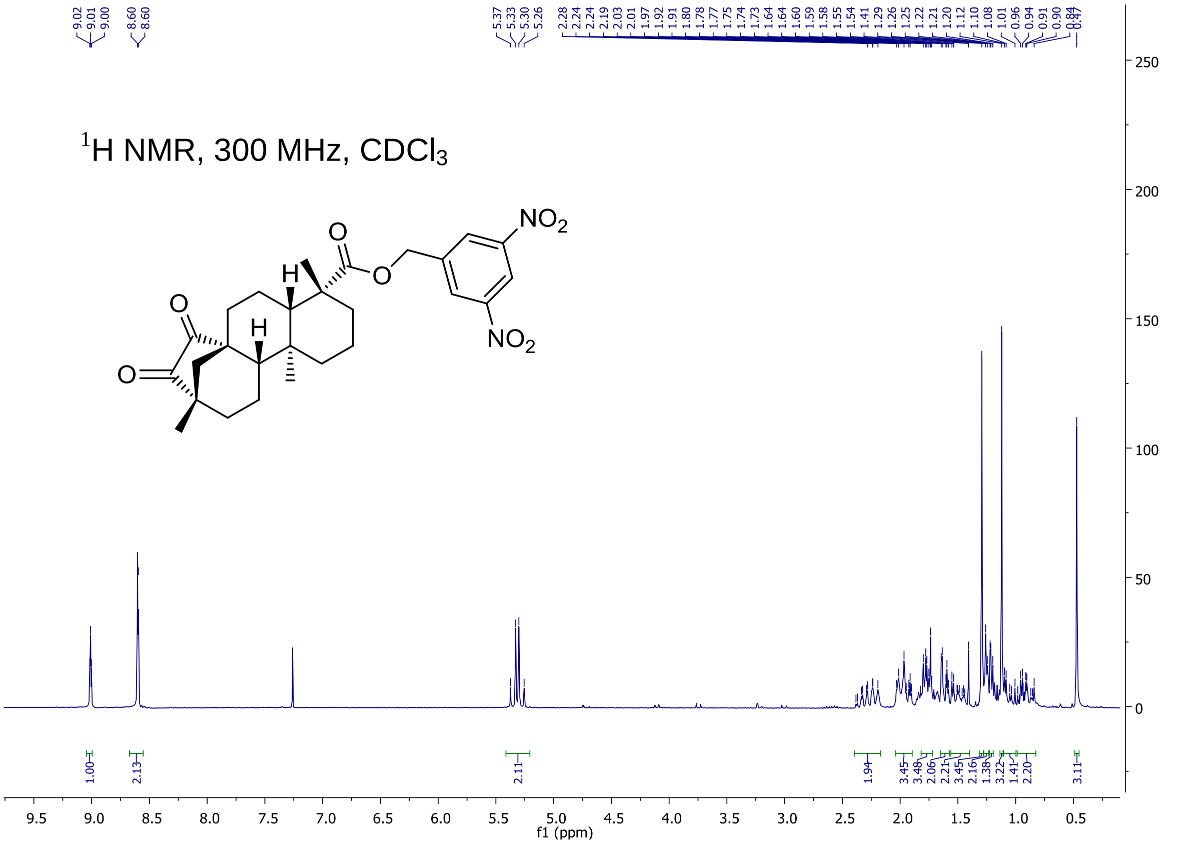

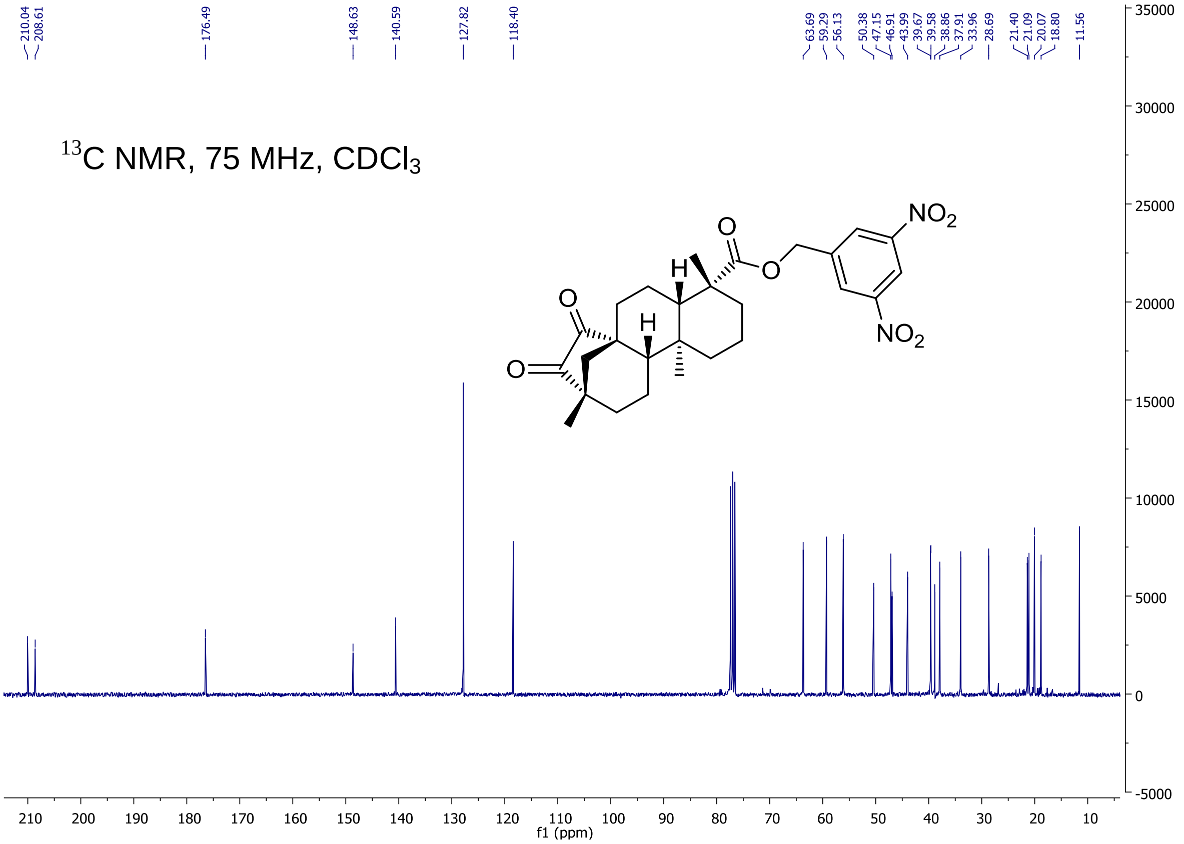

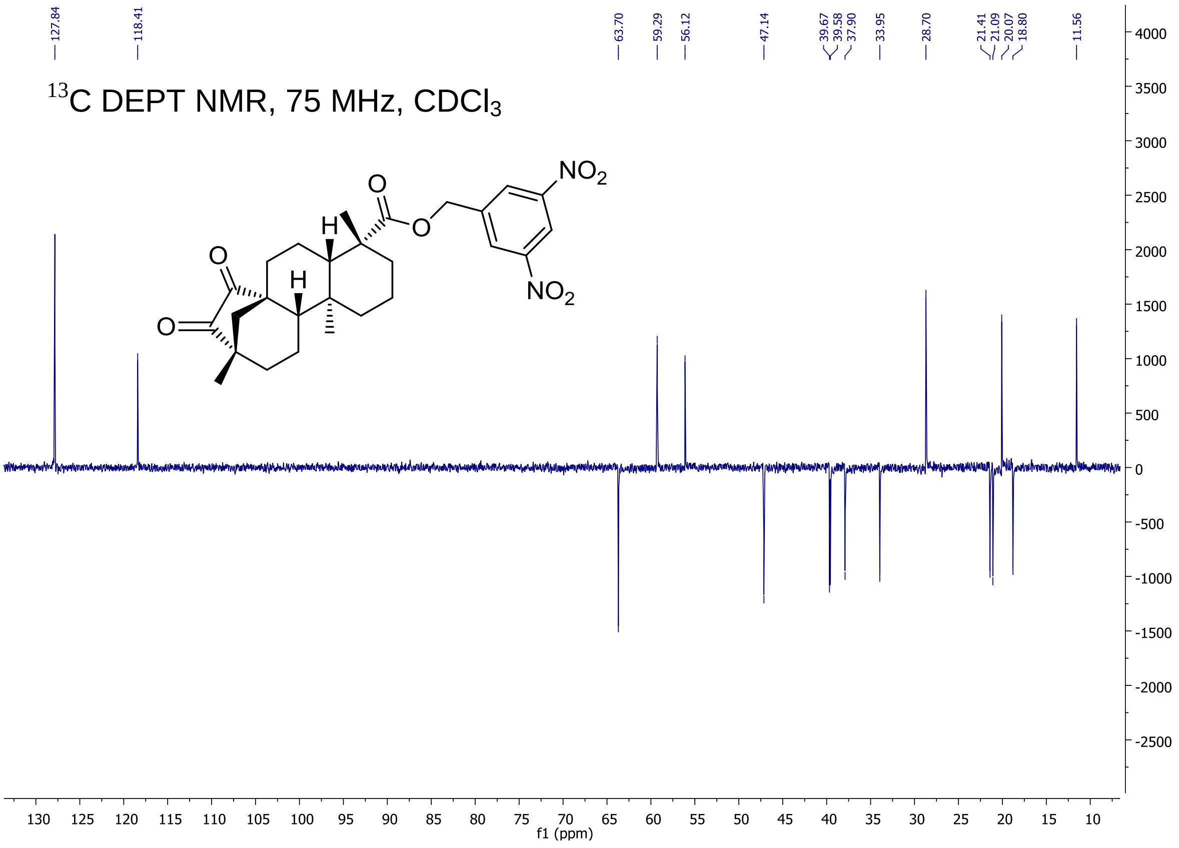
127.84
118.41
63.70
59.29
56.12
47.14
39.67
39.58
37.90
33.95
28.70
21.41
21.09
20.07
18.80
11.56
13C DEPT NMR, 75 MHz, CDCl3
NO2
NO2
O
O
O
O
H
H
f1 (ppm)

Compound **13**:

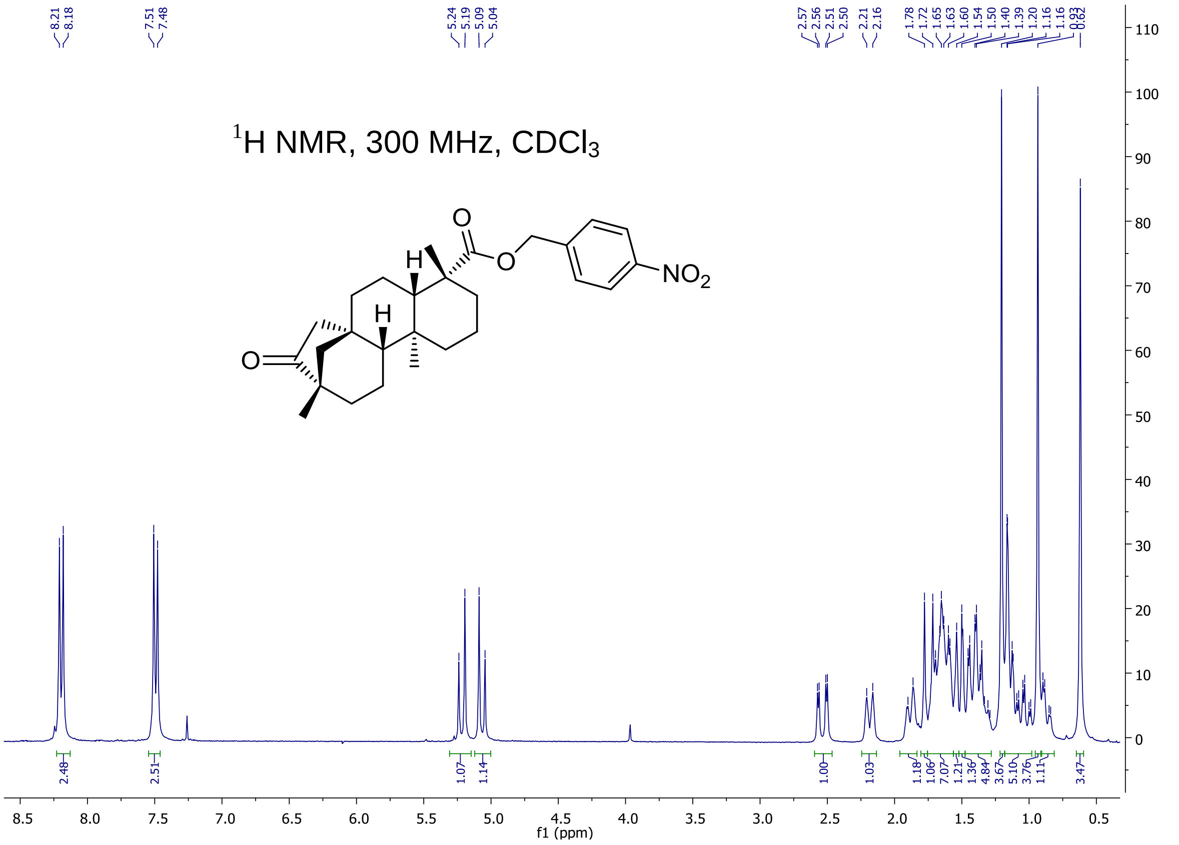
8.21
8.18
7.51
7.48
5.24
5.19
5.09
5.04
2.57
2.56
2.51
2.50
2.21
2.16
1.78
1.72
1.65
1.63
1.60
1.54
1.50
1.40
1.39
1.20
1.16
1.16
0.93
0.62
1H NMR, 300 MHz, CDCl3
NO2
O
O
O
H
H
2.48
2.51
1.07
1.14
1.00
1.03
1.18
1.06
7.07
1.21
1.36
4.84
3.67
5.10
3.76
1.11
3.47
f1 (ppm)

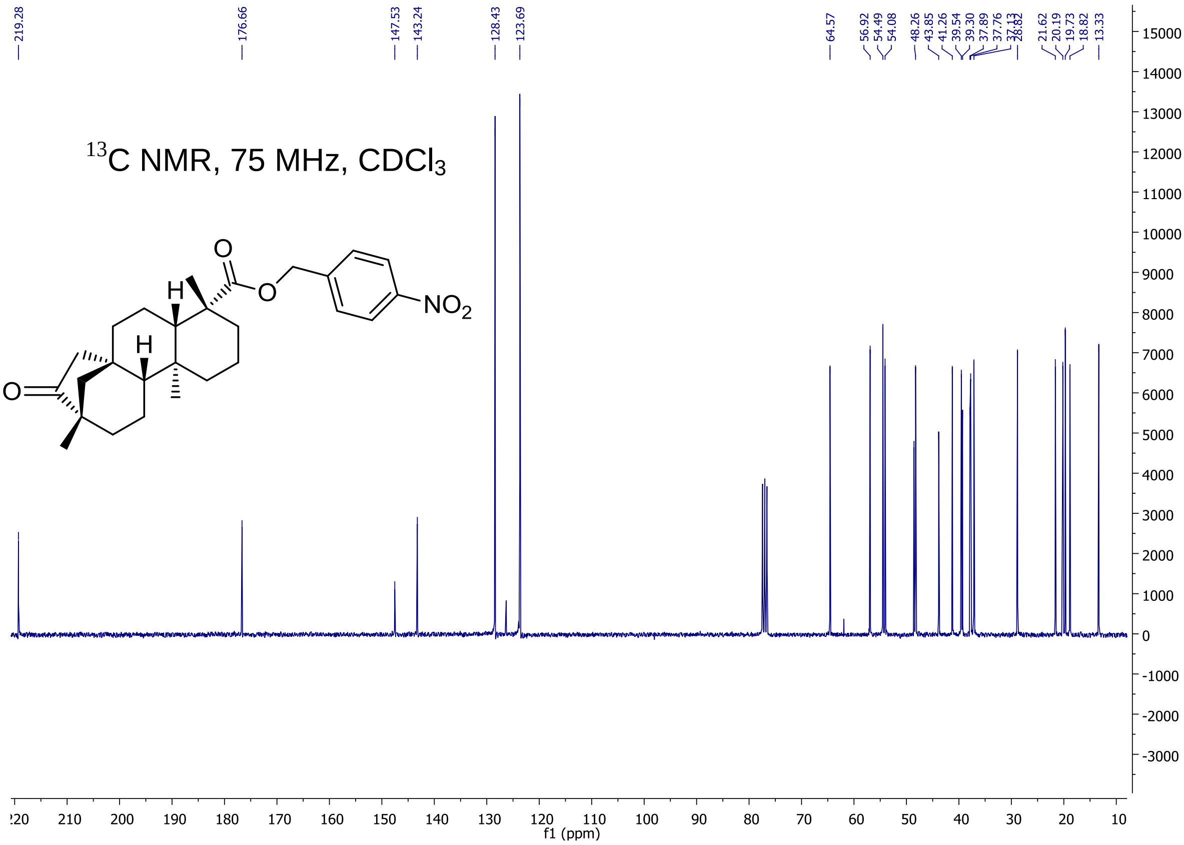
13C NMR, 75 MHz, CDCl3
219.28
176.66
147.53
143.24
128.43
123.69
64.57
56.92
54.49
54.08
48.26
43.85
41.26
39.54
39.30
37.89
37.76
37.13
28.82
21.62
20.19
19.73
18.82
13.33
NO2
f1 (ppm)

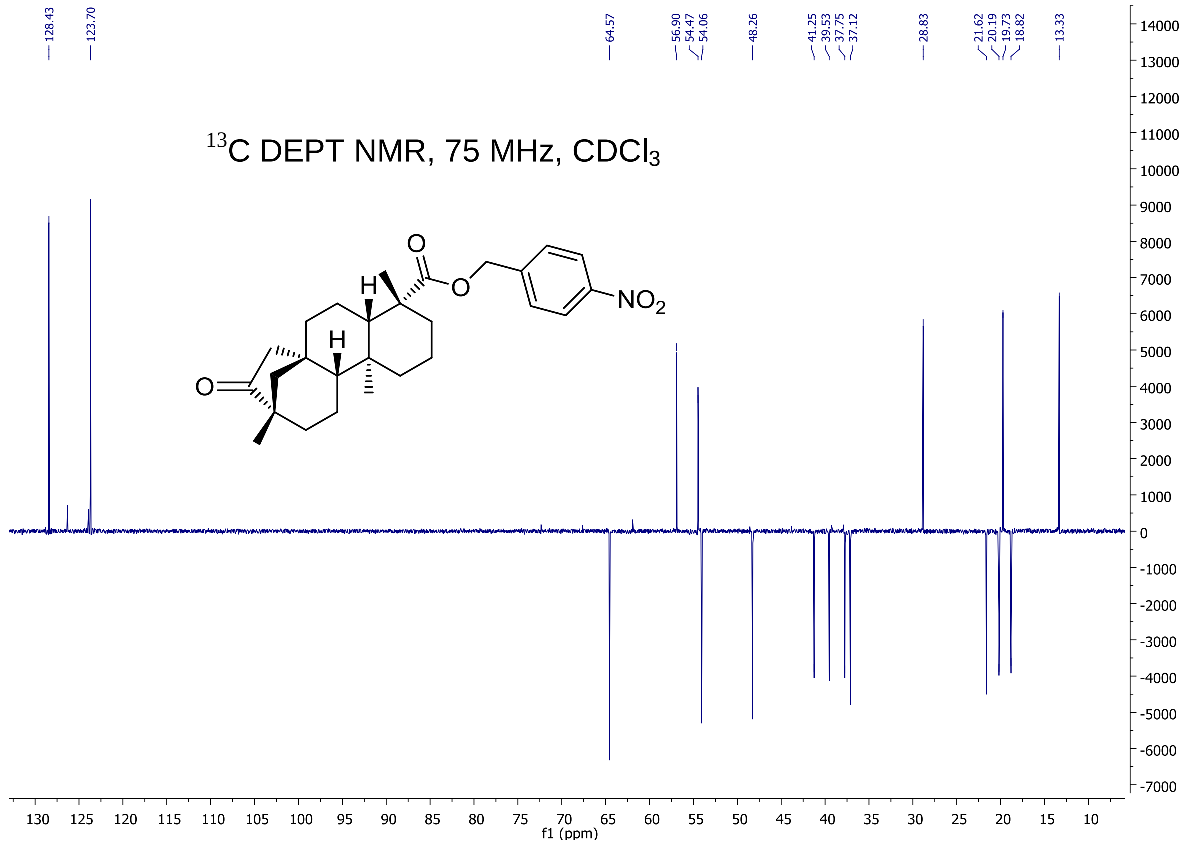
13C DEPT NMR, 75 MHz, CDCl3
128.43
123.70
64.57
56.90
54.47
54.06
48.26
41.25
39.53
37.75
37.12
28.83
21.62
20.19
19.73
18.82
13.33
NO2
f1 (ppm)

Compound **11**:

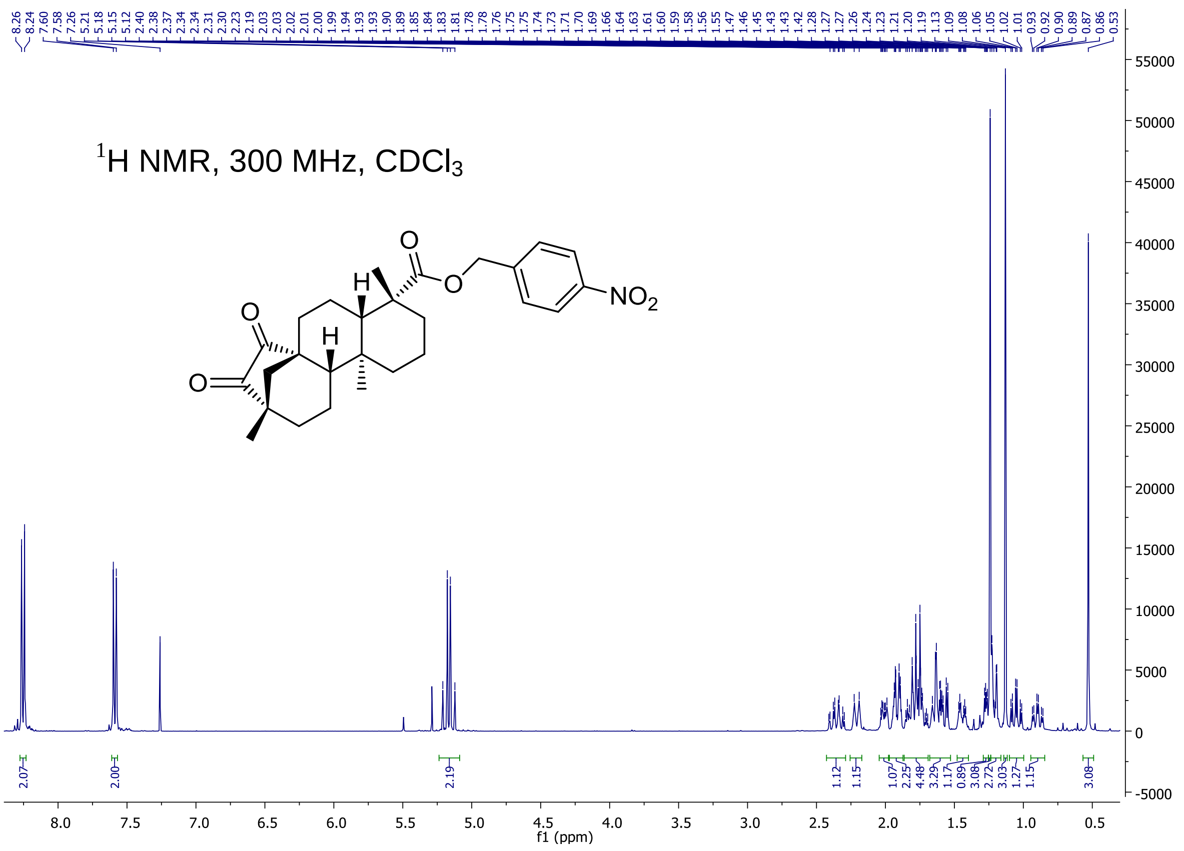

8.26
8.24
7.60
7.58
7.26
5.21
5.18
5.15
5.12
2.40
2.38
2.37
2.34
2.34
2.31
2.30
2.23
2.19
2.03
2.03
2.02
2.01
2.00
1.99
1.94
1.93
1.93
1.90
1.89
1.85
1.84
1.83
1.81
1.78
1.78
1.76
1.75
1.75
1.74
1.73
1.71
1.70
1.69
1.66
1.64
1.63
1.61
1.60
1.59
1.58
1.56
1.55
1.47
1.46
1.45
1.43
1.42
1.28
1.27
1.27
1.26
1.24
1.23
1.21
1.20
1.19
1.13
1.09
1.08
1.06
1.05
1.02
1.01
0.93
0.92
0.90
0.89
0.87
0.86
0.53
^{1}H NMR, 300 MHz, $CDCl_3$
O
O
H
H
O
O
NO_2
2.07
2.00
2.19
1.12
1.15
1.07
2.25
4.48
3.29
1.17
0.89
3.08
2.72
3.03
1.27
1.15
3.08
f1 (ppm)

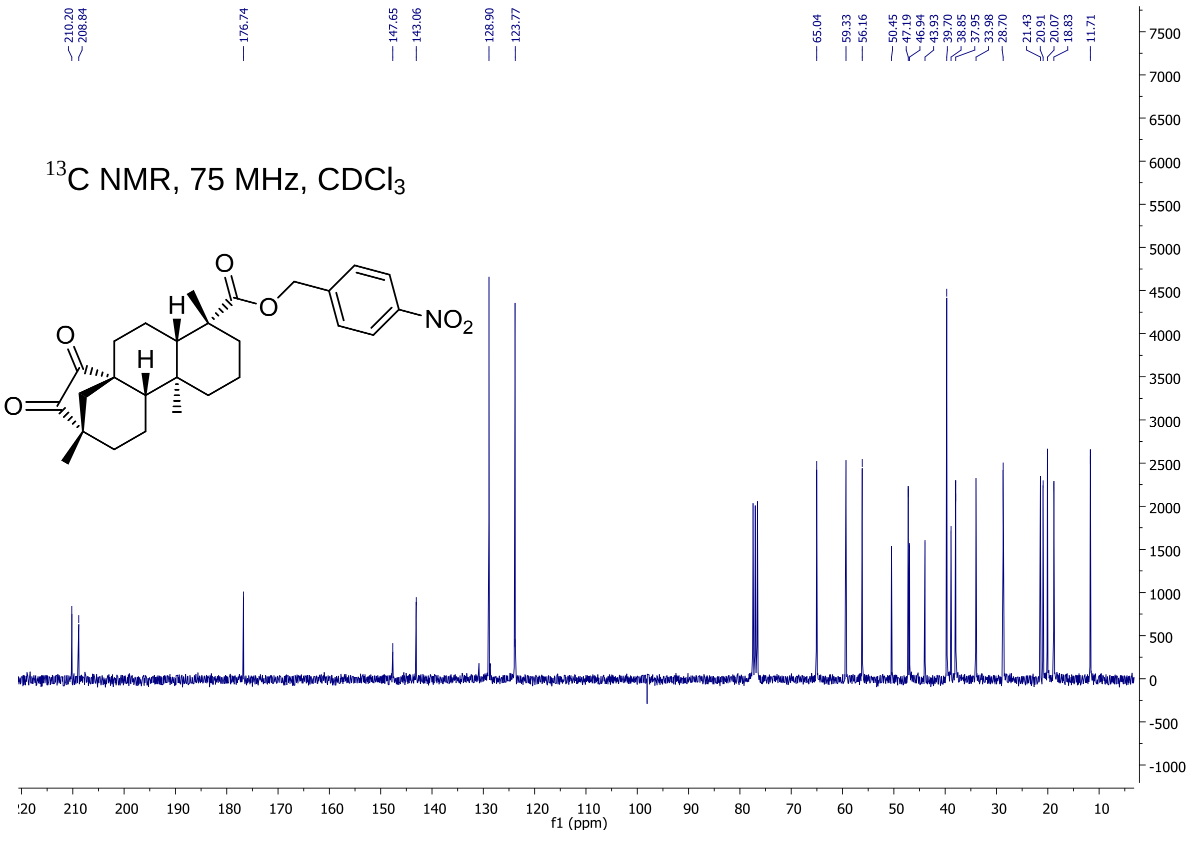

210.20
208.84
176.74
147.65
143.06
128.90
123.77
65.04
59.33
56.16
50.45
47.19
46.94
43.93
39.70
38.85
37.95
33.98
28.70
21.43
20.91
20.07
18.83
11.71
^{13}C NMR, 75 MHz, $CDCl_3$
O
O
H
H
O
O
NO_2
f1 (ppm)

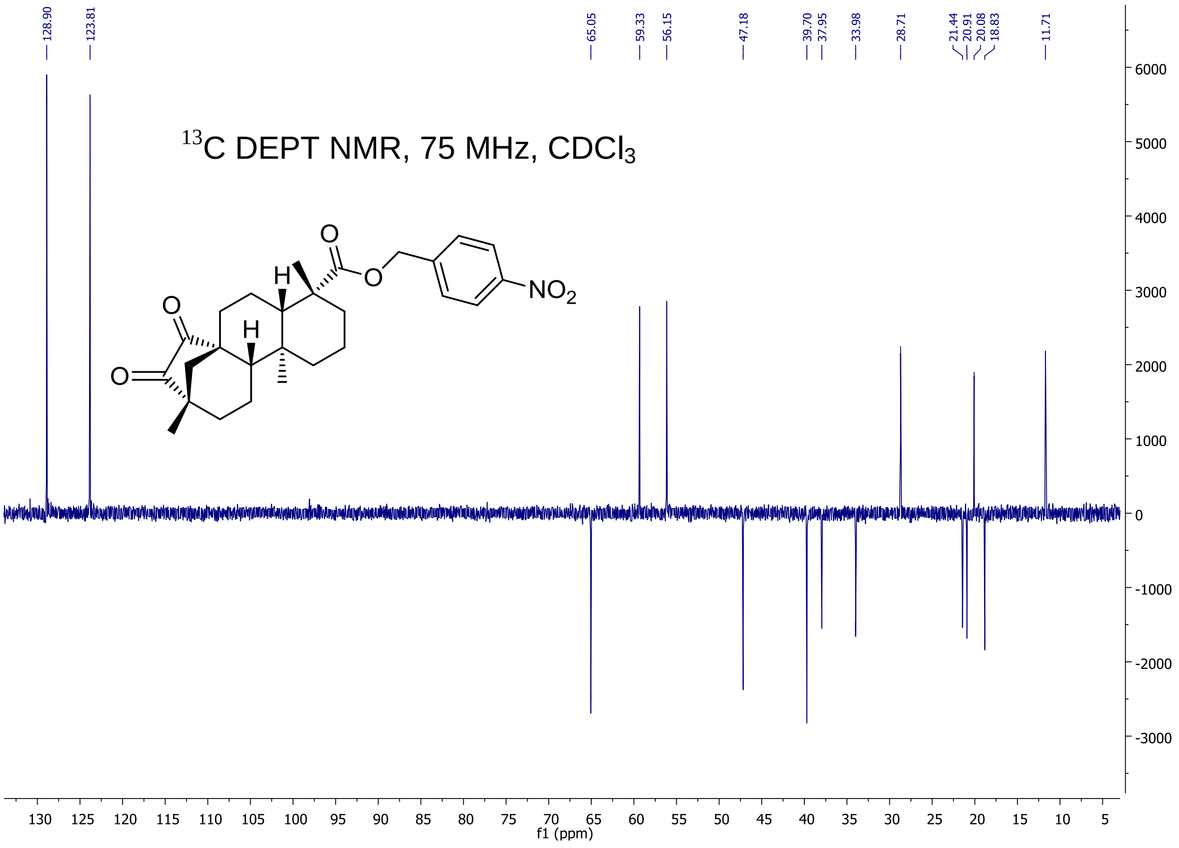

128.90
123.81
65.05
59.33
56.15
47.18
39.70
37.95
33.98
28.71
21.44
20.91
20.08
18.83
11.71
13C DEPT NMR, 75 MHz, CDCl3
f1 (ppm)

Compound **14**:

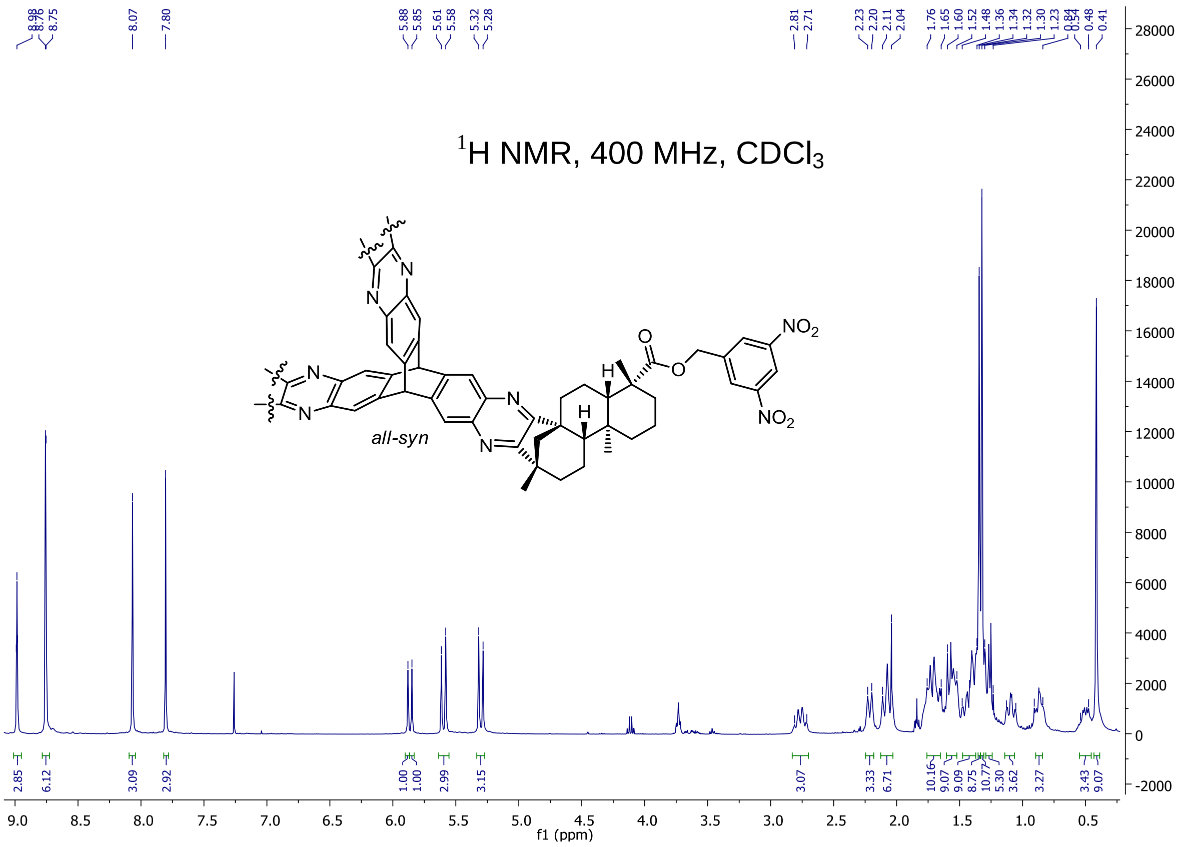

1H NMR, 400 MHz, CDCl3
all-syn
f1 (ppm)

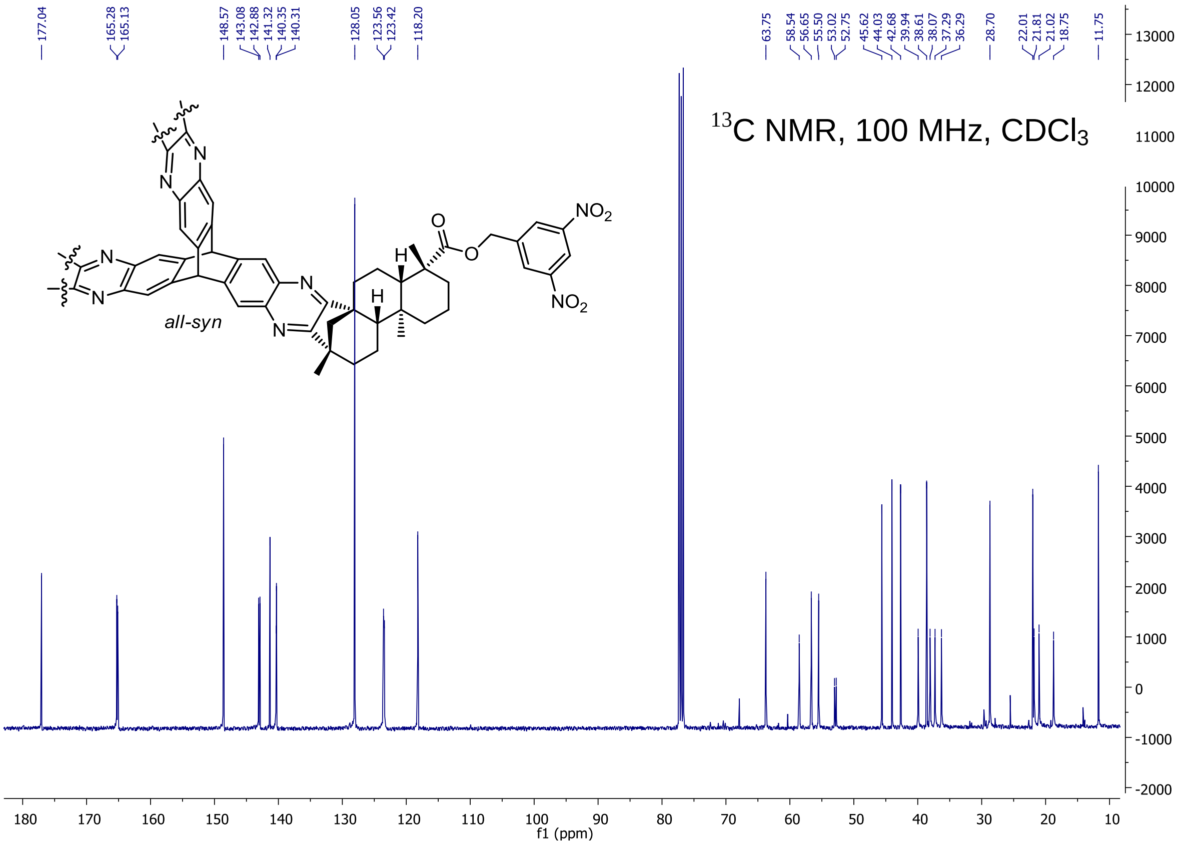
177.04
165.28
165.13
148.57
143.08
142.88
141.32
140.35
140.31
128.05
123.56
123.42
118.20
63.75
58.54
56.65
55.50
53.02
52.75
45.62
44.03
42.68
39.94
38.61
38.07
37.29
36.29
28.70
22.01
21.81
21.02
18.75
11.75
^{13}C NMR, 100 MHz, CDCl$_3$
NO_2
NO_2
all-syn
f1 (ppm)

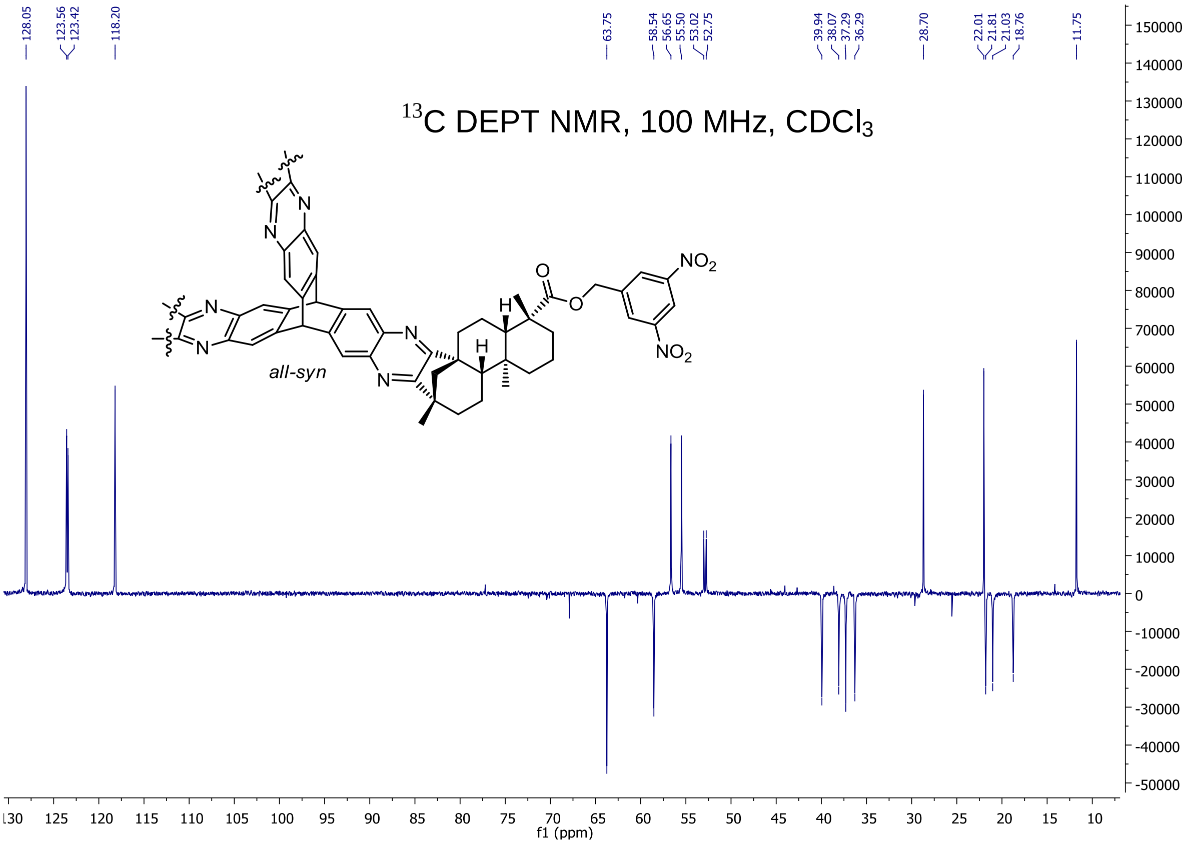
128.05
123.56
123.42
118.20
63.75
58.54
56.65
55.50
53.02
52.75
39.94
38.07
37.29
36.29
28.70
22.01
21.81
21.03
18.76
11.75
^{13}C DEPT NMR, 100 MHz, CDCl$_3$
NO_2
NO_2
all-syn
f1 (ppm)

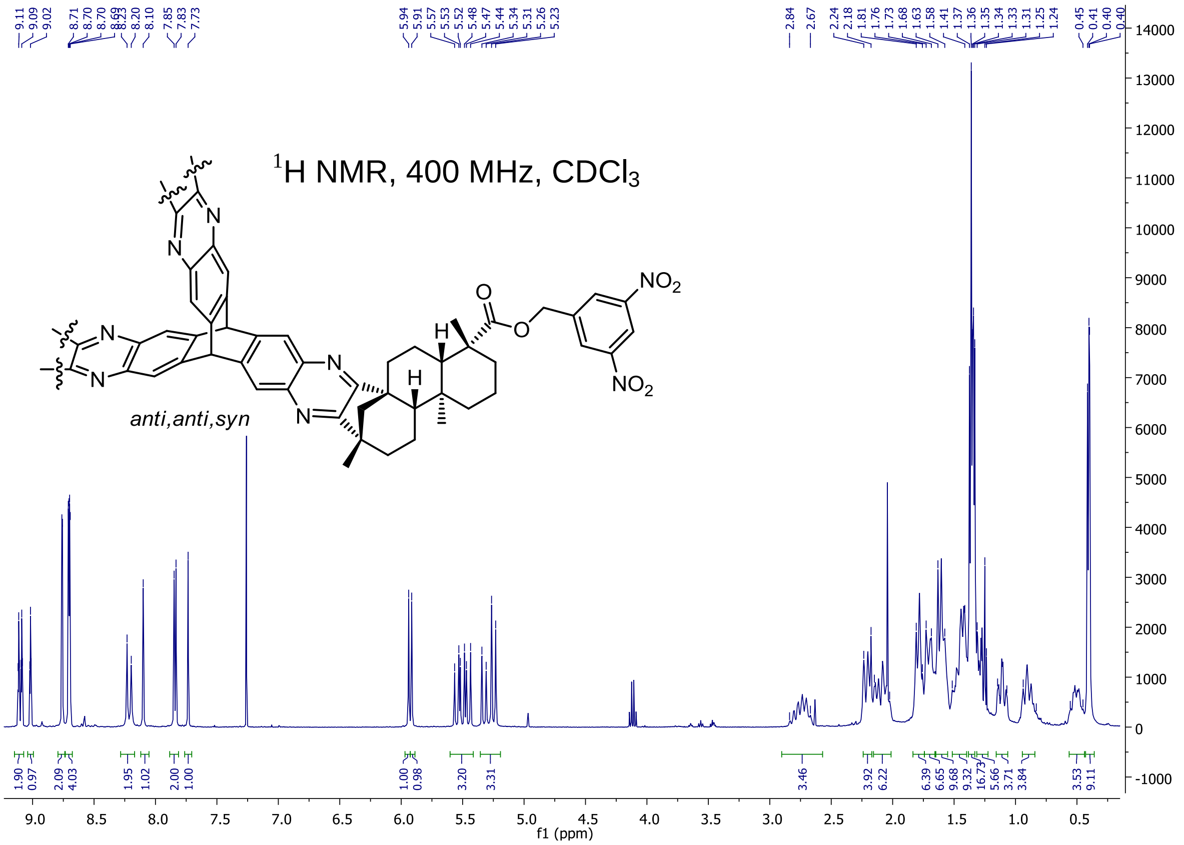

9.11
9.09
9.02
8.71
8.70
8.70
8.69
8.23
8.20
8.10
7.85
7.83
7.73
5.94
5.91
5.57
5.53
5.52
5.48
5.47
5.44
5.34
5.31
5.26
5.23
2.84
2.67
2.24
2.18
1.81
1.76
1.73
1.68
1.63
1.58
1.41
1.37
1.36
1.35
1.34
1.33
1.31
1.25
1.24
0.45
0.41
0.40
0.40
^{1}H NMR, 400 MHz, $CDCl_3$
N
N
N
N
N
N
O
O
NO_2
NO_2
H
H
anti,anti,syn
1.90
0.97
2.09
4.03
1.95
1.02
2.00
1.00
1.00
0.98
3.20
3.31
3.46
3.92
6.22
6.39
6.65
9.68
9.32
16.73
5.66
3.71
3.84
3.53
9.11
f1 (ppm)

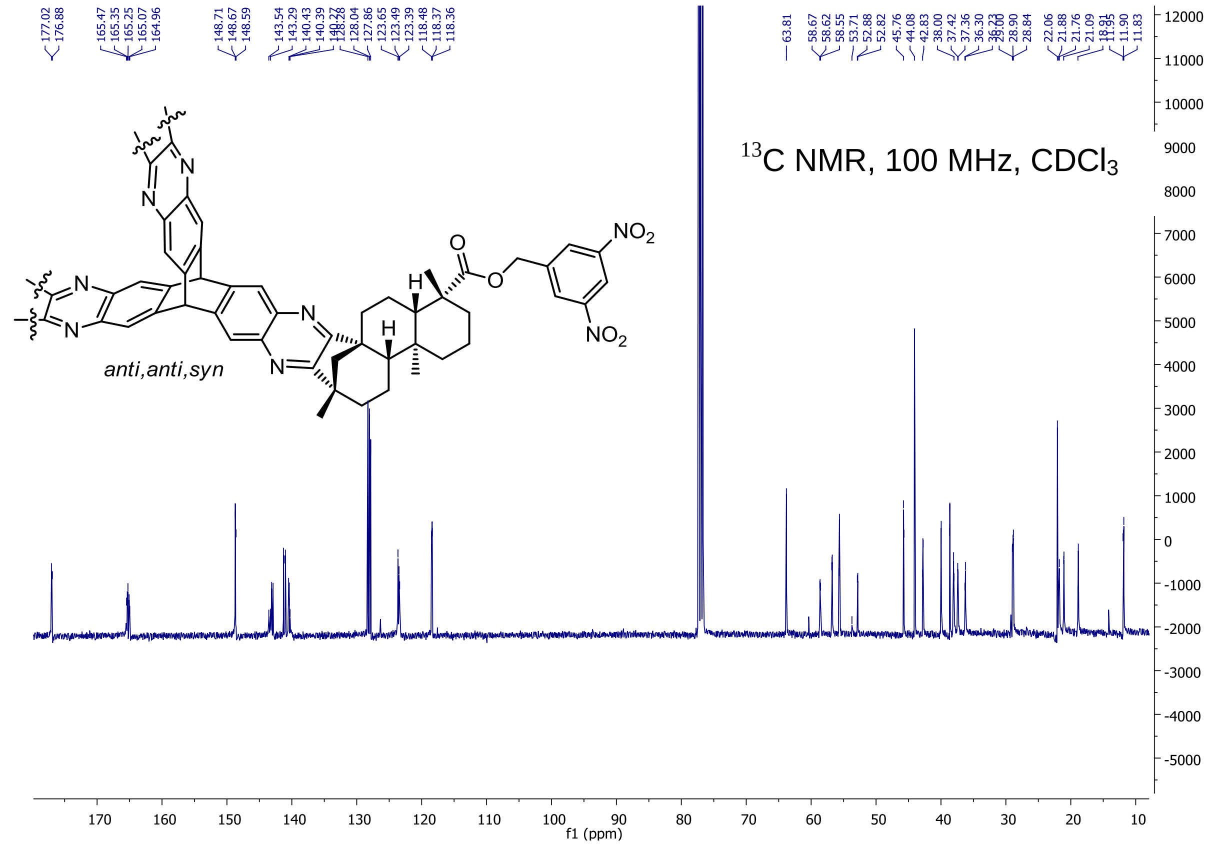

177.02
176.88
165.47
165.35
165.25
165.07
164.96
148.71
148.67
148.59
143.54
143.29
140.43
140.39
140.27
128.28
128.04
127.86
123.65
123.49
123.39
118.48
118.37
118.36
63.81
58.67
58.62
58.55
53.71
52.88
52.82
45.76
44.08
42.83
38.00
37.42
37.36
36.30
36.23
29.00
28.90
28.84
22.06
21.88
21.76
21.09
18.91
11.95
11.90
11.83
^{13}C NMR, 100 MHz, $CDCl_3$
N
N
N
N
N
N
O
O
NO_2
NO_2
H
H
anti,anti,syn
f1 (ppm)

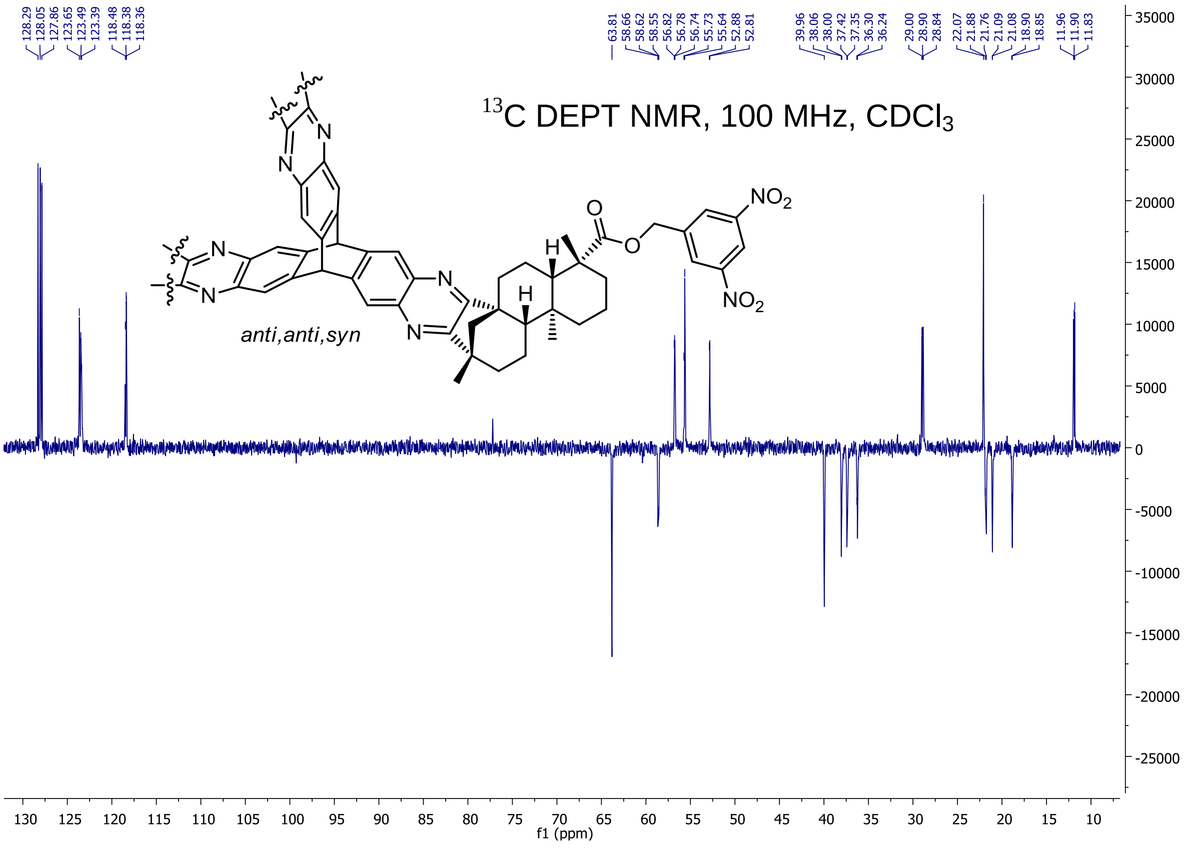

^{13}C DEPT NMR, 100 MHz, CDCl$_3$
anti,anti,syn
f1 (ppm)

Compound **15**:

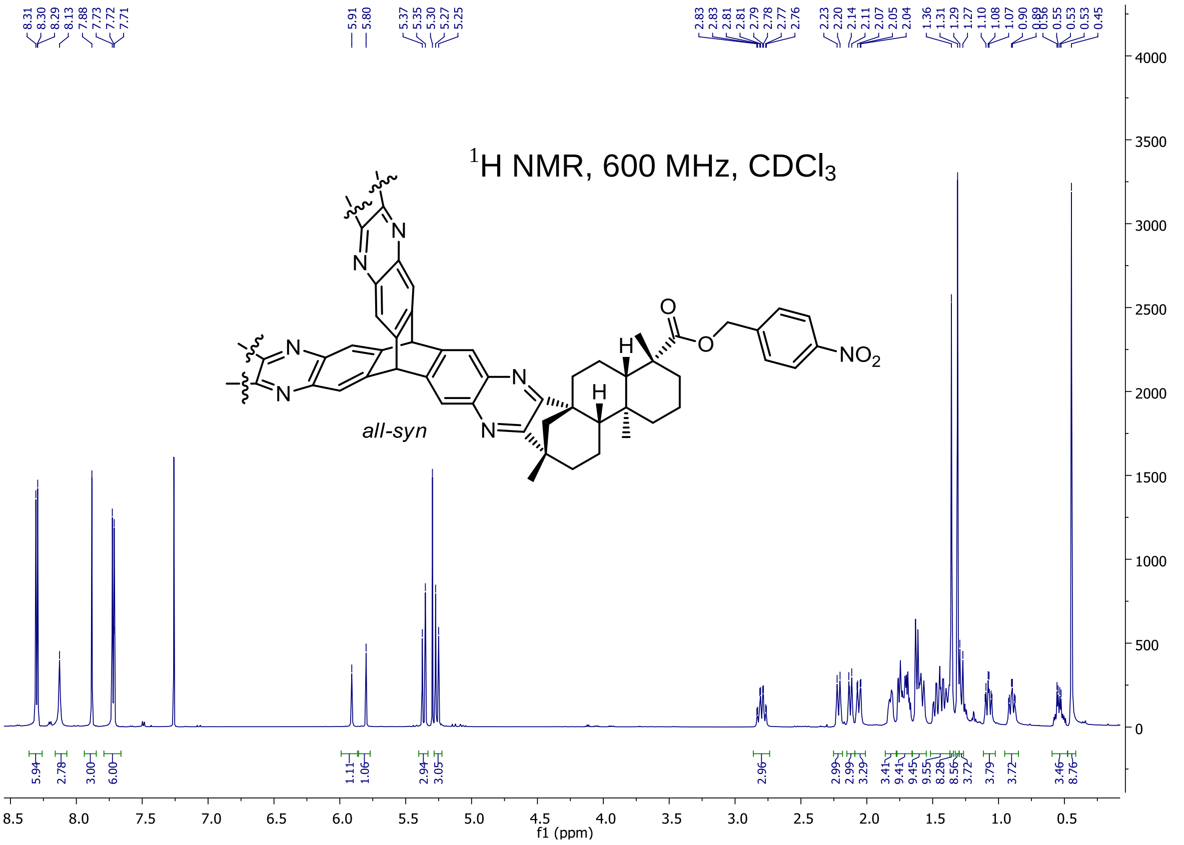

^{1}H NMR, 600 MHz, CDCl$_3$
all-syn
f1 (ppm)

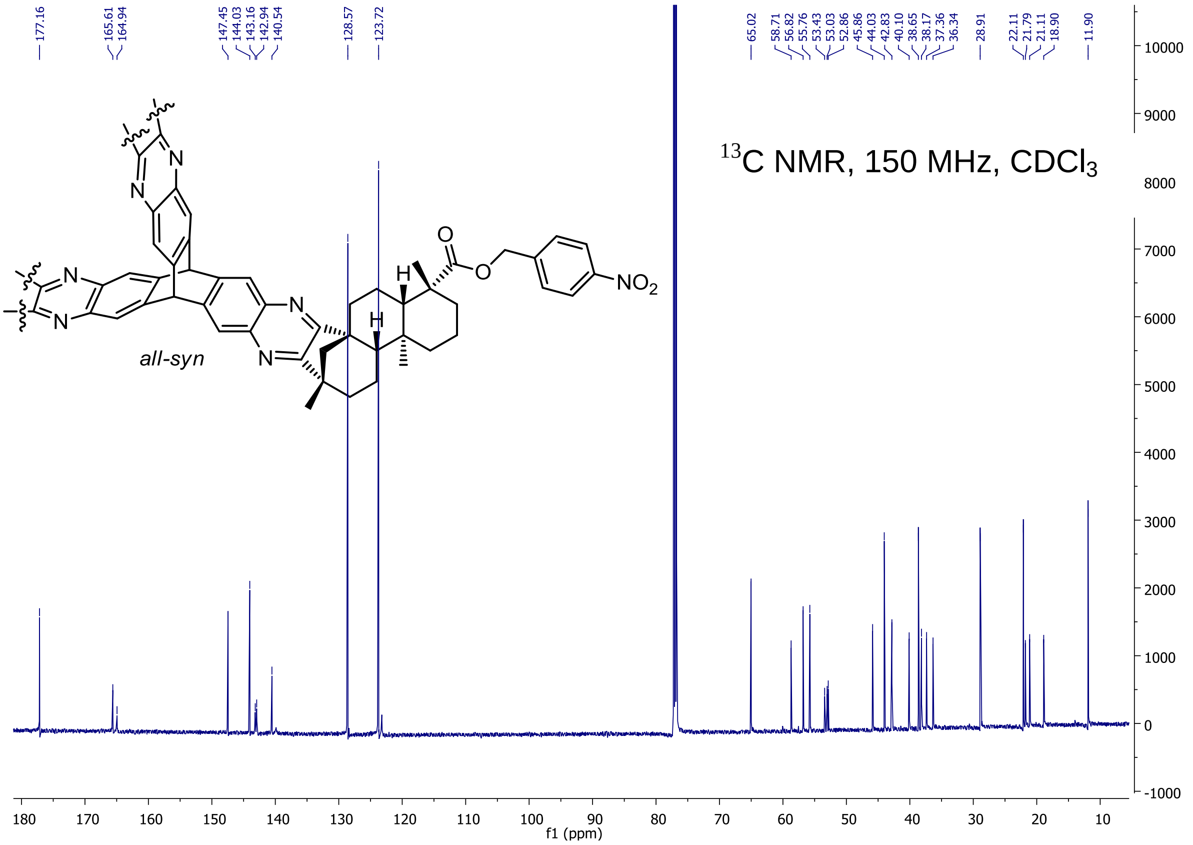

177.16
165.61
164.94
147.45
144.03
143.16
142.94
140.54
128.57
123.72
65.02
58.71
56.82
55.76
53.43
53.03
52.86
45.86
44.03
42.83
40.10
38.65
38.17
37.36
36.34
28.91
22.11
21.79
21.11
18.90
11.90
^{13}C NMR, 150 MHz, CDCl$_3$
all-syn
NO_2
f1 (ppm)

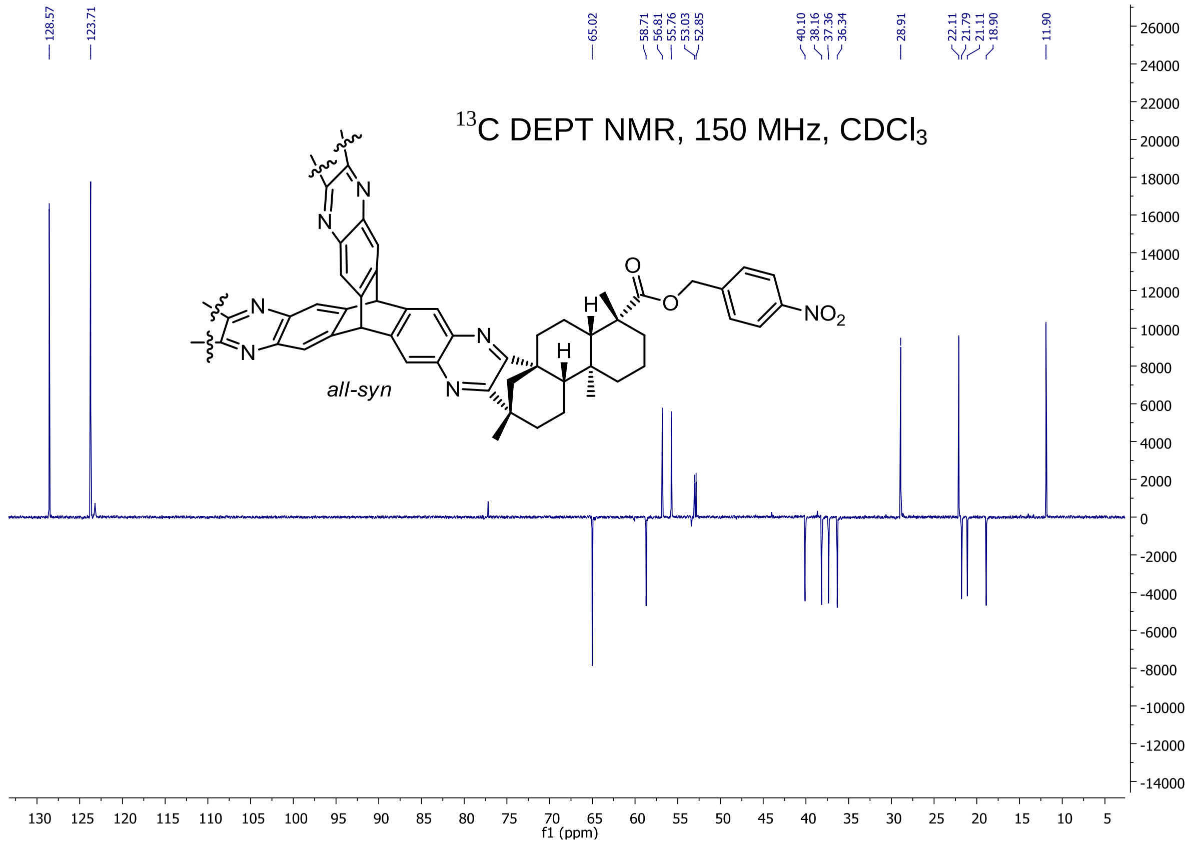

128.57
123.71
65.02
58.71
56.81
55.76
53.03
52.85
40.10
38.16
37.36
36.34
28.91
22.11
21.79
21.11
18.90
11.90
^{13}C DEPT NMR, 150 MHz, CDCl$_3$
all-syn
NO_2
f1 (ppm)

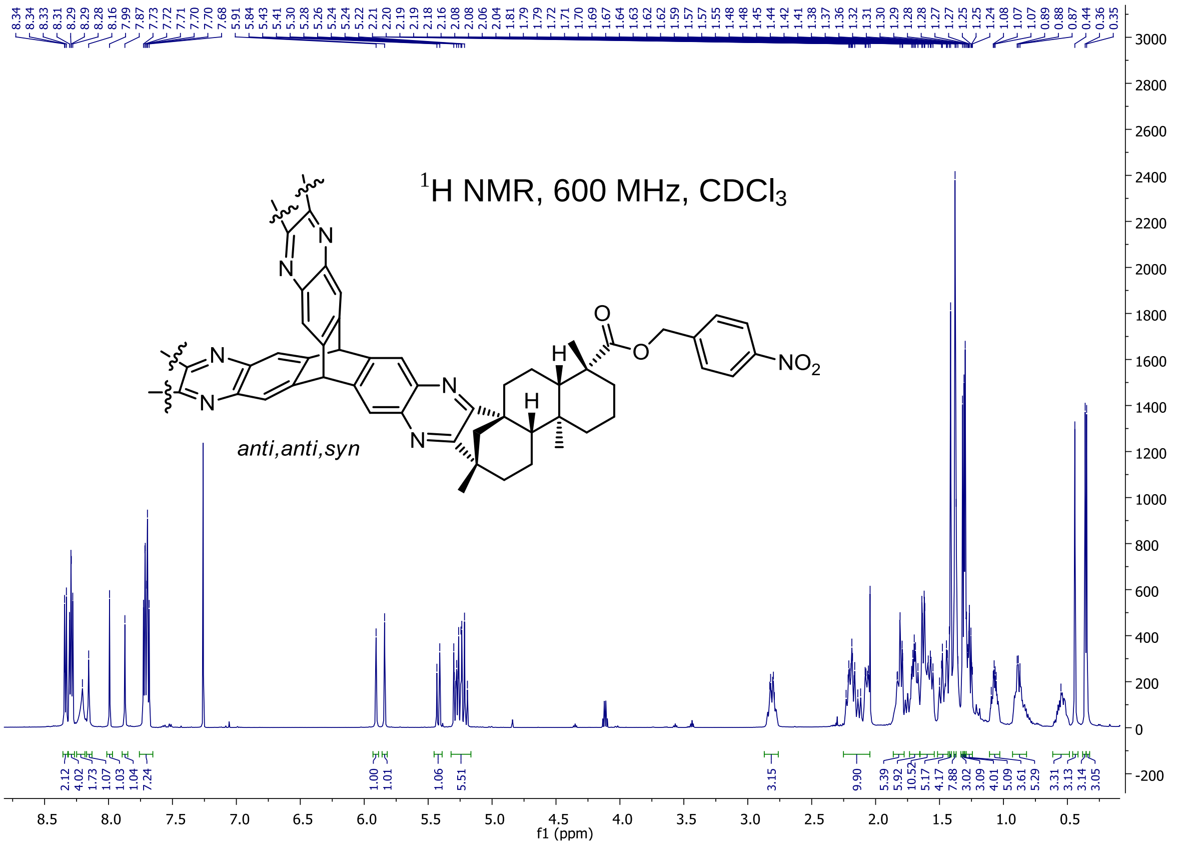

8.34 8.34 8.33 8.31 8.29 8.29 8.28 8.16 7.99 7.87 7.73 7.72 7.71 7.70 7.68 5.91 5.84 5.43 5.41 5.30 5.28 5.26 5.24 5.24 5.22 2.21 2.20 2.19 2.19 2.18 2.16 2.08 2.08 2.06 2.04 1.81 1.79 1.72 1.71 1.70 1.69 1.67 1.64 1.63 1.62 1.62 1.59 1.57 1.55 1.48 1.45 1.44 1.42 1.41 1.38 1.37 1.36 1.32 1.31 1.30 1.29 1.28 1.28 1.27 1.27 1.25 1.25 1.24 1.08 1.07 1.07 0.89 0.88 0.87 0.44 0.36 0.35
^{1}H NMR, 600 MHz, CDCl$_3$
N
N
N
N
N
N
H
H
O
O
NO$_2$
anti,anti,syn
2.12 4.02 1.73 1.07 1.03 1.04 7.24 1.00 1.01 1.06 5.51 3.15 9.90 5.39 5.92 10.52 5.17 4.17 7.88 3.02 3.09 4.01 5.09 3.61 5.29 3.31 3.13 3.14 3.05
8.5 8.0 7.5 7.0 6.5 6.0 5.5 5.0 4.5 4.0 3.5 3.0 2.5 2.0 1.5 1.0 0.5
f1 (ppm)
3000 2800 2600 2400 2200 2000 1800 1600 1400 1200 1000 800 600 400 200 0 -200

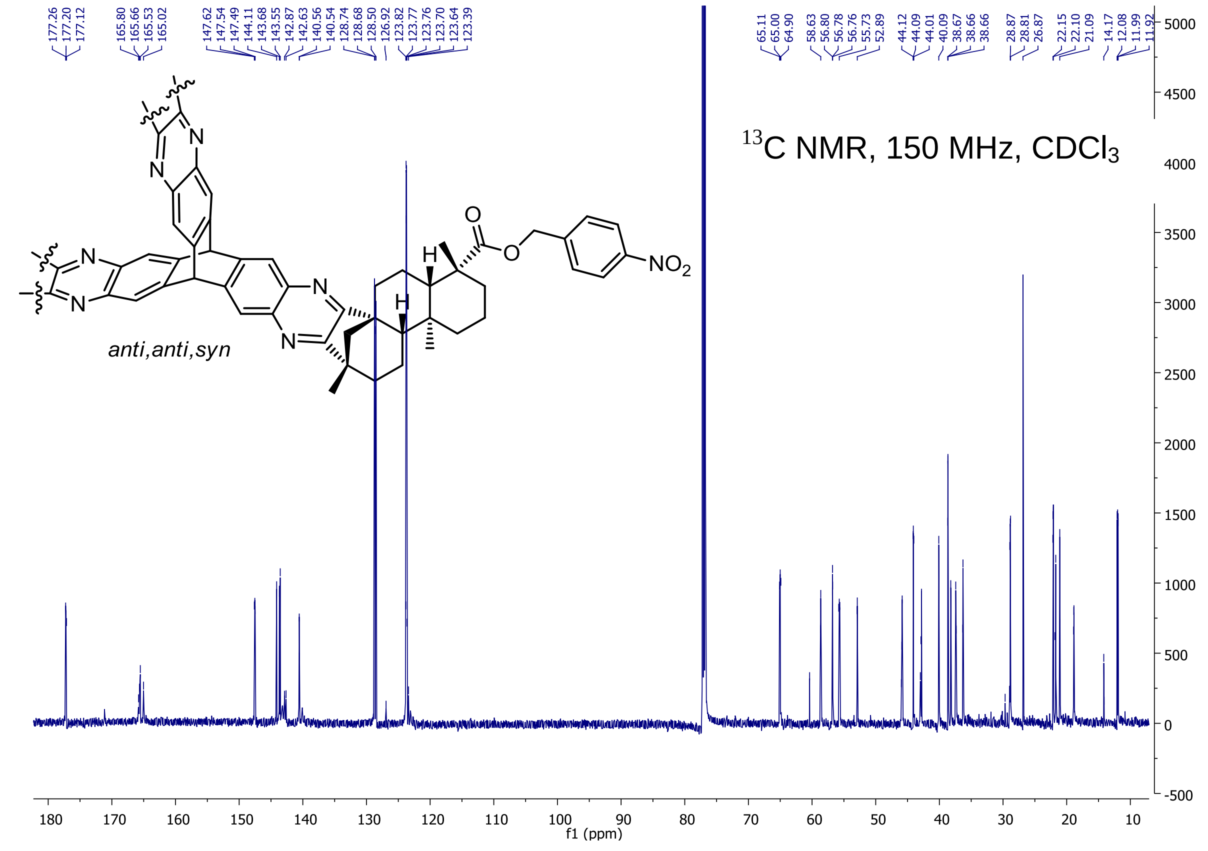

177.26 177.20 177.12 165.80 165.66 165.53 165.02 147.62 147.54 147.49 144.11 143.68 143.55 142.87 142.63 140.56 140.54 128.74 128.68 128.50 126.92 123.82 123.77 123.76 123.70 123.64 123.39 65.11 65.00 64.90 58.63 56.80 56.78 56.76 55.73 52.89 44.12 44.09 44.01 40.09 38.67 38.66 38.66 28.87 28.81 26.87 22.15 22.10 21.09 14.17 12.08 11.99 11.92
^{13}C NMR, 150 MHz, CDCl$_3$
N
N
N
N
N
N
H
H
O
O
NO$_2$
anti,anti,syn
180 170 160 150 140 130 120 110 100 90 80 70 60 50 40 30 20 10
f1 (ppm)
5000 4500 4000 3500 3000 2500 2000 1500 1000 500 0 -500

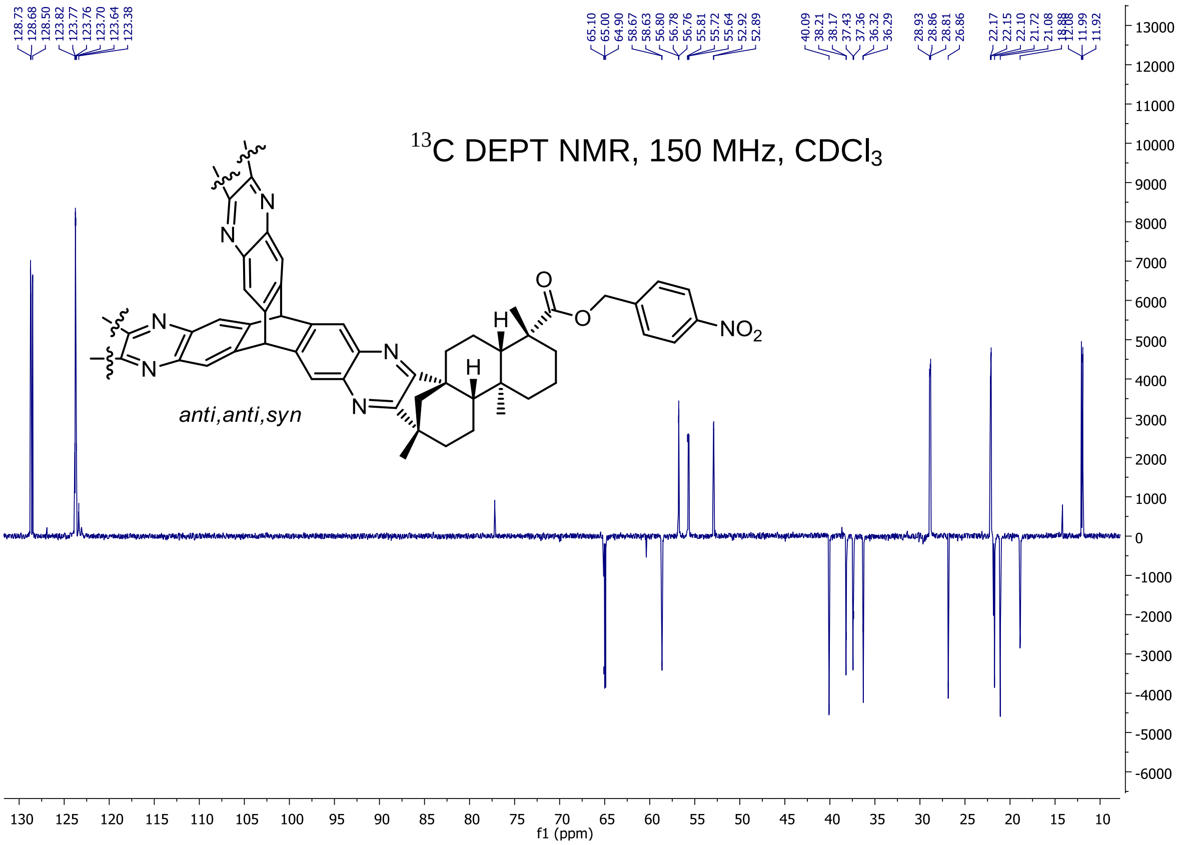

Compound **16**: [* Contamination with *p*-toluidine, see characterization]

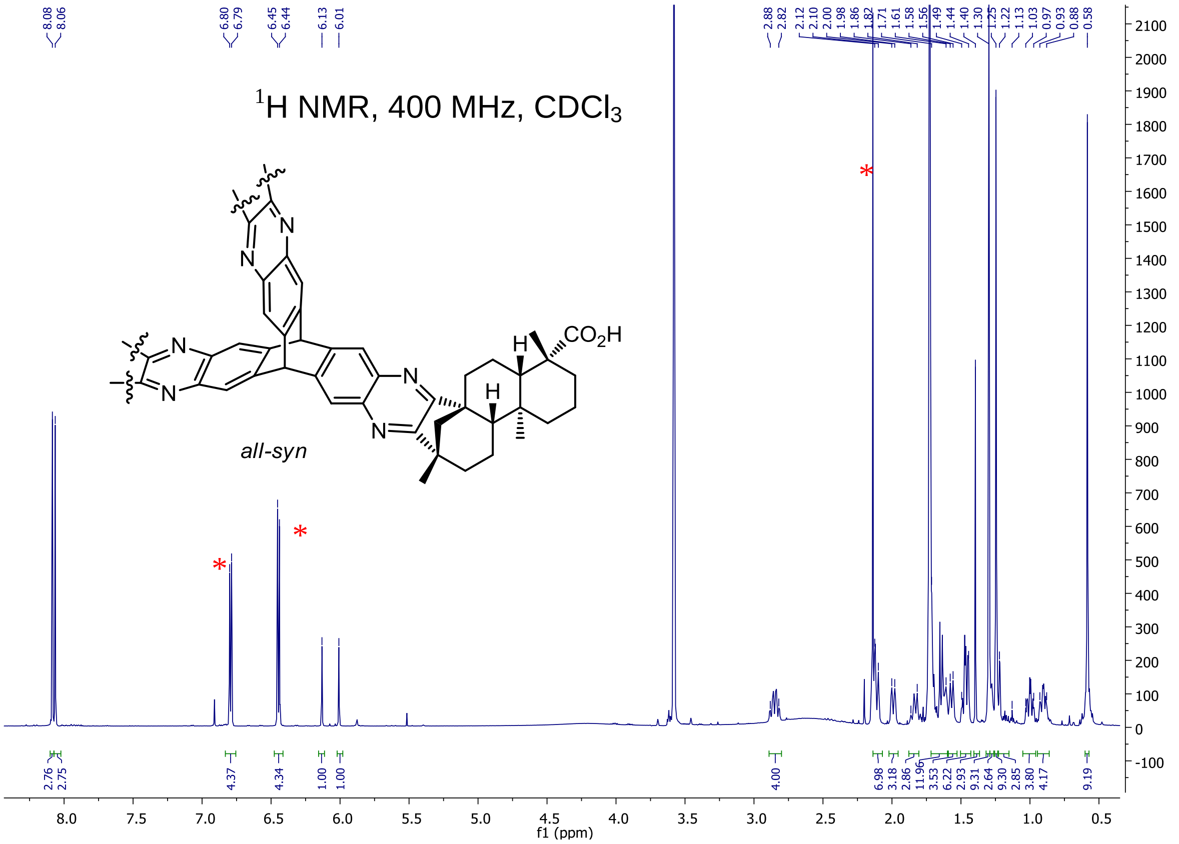

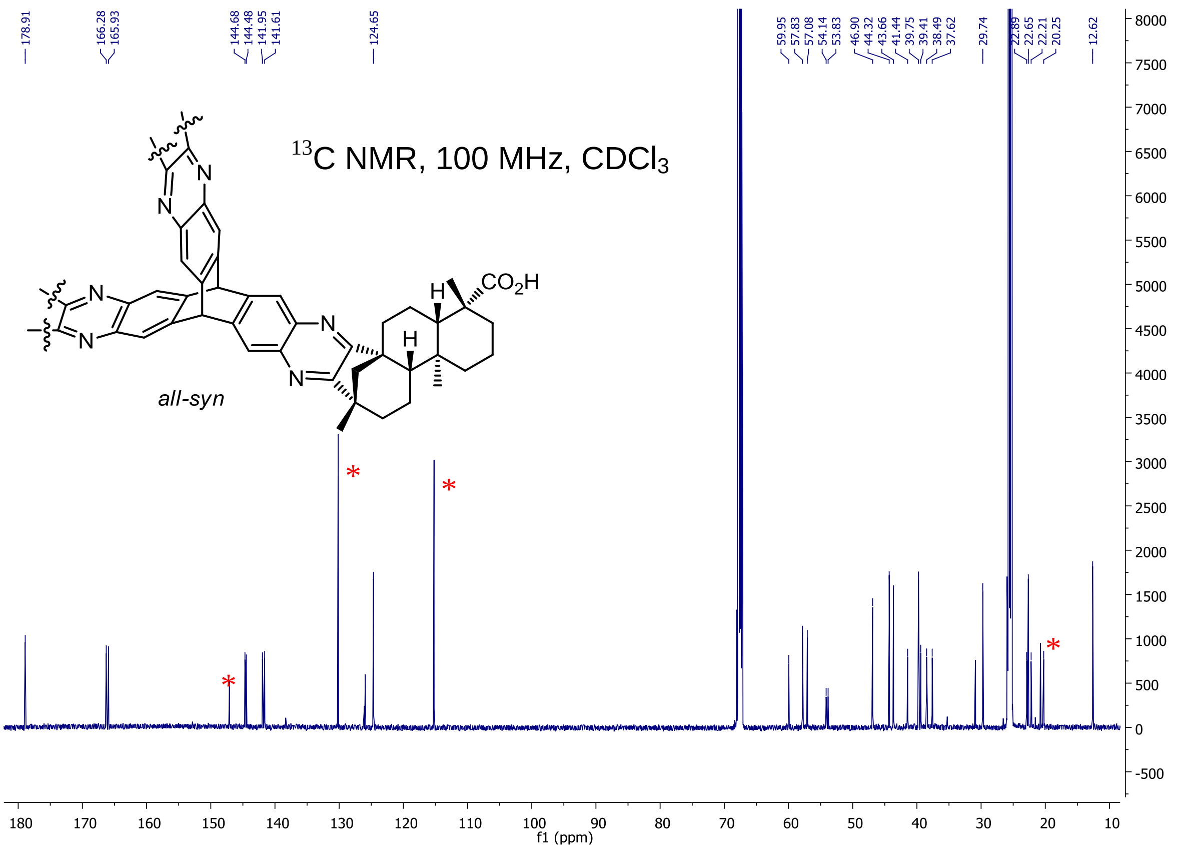

^{13}C NMR, 100 MHz, CDCl$_3$
all-syn
CO_2H
178.91
166.28
165.93
144.68
144.48
141.95
141.61
124.65
59.95
57.83
57.08
54.14
53.83
46.90
44.32
43.66
41.44
39.75
39.41
38.49
37.62
29.74
22.89
22.65
22.21
20.25
12.62
f1 (ppm)

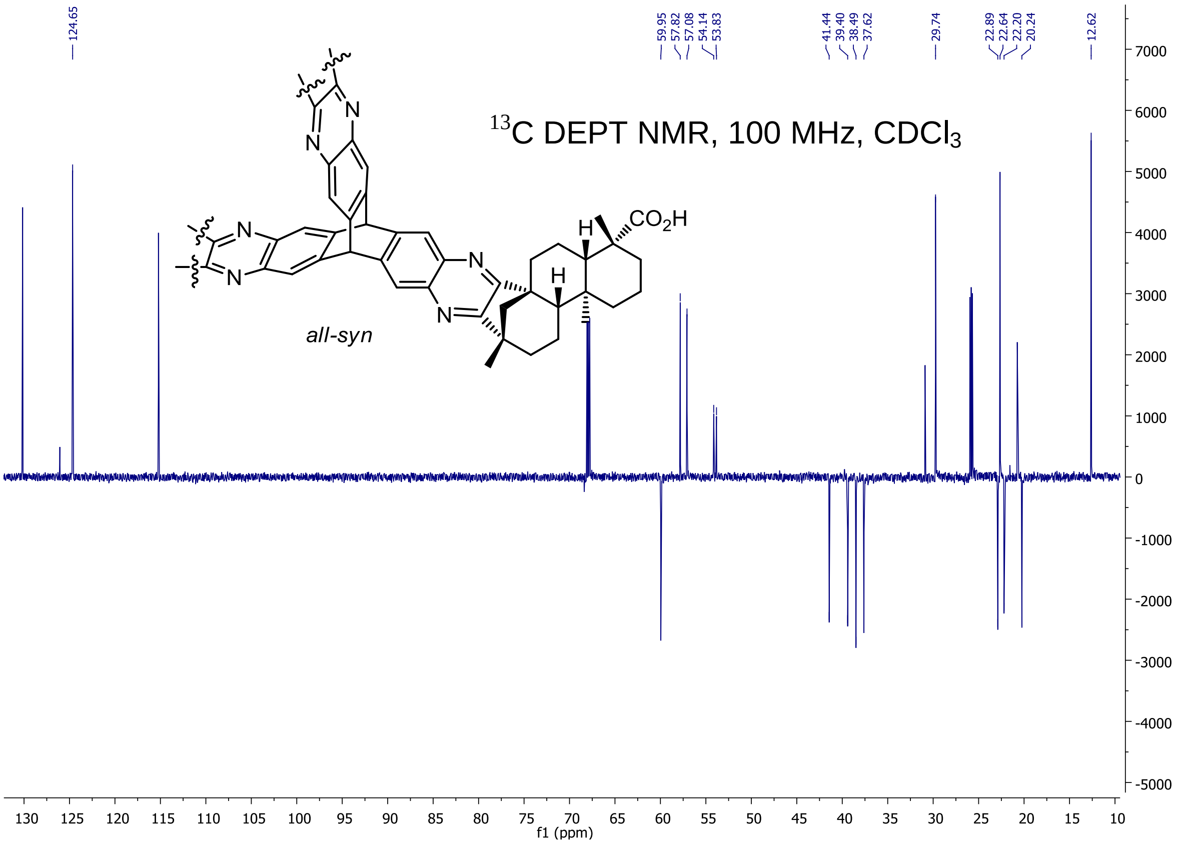

^{13}C DEPT NMR, 100 MHz, CDCl$_3$
all-syn
CO_2H
124.65
59.95
57.82
57.08
54.14
53.83
41.44
39.40
38.49
37.62
29.74
22.89
22.64
22.20
20.24
12.62
f1 (ppm)

Compound **17**:

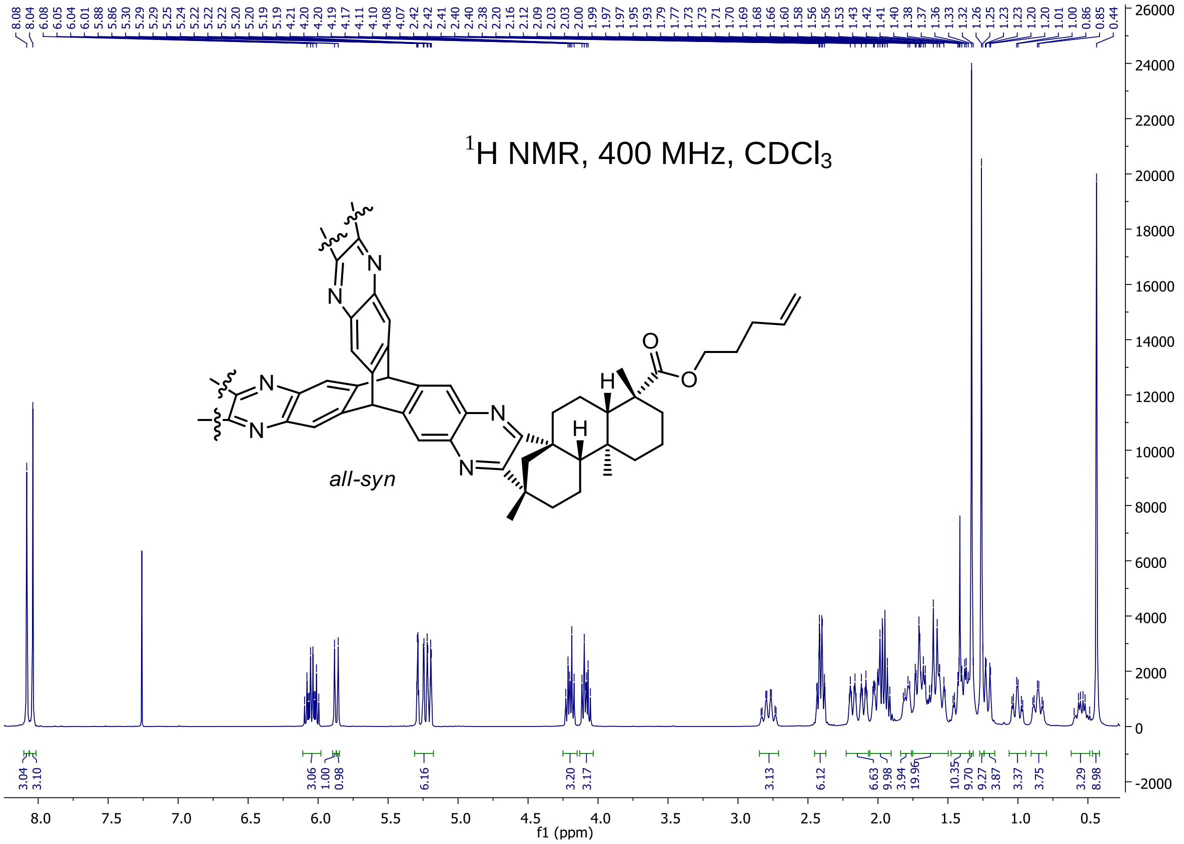

1H NMR, 400 MHz, CDCl3
all-syn
f1 (ppm)

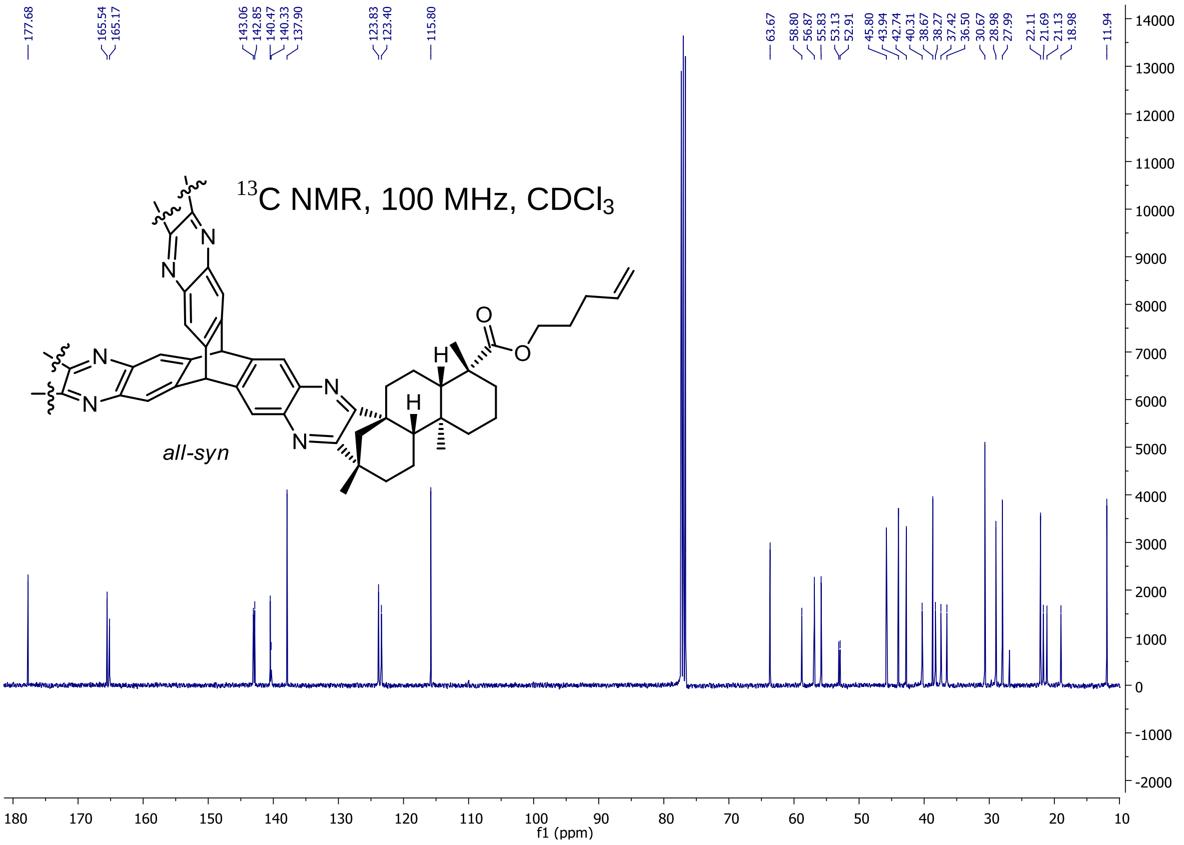

13C NMR, 100 MHz, CDCl3
all-syn
f1 (ppm)

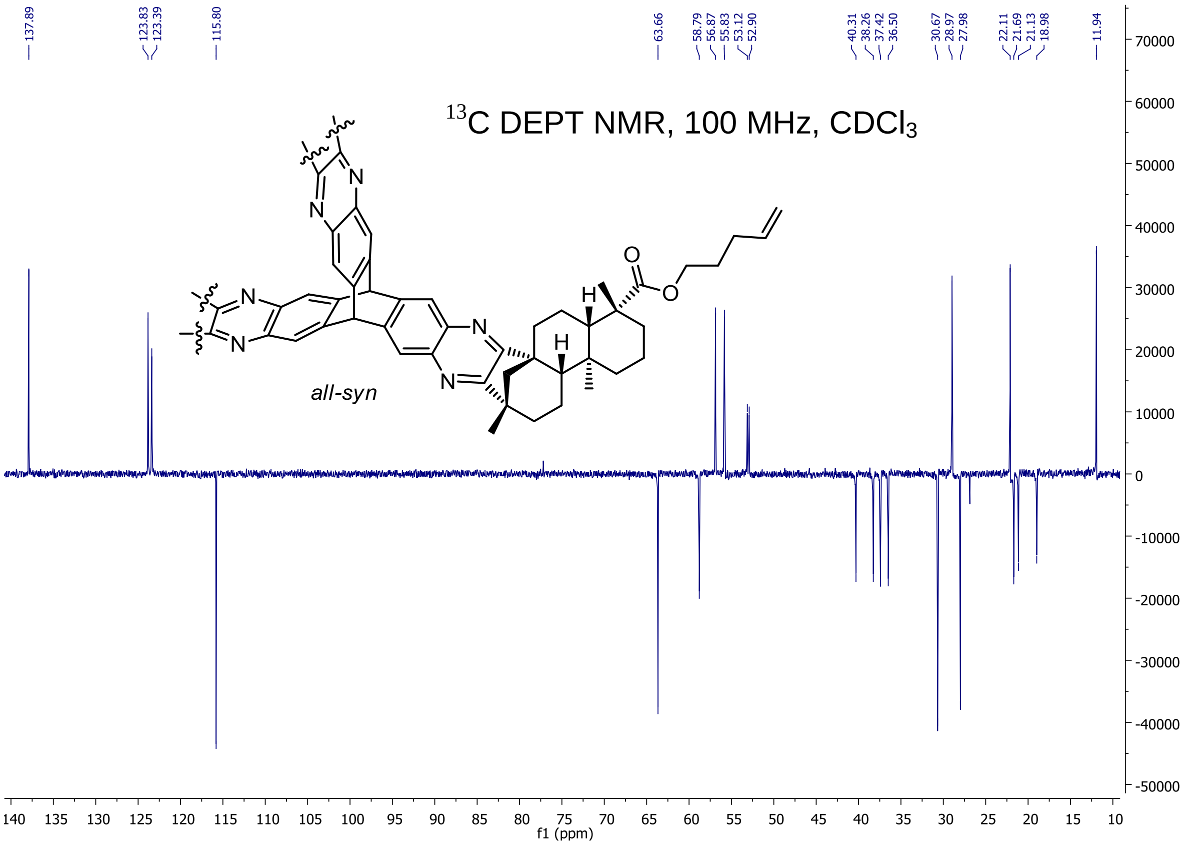
13C DEPT NMR, 100 MHz, CDCl3
all-syn
137.89
123.83
123.39
115.80
63.66
58.79
56.87
55.83
53.12
52.90
40.31
38.26
37.42
36.50
30.67
28.97
27.98
22.11
21.69
21.13
18.98
11.94
f1 (ppm)

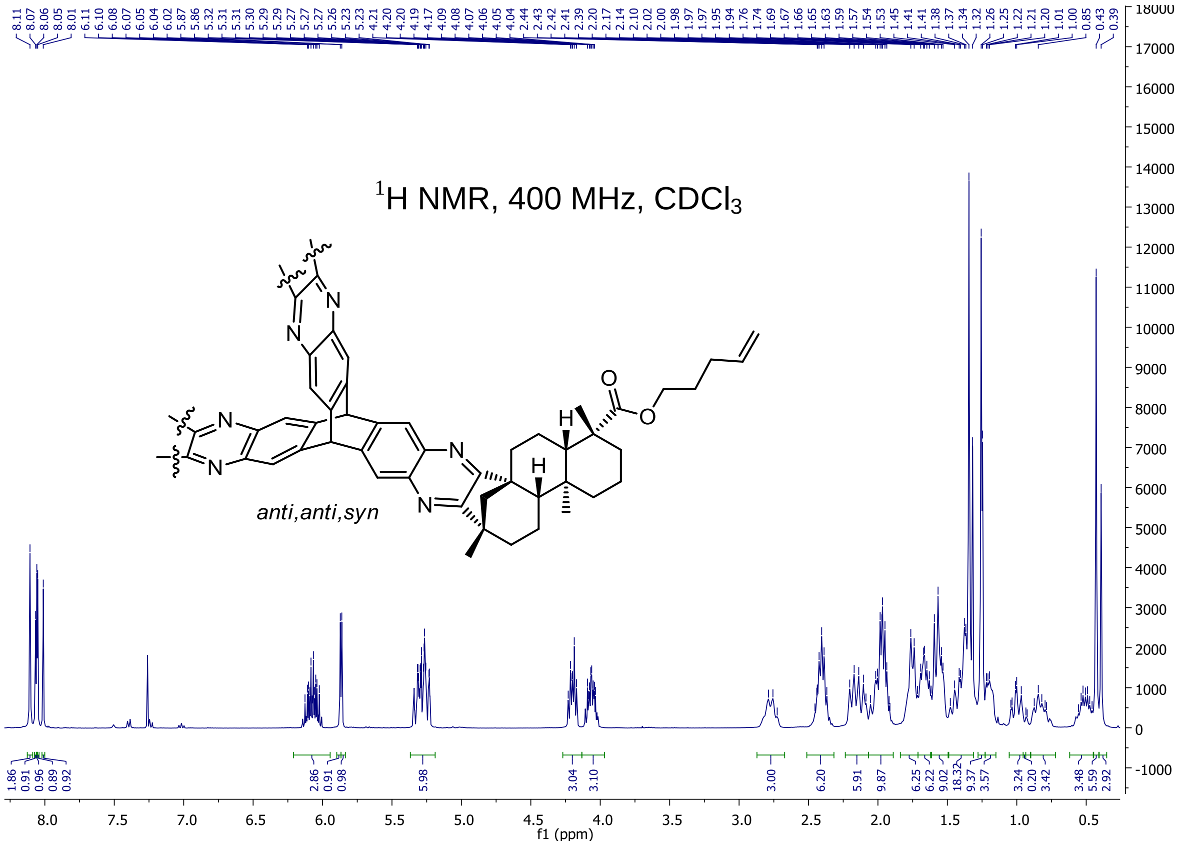
1H NMR, 400 MHz, CDCl3
anti,anti,syn
f1 (ppm)

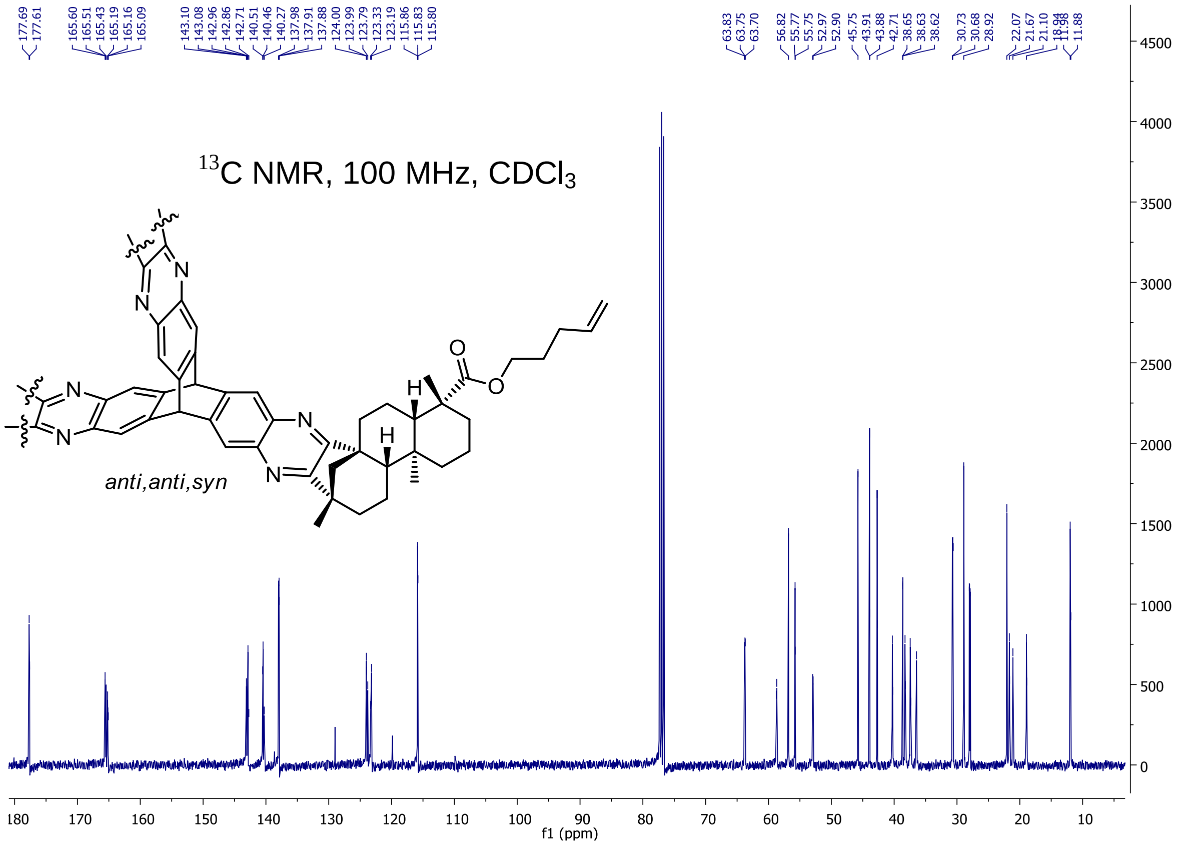

^{13}C NMR, 100 MHz, CDCl$_3$
anti,anti,syn
f1 (ppm)

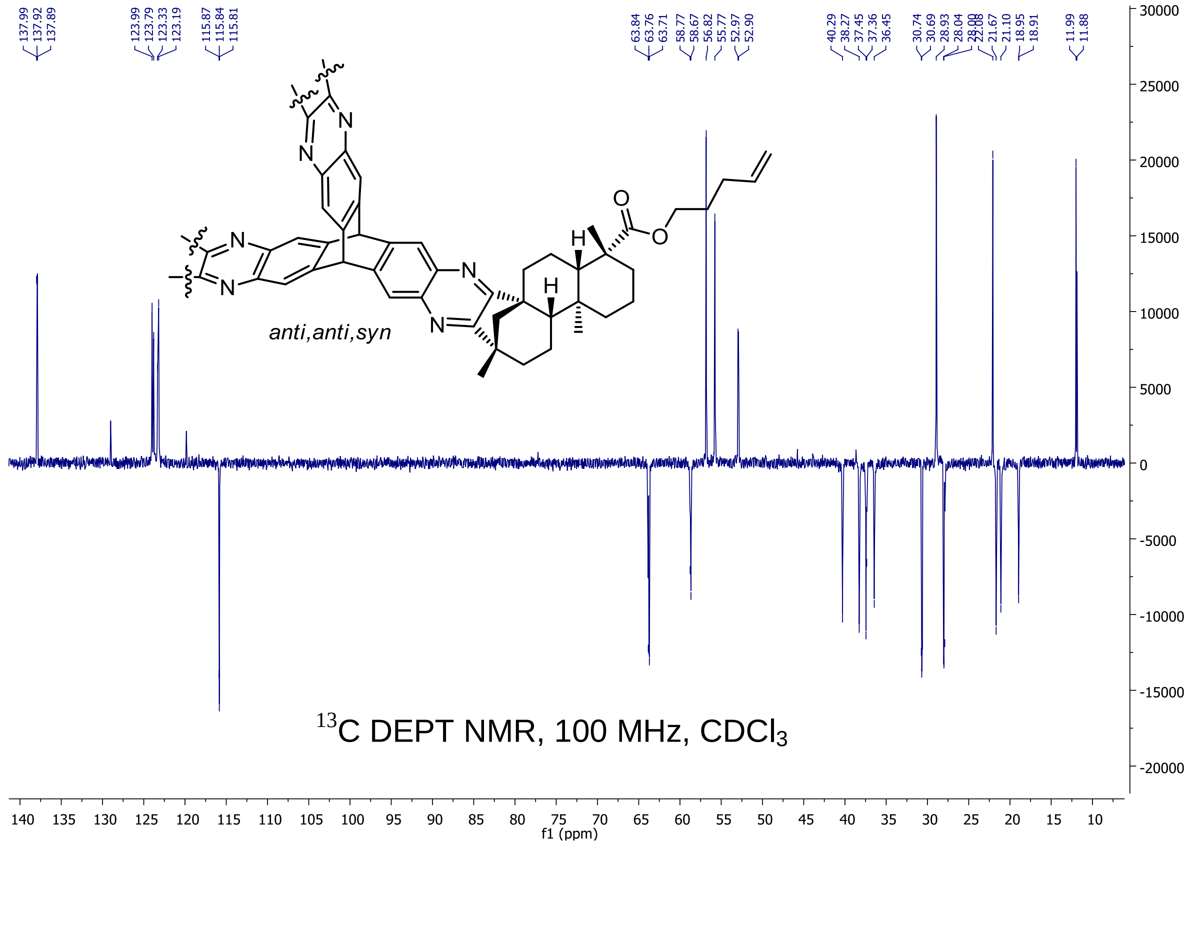

^{13}C DEPT NMR, 100 MHz, CDCl$_3$
anti,anti,syn
f1 (ppm)

D. Evaluation of affinity

QCM setup

The resonance frequency of thickness-shear resonators, like the employed quartz crystal microbalances, is largely influenced by the oscillating mass. The sensitivity of the quartz resonator is influenced by several environmental factors and is described by the Sauerbrey equation (formula 1),[5] wherein A is the oscillating area, N the frequency constant and ρ the density of the quartz material. A variation in the oscillating mass Δm directly results in a linear shift Δf_0 of the fundamental resonance frequency f_0.

$$\Delta f_0 = -\frac{f_0^2}{N \cdot \rho \cdot A} \cdot \Delta m \qquad 1$$

The Sauerbrey equation applies to homogeneous films with nearly the same viscoelastic properties as the quartz crystal so that no damping of the oscillator appears. Also, the maximum frequency shift has to be lower than 2% of the fundamental frequency, in this case 2000 kHz. This correlation is an easy way to determine affinities from various analytes towards selected affinity materials [6].

Coating protocol

The coating of the quartz crystal microbalances is performed using an electrospray protocol [7]. This particular method is well established and allows the continuously monitoring of mass deposition onto the quartz upon spraying process. The electrospray solutions are prepared at concentrations of approx. 0.1 mg/mL in a 9:1 mixture of tetrahydrofuran/methanol.

The experimental setup for the coating unit is schematically displayed in Figure S1. The solution for coating is placed in a glass syringe equipped with metal cannula. The metallic part of the needle is contacted with an applied voltage of 5 kV relative to a counter electrode which is represented by the electrode of the QCM to be coated (distance 0.15 m needle tip/electrode). The cannula represents the anode. A constant delivery of the solution during the coating process is achieved by using a syringe pump (5 µL/min, Figure S1/2). The coating process is monitored by measuring the frequency shift of the QCM. Since it is not possible to determine the

thickness of the deposited film directly, the amount of affinity material on the quartz device is given by the frequency shift. For screening purposes, all compounds are deposited on 195 MHz QCMs until a frequency shift of 50 kHz is reached. This shift corresponds to a mass of approx. 10.4 ng of the deposited material on the electrodes.

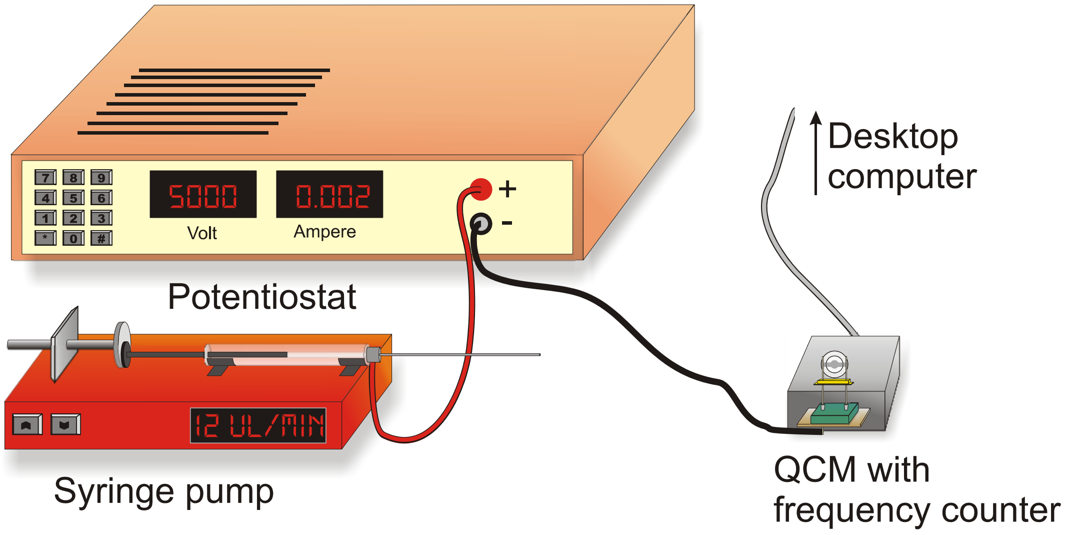

Figure S1: Setup of the coating unit for electrospray.

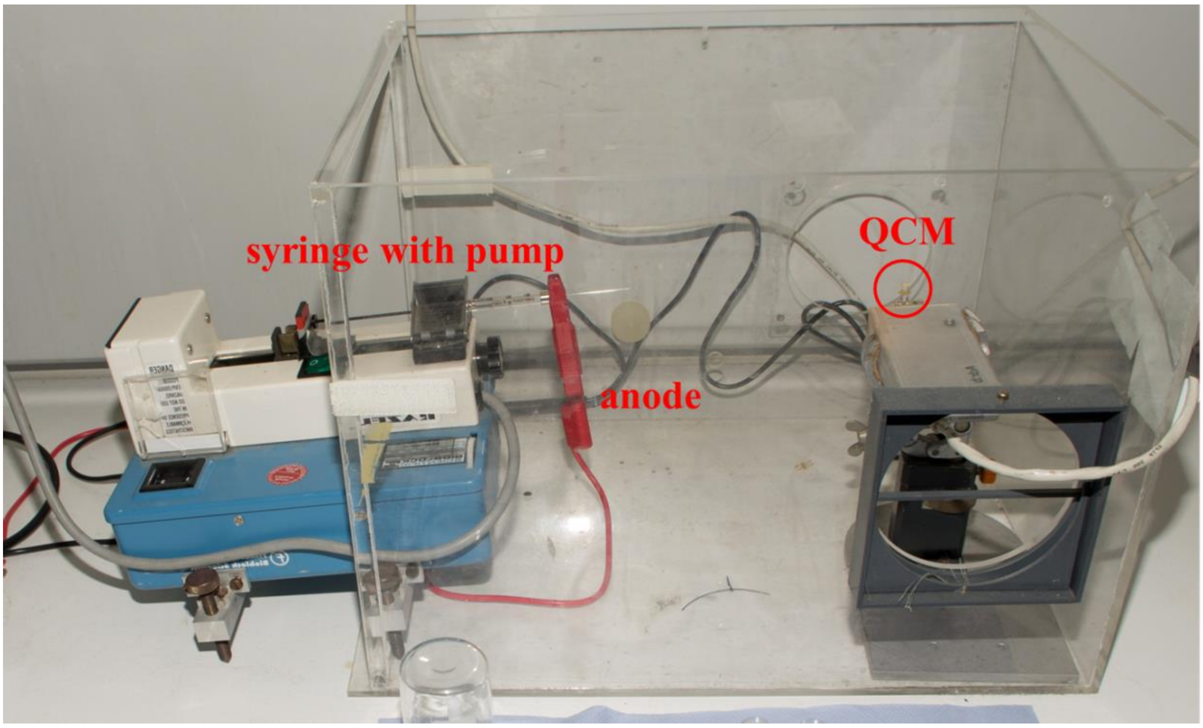

Figure S2: The electrospray setup in the lab.

Setup for measurement of affinity

For determination of affinities precise conditions and concentration of analytes are required. Therefore, a “closed” system connected to a gas mixing unit is used (Figure S2). In this gas mixing unit, the inert nitrogen flow is divided into two streams, both controlled by an individual mass flow controller (MFC) from the Brooks Instrument company (Model 5050S). Flow 1 remains unchanged in temperature and composition and is used as gas source for dilution purposes, whereas, flow 2 is led through an interchangeable analyte-reservoir which is adjusted to 293.1±0.2 K. The analyte-saturated gas flow 2 is recombined with the pure inert gas flow 1 and led to the measuring chamber. The overall gas flow is set to 200 mL/min. By carefully controlling the flow of the both streams by the MFCs, it is possible to produce gas mixtures with a concentration of 1 to 100% of the vapor pressure of the pure analyte at 293.1±0.2 K.

The central part of the screening setup is the measuring cell which is connected to the gas mixing unit and placed in a temperature controlled environment. We employed a slightly modified GC oven (Hewlett Packard, Palo Alto, CA, USA. Type: HP 5890; Figure S4). The cell is kept constantly at 308 K±0.5 K to exclude temperature influences and to prevent condensation effects within the cell. The cell is designed to operate up to 12 QCM in a parallel fashion.

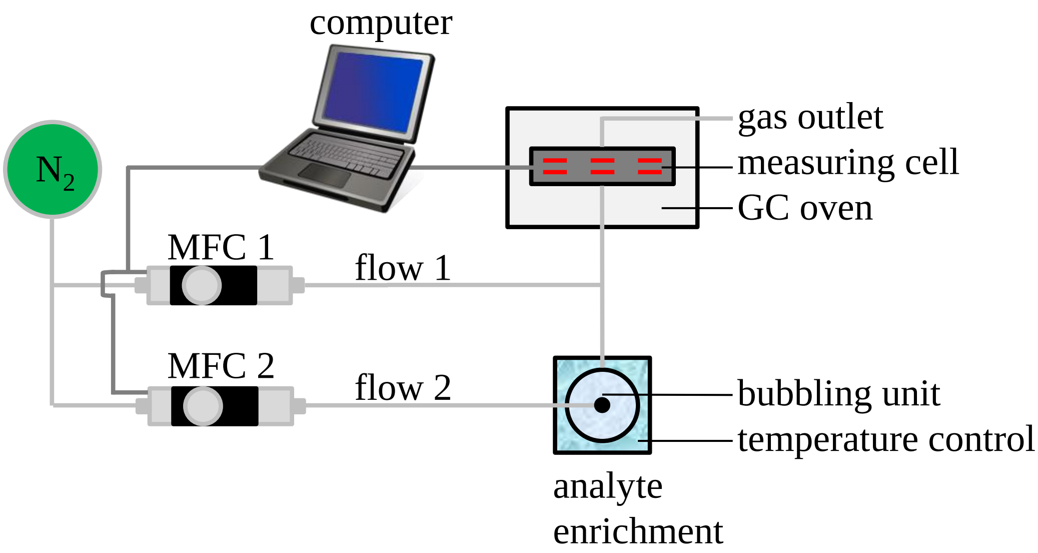

Figure S3: Setup for measurement.

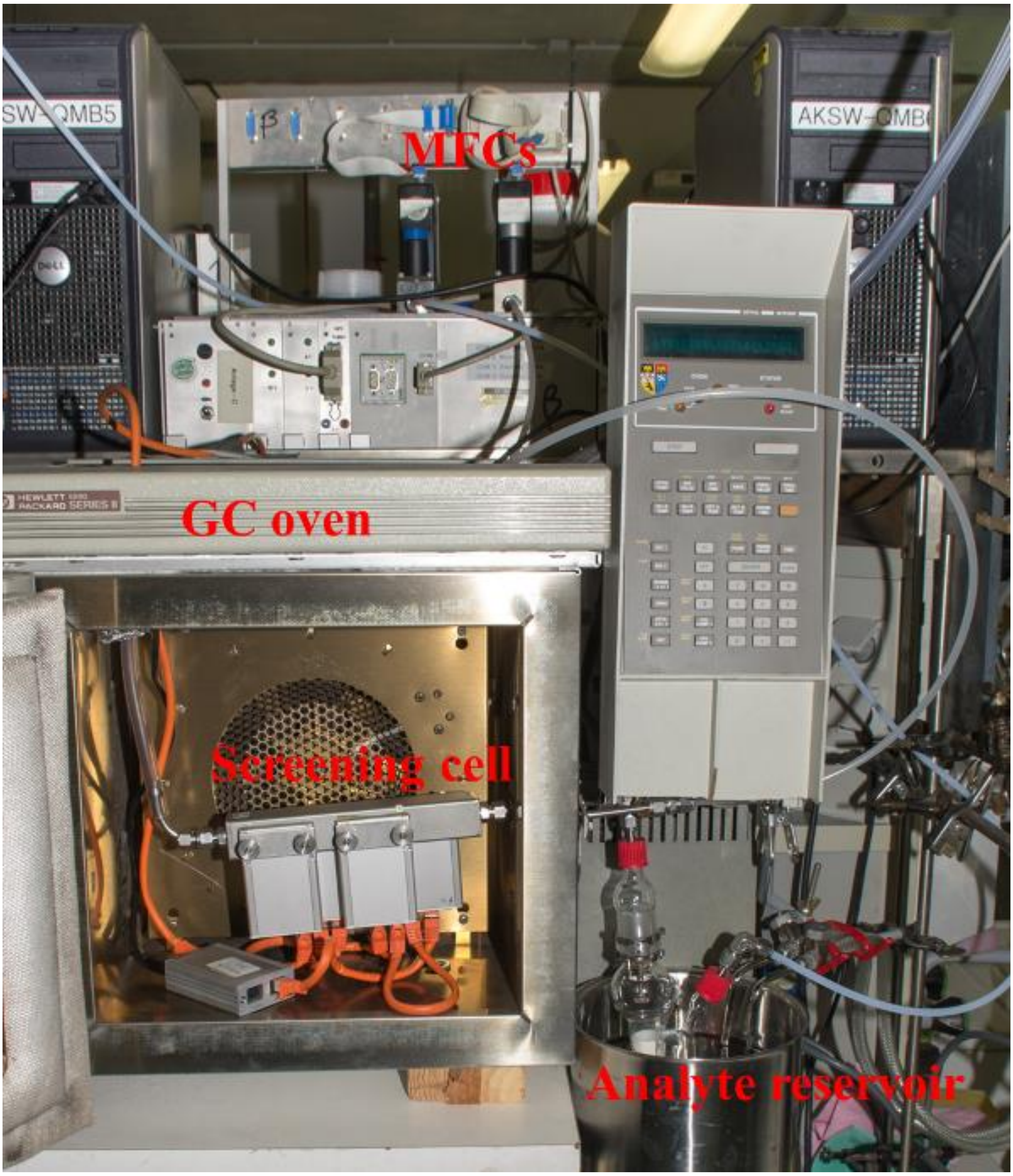

Figure S4: The experimental measurement setup.

Evaluation of the affinity materials

The following example will show the procedure for determination of affinity. The applied affinity material in this example is the triptycene derivative *anti,anti,syn*-**8** and employed analyte is pseudocumene. This procedure is carried out for every affinity material with the individual analyte. In Figure Figure S5 the primary data of a typical measurement for an analyte are depicted. The sensor responses (Figure S5, ΔF_c) were determined by referencing the frequency of the QCM (Figure S5, a) just prior to

the admittance of analyte into the chamber to the frequency in equilibrium (Figure S5, b). The given recovery time between two concentration steps was 1500 seconds in each experiment. The given adsorption time was 1000 seconds for the first five concentration steps and 2000 seconds for the last three.

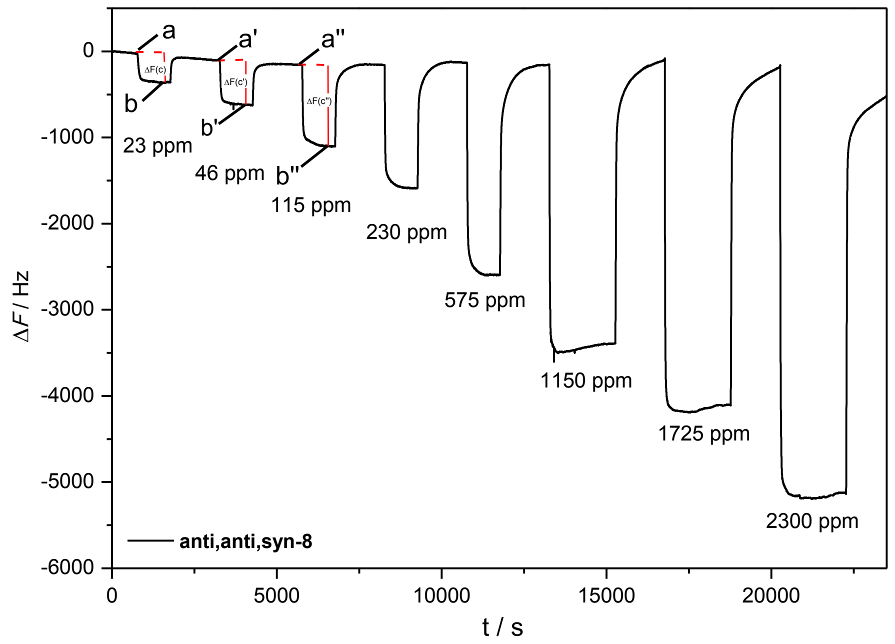

Figure S5: Frequency shifts for different pseudocumene concentrations (primary data, affinity material *anti,anti,syn*-**8**).

By plotting the frequency shift vs. the ethanol concentration the constants of the *Langmuir* adsorption isotherm was determined (Figure S6). The graph is obtained by fitting to equation 2.

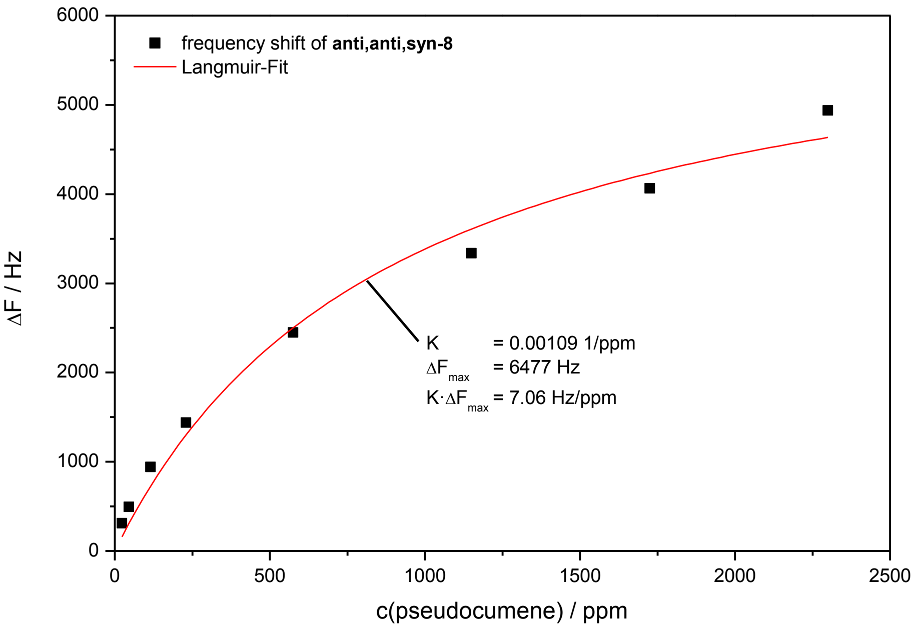

Figure S6: Determination of the affinity from the frequency shifts for different pseudocumene concentrations (affinity material *anti,anti,syn*-**8**)

The slope of the linear part of the *Langmuir* equation is the product of the *Langmuir* constants in equation 2.

$$\Delta F = \frac{\Delta F_{max} \cdot K \cdot c_{anayte}}{1 + K \cdot c_{anayte}} \qquad 2$$

Since the resulting number is a general information about the affinity of a film or porous material to a respective analyte, $\Delta F_{max} \cdot K$ will be used as the affinity within this study.

Table S1: Overview of screened analytes.

analyte	vapor pressure at 20 °C [ppm]	applied concentration range [ppm][a]
**benzene**	99458[8]	99458 - 994
**toluene**	29331[8]	29331 - 293
**water**	23000[9]	23000 - 230
**H_2O_2**	14500[10]	7250 - 73
**p-xylene**	8700[9]	8700 - 87
**m-xylene**	8000 [9]	8000 - 80
**o-xylene**	7000 [9]	7000 - 70
**mesitylene**	2800 [9]	2800 - 28
**pseudocumene**	2300[8]	2300 - 23

[a] For all analytes eight concentrations between 1 % and 100 % of the saturation concentration in the gas phase at 20 °C were chosen.

Screening results

In order to obtain comparable results, the coating of the QCM was always made with the same mass of affinity material (50 kHz which corresponds to 10.4 ng). By that, a comparable film thickness is achieved. In the following table, the affinity is presented for the analytes.

Table S2: Affinities of the affinity materials all-*syn*-**3**, **7**, **8** and **14**.

analyte	*all-syn*-3 [Hz/ppm]	7 [Hz/ppm]	*all-syn*-8 [Hz/ppm]	anti,anti,syn-8 [Hz/ppm]	*all-syn*-14 [Hz/ppm]	anti,anti,syn-14 [Hz/ppm]
**benzene**	0,1238	0,1110	0,0729	0,1410	0,0739	0,0773
**toluene**	0,453	0,404	0,204	0,430	0,395	0,240
**water**	0,23	0,54	0,53	0,46	1,23	0,90
**H_2O_2**	0,29	0,66	0,75	1,69	1,60	3,33
***p*-xylene**	1,43	1,12	0,70	1,90	1,43	0,71
***m*-xylene**	1,73	1,64	0,94	1,05	1,44	0,81
***o*-xylene**	1,98	1,47	0,85	3,34	1,34	0,94
**mesitylene**	4,11	2,88	1,35	4,96	4,05	1,86
**pseudocumene**	5,95	4,02	2,79	7,06	6,56	4,46

Table S3: Affinities of the affinity materials **15**, **17** and **19**.

analyte	*all-syn*-15 [Hz/ppm]	anti,anti,syn-15 [Hz/ppm]	*all-syn*-17 [Hz/ppm]	anti,anti,syn-17 [Hz/ppm]	19 [Hz/ppm]
**benzene**	0,0751	0,0756	0,0605	0,072	0,0571
**toluene**	0,223	0,239	0,175	0,228	0,122
**water**	1,00	0,73	1,20	0,67	0,37
**H_2O_2**	1,33	2,43	2,80	2,54	1,49
***p*-xylene**	1,20	0,80	0,60	0,67	0,43
***m*-xylene**	1,25	0,80	0,68	0,75	0,44
***o*-xylene**	1,17	0,93	0,80	0,85	0,53
**mesitylene**	3,48	2,17	1,52	1,65	0,77
**pseudocumene**	5,61	4,1	3,26	3,75	2,69

E. Structure of the compared organic cage compound

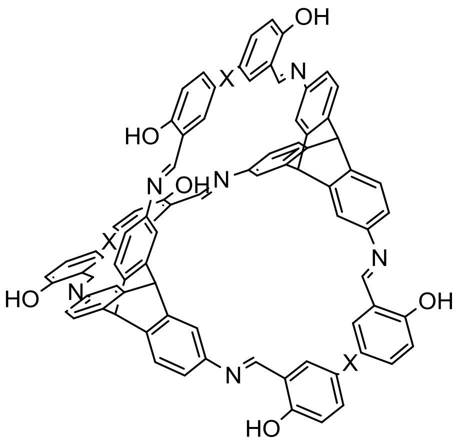

(X = -C_2H_4-)

E. References

1. Bomkamp, M.; Artiukhov, A.; Kataeva, O.; Waldvogel, S. R. *Synthesis* **2007**, 1107–1114.

2. a) Neubig, B.; Briese, W. *Das große Quarzkochbuch*, Franzis, Feldkirchen, **1997**; b) Urbakh, M.; Tsionsky, V.; Gileadi, E.; Daikhin, L. in *Springer Series on Chemical Sensors and Biosensors. Piezoelectric Sensors*, Vol. 5 (*Ed: Wolfbeis, O. S.; Vol. Eds: Steinem, C.; Janshoff, A.*), Springer, Berlin, Heidelberg, **2007**,111−149.

3. Bomkamp, M.; Gottfried, K.; Kataeva, O.; Waldvogel, S. R. *Synthesis* **2008**, 1443–1447.

4. Mastalerz, M.; Sieste, S.; Cenić, M.; Oppel, I. M. *J. Org. Chem.* **2011**, *76*, 6389–6393.

5. Sauerbrey, G. *Zeitschrift für Physik* **1959**, *155*, 206–222.

6. Mirsky, V. M. *Artificial receptors for chemical sensors*, Wiley-VCH, Weinheim, **2011**.

7. a) Heil, C.; Windscheif, G. R.; Braschohs, S.; Flörke, J.; Gläser, J.; Lopez, M.; Müller-Albrecht, J.; Schramm, U.; Bargon, J.; Vögtle, F. *Sens. Actuators. B* **1999**, *61*, 51–58; b) Fenn, J. B. *Angew. Chem.* **2003**, *115*, 3999−4024; *Angew. Chem. Int. Ed.* **2003**, *42*, 3871−3894.

8. *Aldrich Handbook - a Catalog of Fine Chemicals and Laboratory Equipment,* Aldrich, **2003**.

9. *Chemicals & Reagents,* Merck KGaA, Darmstadt, **2011**.

10. Schumb, W.; Scatterfield, C.; Wentworth, R. *Hydrogen Peroxide*, Reinhold Publishing Corporation, New York **1955**.
